# Anti-*Trypanosoma cruzi* Activity, Mutagenicity, Hepatocytotoxicity and Nitroreductase Enzyme Evaluation of 3-Nitrotriazole, 2-Nitroimidazole and Triazole Derivatives

**DOI:** 10.3390/molecules28227461

**Published:** 2023-11-07

**Authors:** Cheyene Almeida Celestino Menozzi, Rodolfo Rodrigo Florido França, Pedro Henrique Luccas, Mayara dos Santos Baptista, Tácio Vinício Amorim Fernandes, Lucas Villas Bôas Hoelz, Policarpo Ademar Sales Junior, Silvane Maria Fonseca Murta, Alvaro Romanha, Bárbara Verena Dias Galvão, Marcela de Oliveira Macedo, Alana da Cunha Goldstein, Carlos Fernando Araujo-Lima, Israel Felzenszwalb, Maria Cristina Nonato, Frederico Silva Castelo-Branco, Nubia Boechat

**Affiliations:** 1 Programa de Pós-Graduação em Farmacologia e Química Medicinal—PPGFQM-Instituto de Ciências Biomédicas, Universidade Federal do Rio de Janeiro, Bloco J, Ilha do Fundão, Rio de Janeiro 21941-902, Brazilrodolfo.franca@fiocruz.br (R.R.F.F.); 2 Laboratório de Síntese de Fármacos—LASFAR, Instituto de Tecnologia em Fármacos, Fundação Oswaldo Cruz, Farmanguinhos—Fiocruz, Manguinhos, Rio de Janeiro 21041-250, Brazil; 3 Laboratório de Cristalografia de Proteínas—LCP-RP, Departamento de Ciências BioMoleculares, Faculdade de Ciências Farmacêuticas de Ribeirão Preto, Universidade de São Paulo FCFRP-USP, Monte Alegre, Ribeirão Preto 14040-903, Brazil; 4 Centro de Pesquisas René Rachou/CPqRR—Fiocruz, Belo Horizonte 30190-009, Brazil; 5 Laboratório de Mutagênese Ambiental, Programa de Pós-Graduação em Biociências—PPGB—Instituto de Biologia Roberto Alcantara Gomes, Universidade do Estado do Rio de Janeiro, Rio de Janeiro 20551-030, Brazil; 6 Programa de Pós-Graduação em Biologia Molecular e Celular—PPGBMC—Instituto Biomédico, Universidade Federal do Estado do Rio de Janeiro, Rio de Janeiro 20211-010, Brazil

**Keywords:** Chagas disease, *Trypanosoma cruzi*, azole, nitroreductase, CYP51, triazole, imidazole, heterocycle

## Abstract

Chagas disease (CD), which is caused by *Trypanosoma cruzi* and was discovered more than 100 years ago, remains the leading cause of death from parasitic diseases in the Americas. As a curative treatment is only available for the acute phase of CD, the search for new therapeutic options is urgent. In this study, nitroazole and azole compounds were synthesized and underwent molecular modeling, anti-*T. cruzi* evaluations and nitroreductase enzymatic assays. The compounds were designed as possible inhibitors of ergosterol biosynthesis and/or as substrates of nitroreductase enzymes. The in vitro evaluation against *T. cruzi* clearly showed that nitrotriazole compounds are significantly more potent than nitroimidazoles and triazoles. When their carbonyls were reduced to hydroxyl groups, the compounds showed a significant increase in activity. In addition, these substances showed potential for action via nitroreductase activation, as the substances were metabolized at higher rates than benznidazole (BZN), a reference drug against CD. Among the compounds, 1-(2,4-difluorophenyl)-2-(3-nitro-1*H*-1,2,4-triazol-1-yl)ethanol (**8**) is the most potent and selective of the series, with an IC_50_ of 0.39 µM and selectivity index of 3077; compared to BZN, **8** is 4-fold more potent and 2-fold more selective. Moreover, this compound was not mutagenic at any of the concentrations evaluated, exhibited a favorable in silico ADMET profile and showed a low potential for hepatotoxicity, as evidenced by the high values of CC_50_ in HepG2 cells. Furthermore, compared to BZN, derivative **8** showed a higher rate of conversion by nitroreductase and was metabolized three times more quickly when both compounds were tested at a concentration of 50 µM. The results obtained by the enzymatic evaluation and molecular docking studies suggest that, as planned, nitroazole derivatives may utilize the nitroreductase metabolism pathway as their main mechanism of action against *Trypanosoma cruzi.* In summary, we have successfully identified and characterized new nitrotriazole analogs, demonstrating their potential as promising candidates for the development of Chagas disease drug candidates that function via nitroreductase activation, are considerably selective and show no mutagenic potential.

## 1. Introduction

Chagas disease (CD), known as American trypanosomiasis, is an infectious disease caused by the protozoan *Trypanosoma cruzi* (*T. cruzi*) and vectorized by triatomine insects [1]. This parasite has a complex cycle, and its morphophysiological characteristics are significantly altered as its life cycle phase advances. Although CD is endemic in Latin America, cases have been reported on other continents due to the migration of contaminated people, making this disease a global public health problem [2]. Currently, it is estimated that approximately 7 million people are infected worldwide and 10,000 deaths occur annually due to complications of this disease. Among parasitic diseases in the Americas, CD kills the most people [3].

The only drugs available to treat CD, benznidazole (**BZN**) and nifurtimox (NFX), were introduced to the market more than 50 years ago and cause numerous adverse effects. In addition, these drugs are ineffective in the chronic phase of the disease; thus, the complications of CD cannot be eliminated in patients [4]. These substances act as prodrugs and must undergo bioactivation to establish biological activity. The enzyme nitroreductase type I (NTR-I) is responsible for the bioactivation of these compounds through successive reduction reactions; NTR-I forms products that are toxic to the parasite and exhibit high nucleophilic character, which bind to macromolecules, such as proteins and DNA, and cause cellular damage [5]. *T. cruzi* nitroreductase (TcNTR) belongs to class I, and its mechanism of action is insensitive to oxygen. TcNTR has a flavin mononucleotide (FMN) coenzyme that is not covalently linked to its active site; thus, the reaction can proceed in two steps, following the ping-pong mechanism of catalysis. In the initial stage, an NADH molecule is oxidized, activating the catalytic site of the enzyme by reducing FMN to dihydroflavin mononucleotide (FMNH_2_). This compound can then reoxidate FMN with the concomitant sequential reduction of two electrons in nitroheterocyclic rings, enabling the generation of highly reactive nitrocomposites [6,7].

Despite the pleiomorphism of *T. cruzi*, differences in the expression levels of possible therapeutic targets greatly hinder the development of drugs that are effective against all phases of the parasite cycle. In this context, the ergosterol biosynthesis pathway, specifically its enzyme 14α-demethylase (CYP51), may be a potential target for the development of new active substances against this parasite. This is because *T. cruzi*, like fungi, contains ergosterol as the main membrane sterol, which is present in all its life stages and crucial for the fluidity of its cell membrane.

**Figure 1:**
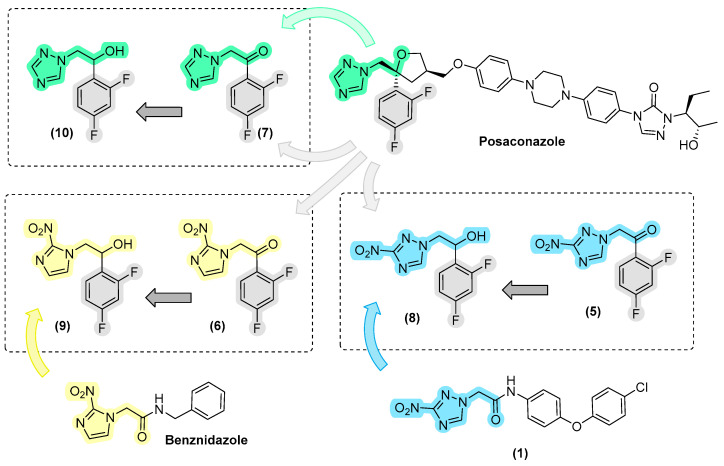
Design of azolic derivatives **5**–**10**.

Azole compounds have been described to inhibit the CYP51 enzyme by coordinating one nitrogen atom in the azole ring with an iron atom present in the heme group of the enzyme’s catalytic site. The competitive inhibition mechanism prevents the natural substrate of the enzyme from binding and blocks the biosynthesis pathway of ergosterol, causing a loss in cell membrane function and leading to the death of these microorganisms [8]. An important representative of this class is posaconazole (Figure 1), a triazole derivative originally developed as an antifungal; this compound exhibited potent in vitro activity against *T. cruzi* with an IC_50_ below 1 nM and showed cure in acute- and chronic-phase animal models, including strains resistant to **BZN** and NFX [9]. However, posaconazole was not successful in phase III clinical trials and was less effective than **BZN** [10]. Although substances that act exclusively on this target are not promising, the potency achieved by inhibiting this target could be combined with additional mechanisms of action that may lead to greater curative efficacy.

In this sense, compounds containing the 3-nitro-1,2,4-triazole heterocycle were developed and evaluated in vitro and in vivo against *T. cruzi*, showing high potency and significant selectivity indices with compound **1** (Figure 1) being a representative of this series [11,12]. Molecular modeling studies have shown that these compounds may bind to the catalytic site of the CYP51 enzyme in a similar way to ergosterol biosynthesis inhibitors, such as posaconazole. In addition, enzymatic assays have shown that these substances are significantly converted by the TcNTR enzyme. Thus, it is possible that the 3-nitro-1,2,4-triazole compounds can function through a dual mechanism, acting as substrates of NTR enzymes or as inhibitors of CYP51 [11]. When binding assays were performed with 3-nitrotriazole derivatives and *T. cruzi* CYP51 enzyme, it was observed that this target was inhibited, thus validating this approach [12].

Importantly, including the nitro group in new substances remains controversial due to its potential relationship with mutagenic and genotoxic effects [13,14]. However, these effects were demystified by our research group, as we analyzed the participation of the nitro group in the genotoxic and mutagenic effects of nitroimidazoles with anti-*T. cruzi* activity [15]. Even with NTR substrates, in which the cytotoxic effect is desired, the effects only occur after the bioactivation of the molecule.

Thus, in the search for new active substances against *T. cruzi* in the present study, we synthesized, evaluated and compared the activity of a series of derivatives containing 2-nitroimidazole, 1,2,4-triazole and 3-nitro-1,2,4-triazole. The design of these derivatives involved maintaining the 1,3-difluorbenzene ring, which is present in posaconazole and other ergosterol biosynthesis inhibitors active against *T. cruzi.* The 2-nitroimidazole heterocycle, which is present in the **BZN**, and the 3-nitro-1,2,4-triazole and 1,2,4-triazole rings, which are present in substances with potent anti-*T. cruzi* activity and in the IBEs, were added (Figure 1). In addition, the importance of the nitro group in the triazole heterocycle and the influence of the selected heterocycles on the activity against *T. cruzi* were investigated. As mentioned, combining high anti-*T. cruzi* potency characteristic of IBEs with the additional action pathway by NTR activation may increase the efficacy of these substances. In addition, different strains could be applied with different sensitivities to specific targets, minimizing the risk of therapeutic failure due to parasitic resistance.

It is important to emphasize that substances **5** and **7** have already been previously evaluated against *T. cruzi*, with the first showing moderate activity [16]. Substance **10**, although not new [17], has never been evaluated against this parasite. Finally, compounds **6**, **8** and **9** are all new, so the present work brings, in a unified way, important direct comparative aspects in relation to the anti-*T. cruzi* of these different heterocyclic systems in addition to their vicinity.

## 2. Results and Discussion

### 2.1. Organic Synthesis

**Scheme 1:**
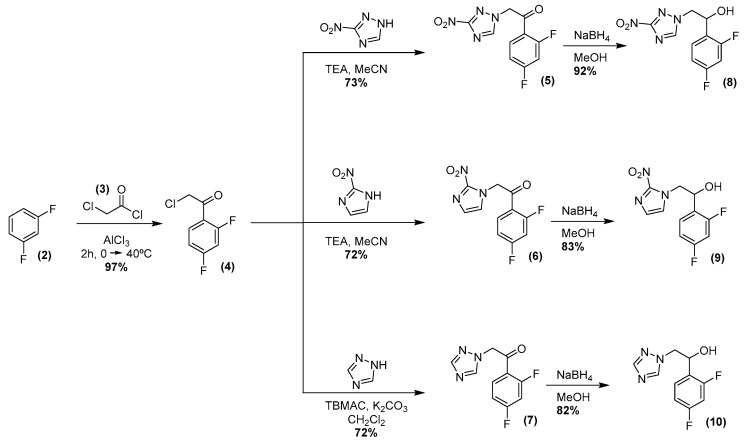
Synthesis of azolic derivatives **5**–**10**.

Derivatives **5**–**10** were obtained according to Scheme 1 by a modified method similar to that previously described by Silva et al. [16], Nelson et al. [18] and Upadhayaya et al. [19]. A simple synthetic route involving two or three stages was used. Initially, a Friedel–Craft acylation reaction was performed on 1,3-difluorbenzene (**2**) using chloroacetyl chloride (**3**) in the presence of AlCl_3_, obtaining key intermediate **4** in 97% yield. Then, key intermediate **4** reacts with the respective azoles through a nucleophilic substitution reaction at room temperature, giving rise to carbonyl derivatives **5**–**7** with yields ranging from 72 to 73%. The carbonyls of **5**–**7** derivatives were reduced through a reaction with NaBH_4_ in methanol at room temperature, generating hydroxylated derivatives **8**–**10** with yields ranging from 82 to 92% (Scheme 1).

### 2.2. Biological Evaluation

#### 2.2.1. Anti-*T. cruzi* Activity

Derivatives **5**–**10** were evaluated in vitro against *T. cruzi* in an intracellular model on the trypomastigote and amastigote forms of the parasite of the Tulahuen strain [20]. This model is interesting because it mimics the life cycle of the parasite, as both clinically relevant forms are present in the environment [21]. The mammalian host cells used were fibroblasts from L929 mice.

All derivatives showed anti-*T. cruzi* activity except for triazole derivative **7**. Among them, derivative **8** stands out, which was the most potent in the series with an IC_50_ value of 0.39 µM. In parallel, a mean cytotoxic evaluation (CC_50_) was performed on the host cell, in which all showed low cytotoxicity with CC_50_ values greater than 400 µM and thus had high selectivity indices (SI), such as that of Compound **8**, the most powerful in the series, which had an SI of 3077 (Table 1).

**Figure 2:**
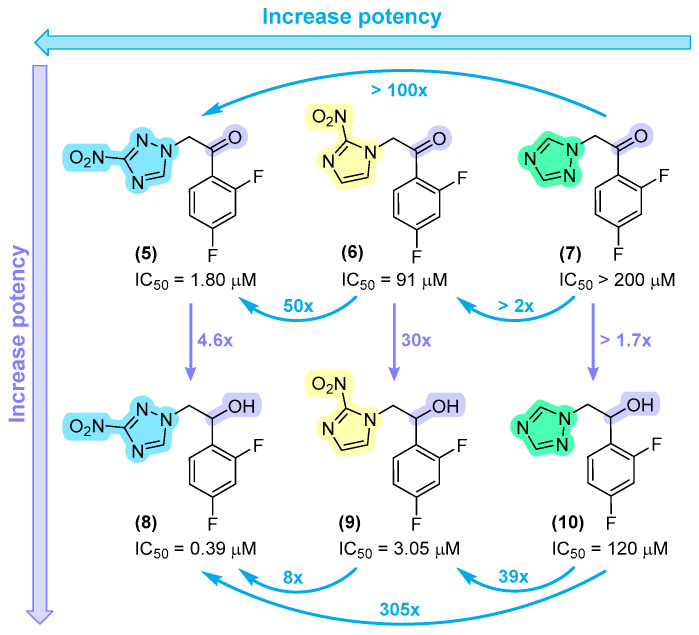
Comparison of the anti-*T. cruzi* activities among the **5**–**10** series of compounds.

The results obtained from this evaluation confirmed that the presence in the 3-nitrotriazole nucleus leads to increased activity against *T. cruzi*. Regarding the carbonyl derivatives (**5**–**7**), it is possible to observe that Compound **5** (IC_50_ *T. cruzi* = 1.80 µM) was approximately 50 times more potent than its corresponding 2-nitroimidazole **6** (IC_50_ *T. cruzi* = 90.90 µM), whereas triazole derivative **7** showed no activity (IC_50_ *T. cruzi* > 200 µM) (Figure 2). The same trend was observed for hydroxy derivatives (**8**–**10**) since nitrotriazole derivative **8** (IC_50_ *T. cruzi* = 0.39 µM) was approximately 8 times more potent than 2-nitroimidazole **9** (IC_50_ *T. cruzi* = 3.05 µM) and more than 300 times more potent than the corresponding triazole **10** (IC_50_ *T. cruzi* = 120 µM) (Figure 2). In addition, it is possible to observe that the hydroxy derivatives were more potent than their carbonyl counterparts since Compound **8** was more than 4 times more potent than **5**, derivative **9** was approximately 30 times more potent than **6** and derivative **10** showed some anti-*T. cruzi* activity, unlike **7** (Figure 2). Thus, the most potent substance in this series was hydroxy nitrotriazolic derivative **8**, which was almost 4 times more potent than **BZN** in vitro. Regarding cytotoxicity, all derivatives were slightly toxic to L929 cells. This toxicity resulted in good selectivity indices. In this regard, derivative **8** stood out, as the derivative was more than twice as selective as **BZN**. Thus, derivative **8** showed improved anti-*T. cruzi* potency and greater selectivity compared to that of this reference drug.

#### 2.2.2. *T. cruzi* Nitroreductase Enzyme Evaluation

To determine a potential mechanism of action, nitroazole derivatives **5**, **6**, **8** and **9** were evaluated as substrates of the *T. cruzi* nitroreductase enzyme (TcNTR) and compared to **BZN**, the reference prodrug (Table 2). The enzymatic activity was measured by monitoring the oxidation of NADH, the first substrate of the enzyme, via spectrophotometric assays. The results show that nitrotriazole derivatives **5** and **8** were metabolized at higher rates than **BZN** at the two concentrations evaluated (25 and 50 µM). At a concentration of 25 µM, derivatives **5** and **8** were metabolized at rates 2.3 and 2.6 times higher than that of **BZN**, respectively. At 50 µM, the compounds presented metabolism rates 1.6 and 3.0 times higher than that of **BZN**, respectively.

**Figure 3:**
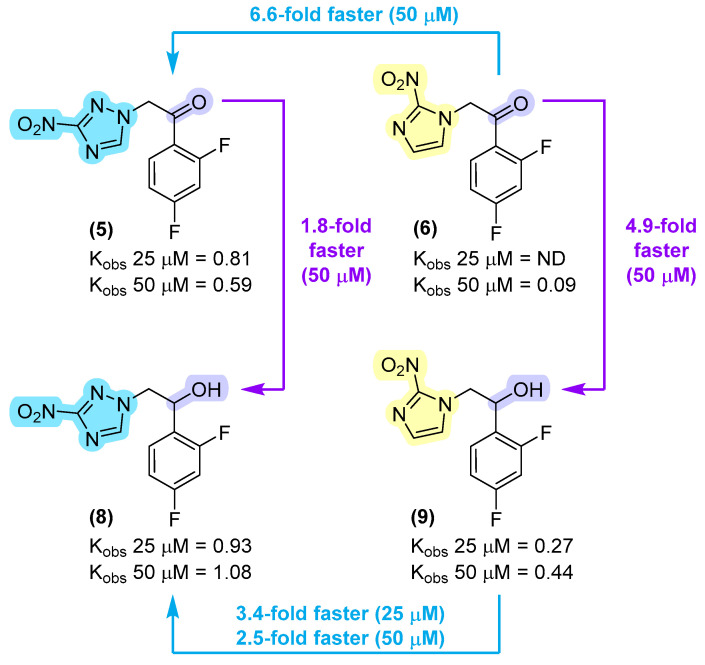
Metabolization rate of nitroazole derivatives by the enzyme TcNTR. Higher values of K_obs_ obtained indicate a higher consumption rate of this compound at a given concentration compared to the other derivatives.

Among the 2-nitroimidazole compounds, only **9** was metabolized at a concentration of 50 µM and at a higher rate than **BZN**. The nitrotriazole and nitroimidazole derivatives were compared with each other, and it was observed that the nitrotriazole derivatives were consumed at much higher speeds than the nitroimidazole at both concentrations. At a concentration of 25 µM, derivative **8** was consumed at a rate 3.4 times higher than that of derivative **9**. At a concentration of 50 µM, derivative **5** was consumed at a rate 6.6 times higher than its nitroimidazole analog **6**; derivative **8** showed a consumption rate approximately 2.5 times higher than its corresponding nitroimidazole **9**. In addition, at a concentration of 50 µM, the hydroxy derivatives exhibited a higher consumption rate than that of their ketone counterparts, regardless of the heterocycle present. In particular, nitroimidazole derivative **9** was consumed 4.9 times faster than **6**, and nitrotriazole derivative **8** exhibited a higher rate of consumption (1.8 times higher than that of **5**) (Figure 3).

Notably, in at least one concentration used in the assays, Compounds **5**, **8** and **9** showed a higher rate of consumption by the NTR than that of the reference drug. Although these isolated data do not indicate the affinity, the catalytic constant or whether the metabolites of each derivative exhibit cytotoxic effects on the parasite, the data show that the substrates are TcNTR substrates. In addition, a correlation was observed between higher K_obs_ and lower IC_50_ values, such as that observed for Compound **8**, which showed higher K_obs_ than **BZN** at both test concentrations and the lowest IC_50_ value among the molecules tested. This result suggested a significant participation of this mechanism in the inhibition of parasites induced by the compounds. The activity of Compound **6** was monitored at the highest concentration and even at a much lower rate than the others and the reference. At 25 µM, it was not possible to determine the K_obs_ values of this compound, most likely due to the low activity observed for this compound. Importantly, after reduction via TcNTR, the compounds may lose their ability to act as IBEs due to possible chemical and structural changes that may result from the reduction of the nitro group.

#### 2.2.3. In Silico ADMET Studies

An in silico prediction of several pharmacokinetic and toxicity parameters was performed by the pkCSM server [22] for Compound **8** and **BZN** (as reference drug) due to the high anti-*T. cruzi* potency and high selectivity of derivative **8** and to clarify its absorption, distribution, metabolism, excretion and toxicity (ADMET) potential, focusing on a future in vivo antichagasic evaluation (Table S1, Supplementary Data).

This prediction showed that **8** presents favorable properties, including high gastrointestinal absorption, low blood–brain barrier permeability, and classification as a nonsubstrate or inhibitor of P-glycoprotein (a macromolecule related to drug efflux) [23,24]. Furthermore, this compound has not shown potential as a substrate or inhibitor of the five cytochrome P450 (CYP) isoforms (Table S1).

Compared to **BZN** (75.83%; log BB = −0.49), Compound **8** (88.45%) shows a higher value of intestinal absorption (human) and a lower blood–brain barrier permeability (log BB = −1.89). **BZN** (0.539 mL/min/kg) was predicted to exhibit faster clearance than that of **8** (0.106 mL/min/kg) and also showed a lower putative bioavailability (Table S1).

On the other hand, the toxicity predictions show that **BZN** and **8** were classed as hepatotoxic, as well as possible mutagens in the Ames test, although the compounds were negative for hERG I and II inhibition (Table S1).

#### 2.2.4. Hepatocytotoxicity Assessment in HepG2 Cells

Regarding the in vitro liver cell toxicity obtained using HepG2 cells (Table 3), the results suggested that the safety profile for all the tested compounds was considerably safe, with low cytotoxic responses in WST-1 and LDH assays. Therefore, the compounds did not induce a considerable reduction in cell viability either by mitochondrial dysfunction or by cell membrane damage. Cytotoxicity and genotoxicity using liver cell cultures have been widely used over the last decade to detect synthetic and natural drug genetic toxicity [25] and predict liver injury [26]. Drug-induced liver toxicity is divided into fatty liver disease (steatosis), cholestasis or acute liver injury, and using a proteomic analysis, van Summeren et al. showed that HepG2 cells were an accurate model to predict drug-induced acute liver injury (by oxidative disbalance and consequently necrosis) and cholestasis (by secondary hepatocyte lesion, resulting in hepatopathy), mechanisms often associated with nitroderivative hepatotoxicity, as observed in nitroimidazoles [27].

#### 2.2.5. In Vitro Mutagenicity Assessment by Ames Test

Concerning the in vitro mutagenicity assessment of **8** and **9**, both compounds were less mutagenic **BZN**. The results obtained from the Ames Test (Table 4) demonstrate that Compound **8** was not mutagenic in the three tested *S. typhimurium* strains, either in the absence or presence of metabolic activation, even at the highest concentration (assay’s detection limit). However, without the S9 mix, cytotoxic responses were observed for the TA1535 and TA98 strains at 500 µg/plate. On the other hand, Compound **9** and **BZN** were considered mutagenic when tested in G:C to A:T pair substitution strains (TA1535 and TA100), with different levels of mutagenic potency, and were remarkably more mutagenic in the recombinational DNA repair-proficient strain (TA100). According to the Claxton et al. [28,29] classification, Compound **9** was a highly inductive mutagen in TA100 both in −S9 (250.3 revertants/µM) and in +S9 (292.1 revertants/µM). Similar to Compound **9**, **BZN** was classified as a mutagen but was more harmful than the prototypes and was an extremely high mutagen in TA100 under −S9 (1789.9 revertants/µM) and +S9 (1382.2 revertants/µM) conditions. This panel of strains was chosen for their ability to detect 93% of the bacterial mutagens detected collectively by all the TG471-recommended bacterial strains [30,31], reducing the cost, time and redundancy of the results. Furthermore, the preincubation method, which is more sensitive to nitrocompounds [32], was effective in mutagenicity detection since a higher probability of short-lived mutagenic metabolites can react with the tester strains in the small volume of preincubation mixture and consequently a higher concentration of S9 mix [33].

Mostly, nitroheterocyclic compounds act as prodrugs, which must be activated to exert their bioactivity [34]; some examples are NFX and **BZN**, which are activated by an oxygen-insensitive type I nitroreductase (NTR) expressed in trypanosomes but absent in mammalian cells. NTR typically fragments the heterocyclic ring by carrying out a series of two-electron reduction reactions, resulting in the production of toxic metabolites [5]. In 2-nitroimidazoles, the hydroxylamine metabolite may undergo rearrangement and hydration, producing a dihydro-dihydroxyimidazole, which releases glyoxal after decomposition. The latter two products interact with biomolecules, including DNA, forming adducts and thiols [35]. In agreement with what was shown in the present work, Papadopoulou et al. showed that 3-nitro-1,2,4-triazoles are also TcNTR substrates; therefore, the compounds may utilize a similar mechanism of action in terms of their interaction with parasite DNA [11].

Boechat et al. demonstrated that the nitro group is not solely responsible for nitroimidazole genotoxic activity, showing the importance of studying nitroderivatives to determine which substitutions can reduce or eliminate mutagenic effects and thus maintain the trypanocidal potential [15]. It is also important to emphasize that although mutagenicity in *S. typhimurium* strains was observed, it does not necessarily translate to mutagenicity in humans. For instance, although **BZN** mutagenicity in humans has never been clearly reported, the drug was reported to be mutagenic for TA98 and TA100, corroborating our data [36,37]. Furthermore, in a mutagenicity study with the serum and urine from guinea pigs, Ferreira et al. demonstrated that **BZN** is not metabolized by the mammalian host into stable mutagenic derivatives detectable by *S. typhimurium* strains, suggesting a reduced risk of potential carcinogenic effects in humans [38].

Although ample data are available on nitroimidazoles, studies addressing the mutagenicity of nitrotriazoles remain scarce. Papadopoulou et al. evaluated the mutagenicity of a 3-nitrotriazole-based benzylamide with excellent in vivo antichagasic activity (IC_50_ 0.113 µM) using TA98 and TA7001–7006, a set of mixed strains, each of which carries a unique missense mutation in the histidine biosynthetic operon [39,40]. The compound induced a point mutation for the mixed strains with S9 at the highest concentration (1000 µg/mL) and was toxic to L6 cells (rat skeletal myoblasts). However, in the absence of a linear dose–response factor, the authors assumed there was a safe threshold. They also observed that 2-nitroimidazole compounds were associated with mutagenic activity to a greater degree than nitrotriazoles.

## 3. Molecular Docking on the TcCYP51 Enzyme

**Figure 4:**
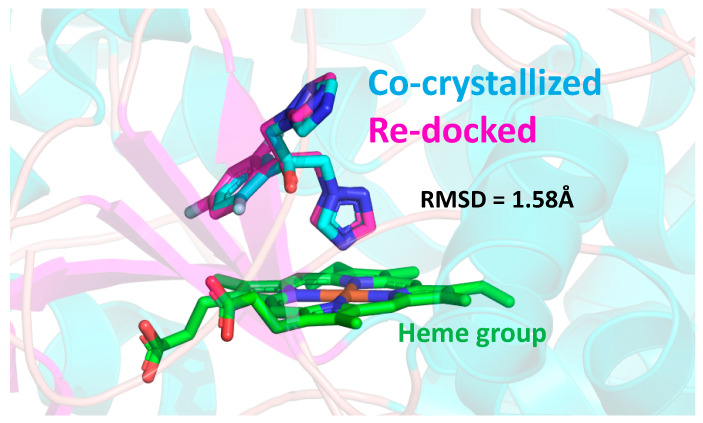
Superposition of the cocrystal fluconazole (TPF) and redocked pose. Three-dimensional (3D) representation of the TPF in the binding site of the 14α-demethylase structure (CYP51). The cyan color represents the cocrystal TPF pose (carbon atoms), and the redocked pose of TPF (carbon atoms) is preserved in magenta. Carbons of the heme group are represented in green. The other atoms were colored according to PyMOL defaults (v2.5.0, https://pymol.org, accessed on 18 April 2023) [43]. The root mean square deviation (RMSD) value between the TPF crystal coordinates and predicted coordinates is also shown.

**Figure 5:**
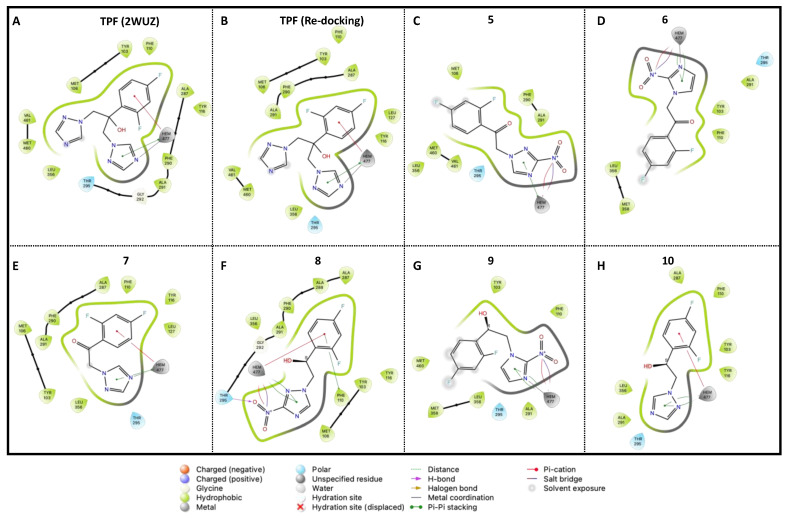
Two-dimensional (2D) diagrams of protein–ligand interactions in the active sites of the 14α-demethylase structure (CYP51) (PDBid.: 2WUZ) [41] using the Maestro (Schrödinger) program [44]. The interactions of cocrystallized (**A**) and docked (**B**) poses of fluconazole (TPF) with CYP51 residues and heme groups. 2D docking interactions of Compounds **5**–**10** poses (**C**–**H**) with 14α-demethylase residues and heme groups. Protein–ligand interactions are represented with arrows/lines between ligand atoms and protein residues: H-bonds are represented by solid pink arrows; π–π stacking interactions are shown in green lines; π–cation interactions are represented by red lines; metal coordination is shown in purple line; the salt bridge is represented in red-to-blue gradient lines. The colors in residue circles indicate the residue type: (acidic, red; basic, purple; hydrophobic, green; polar, blue; glycines, light green; metal atoms, dark gray; other, light gray). Ligand atoms that are exposed to solvent are marked with gray spheres. The protein “pocket” is displayed with a line around the ligand, colored with the color of the nearest protein residue.

**Figure 6:**
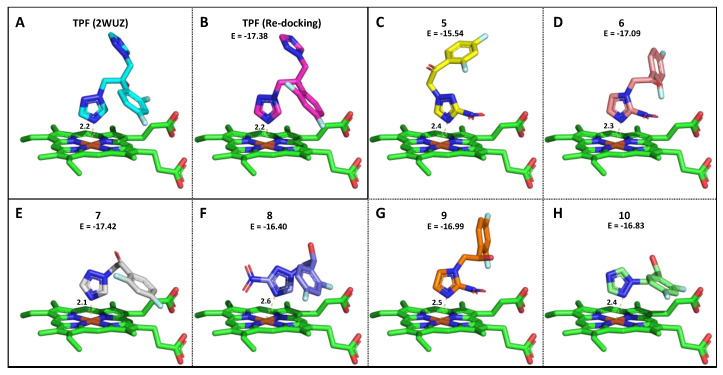
Distance (in Å) of the heme Fe^2+^ to the proximal aromatic nitrogen (N.ar) of the nitrotriazole and imidazole rings in cocrystallized (**A**) and docked (**B**) poses of fluconazole (TPF) and Compounds **5**–**10** (**C**–**H**) are shown in A through H. Furthermore, better docking energies are also shown (in kcal/mol). Heme carbons are represented in green, cocrystallized TPF carbons are shown in cyan and redocked TPF carbons are in magenta. Carbons in derivatives **5**–**10** are shown in yellow, pink, gray, purple–blue, orange, and green–cyan, respectively. The other atoms are colored according to PyMOL [43] defaults.

Considering the possible mechanism of action in the *T. cruzi* ergosterol biosynthesis pathway, which involves inhibiting the enzyme TcCYP51, a molecular docking study was carried out to evaluate the possible interaction between the derivatives and this enzyme. The binding of fluconazole (TPF) to TcCYP51 was predicted by molecular docking using PDBid: 2WUZ [41]. Docking was carried out using the GalaxyDock2-HEME [42] program. Initially, the docking protocol was validated by redocking TPF, which reproduced the same binding mode observed in the crystal structure (Figure 4), with a root mean square deviation (RMSD) value of 1.58 Å. All major interactions between TPF and TcCYP51 amino acid residues resemble those in the redocked pose (Figure 5), including the interaction between Fe^2+^ of the heme group and one of the nitrogen atoms from the ligand’s triazolyl ring. The binding energy of the redocked best pose is −17.38 kcal/mol (Table 5). The binding modes of derivatives **5**–**10** were determined through molecular docking simulations applying the validated protocol. The results showed that all compounds exhibit similar affinities to the receptor (Figure 6) (Table 5). Furthermore, the distance between heme Fe^2+^ and the proximal aromatic nitrogen (N.ar) of the nitrotriazole and imidazole rings was also similar. Even Compound **5** reached a smaller distance when compared to the TPF crystal binding mode. However, Compounds **5**, **8** and **10** did not show metal coordination. This probably contributed to their higher energies, indicating a lower energy of interaction with this enzyme. Nonetheless, the metal coordination can be recovered since molecular docking simulations capture only a snapshot of the complex interaction and not its dynamic behavior. Despite this, the complex energies were similar to those found in the redocking simulation. Compounds with NO_2_ moieties perform interactions with the heme group via electrostatic interactions, π–π stacking and π–cation. In this sense, there was no clear correlation between the interaction energies with CYP51 and activity against *T. cruzi*, which suggests that the high antiparasitic activity of Compound **8** is probably due to its activation by the nitroreductase enzyme, since this substance was metabolized more quickly by this enzyme.

## 4. Experimental Section

### 4.1. Chemistry

All reagents and solvents used were from Sigma-Aldrich^®^ (Burlington, VT, USA) and were of analytical grade. The yields presented were calculated after respective purification. Thin-layer chromatography (TLC) was performed using Merck^®^ (Darmstadt, Germany) TLC Silica gel 60 F254 aluminum sheets 20 cm × 20 cm (eluent Ehyl acetate/hexane 1:1). The melting points (m.p.) were determined using a Büchi^®^ (Flawil, Switzerland) model B-545 apparatus. The ^1^H, ^13^C and ^19^F nuclear magnetic resonance (NMR) spectra were generated at 400.00, 100.00 and 376.00 MHz, respectively, in a Bruker^®^ Avance instrument equipped with a 5 mm probe. Tetramethylsilane (TMS) was used as an internal standard, and deuterated dimethylsulfoxide (DMSO-d_6_) was used as a solvent. The chemical shifts (δ) are reported in ppm, and the coupling constants (J) are reported in Hertz. Gas chromatography with mass spectrometry detection (GC–MS) was performed using the Agilent^®^ (Santa Clara, CA, USA) Model 6890 Chromatograph with Agilent^®^ Model 5973 at 70 eV. Mass spectrometry with electrospray ionization in positive mode (ESI–MS (+)) was carried out in Waters^®^ Micromass ZQ4000 equipment (Milford, MA, USA). Values are expressed as mass/charge ratio (*m*/*z*) and are equivalent to the molecular mass of the substance plus a proton or its sodium adduct. The HRMS data were obtained using LC–MS Bruker^®^ Daltonics MicroTOF (Yokohama, Kanagawa, Japan) (analyzer time of flight). The determination of the purity of the substances was carried out by high-performance liquid chromatography (HPLC) on Shimadzu^®^ liquid chromatograph using a Supelcosil LC-8 column (Kyoto, Japan) (250 mm × 4.6 mm × 3 μm) and as mobile phase acetonitrile: potassium phosphate buffer 0.01 mol/L, pH 5.8, flow 1 mL/min.

#### 4.1.1. Synthesis of the Intermediate 2-Chloro-1-(2,4-difluorophenyl)ethanone (**4**)

This compound was obtained as described by Upadhayaya et al. [19] with modifications. In a flask, 20 g (0.17 mol) of 1,3-difluorbenzene was added and cooled to 0 °C, and then 19.65 g (1 equivalent) of chloroacetyl chloride was added. The reaction medium was stirred for 15 min at this temperature. Then, 24.59 g (1.05 equivalents) of aluminum chloride was added gradually, stirring for 1.5 h at 10 °C and at 40 °C for another 30 min. The reaction medium was poured into crushed ice (150 g) and concentrated hydrochloric acid solution. The mixture was stirred for approximately 30 min. Then, the aqueous phase was extracted once with 100 mL of ethyl ether. The organic phase was washed twice with 100 mL of saturated sodium bicarbonate solution and once with 100 mL of saline solution. The organic phase was dried with anhydrous sodium sulfate and concentrated in a rotary evaporator, generating the product of interest without the need for a subsequent purification step.

Yield: 91%; GC–MS: *m/z* 141 (100), *m/z* 113 (30), *m/z* 142 (8), *m/z* 127 (7), *m/z* 93 (2); melting point: 46–47 °C (lit. 46–48 °C [19]); ^1^H NMR (400 MHz, DMSO-d_6_) δ 8.01 (td, *J* = 8.7, 6.7 Hz, 1H), 7.48 (ddd, *J* = 11.7, 9.3, 2.5 Hz, 1H), 7.28 (dddd, *J* = 8.8, 8.0, 2.5, 0.8 Hz, 1H), 5.05 (d, *J* = 2.6 Hz, 2H). ^13^C NMR (101 MHz, DMSO-d_6_) δ 188.1 (d, *J* = 4.4 Hz), 165.5 (dd, *J* = 254.9, 12.8 Hz), 162.0 (dd, *J* = 257.7, 13.3 Hz), 132.8 (dd, *J* = 11.0, 4.0 Hz), 120.0 (dd, *J* = 13.1, 3.6 Hz), 112.6 (dd, *J* = 21.8, 3.4 Hz), 105.3 (dd, *J* = 27.7, 26.1 Hz), 50.2 (d, *J* = 9.5 Hz).

#### 4.1.2. Synthesis of 1-(2,4-Difluorophenyl)-2-(3-nitro-1*H*-1,2,4-triazol-1-yl)ethanone (**5**)

This compound was obtained as described by Silva et al. [16] with modifications. Intermediate **4**, 1.5 equivalents of 3-nitro-1*H*-1,2,4-triazole, 2 equivalents of triethylamine and acetonitrile were added to a flask. The mixture was stirred at room temperature for 3 h. The reaction medium was concentrated and dissolved in dichloromethane. Then, extraction was performed using water. Finally, the organic phase was dried with anhydrous sodium sulfate and concentrated on a rotary evaporator.

Yield: 73%; melting point: 117–119 °C (lit. 113–114 °C [16]); ESI–MS (+): 269 [M+H]^+^, 291 [M + Na]^+^, 307 [M + K]^+^; HPLC: 100% (λ = 250 nm); HRMS (+): exp.: *m/z* 291.0303/calc.: *m/z* 291.0300; GC–MS: *m/z* 141 (100), *m/z* 113 (17), *m/z* 142 (8), *m/z* 127 (2), *m/z* 114 (1); ^1^H NMR (400 MHz, DMSO-d_6_) δ 8.81 (H-14, s, 1H), 8.07 (H-6, td, *J* = 8.7, 8.7, 6.6 Hz, 1H), 7.57 (H-3, ddd, *J* = 11.7, 9.3, 2.4 Hz, 1H), 7.34 (H-5, dddd, *J* = 8.7, 7.9, 2.5, 0.7 Hz, 1H), 6.04 (H-9, d, *J* = 3.0 Hz, 2H); ^13^C NMR (101 MHz, DMSO-d_6_) δ 187.9 (C-7, d, *J* = 4.7 Hz), 165.8 (C-2, dd, *J* = 255.9, 13.0 Hz), 162.5 (C-4, dd, *J* = 258.1, 13.3 Hz), 161.9 (C-12, s), 148.3 (C-14, s), 132.6 (C-6, dd, *J* = 11.1, 4.0 Hz), 119.2 (C-1, dd, *J* = 13.0, 3.5 Hz), 112.8 (C-5, dd, *J* = 21.9, 3.3 Hz), 105.4 (C-3, dd, *J* = 27.0, 26.0), 59.3 (C-9, d, *J* = 11.8 Hz); ^19^F NMR (376 MHz, DMSO-d6) δ −100.01–−100.22 (F-2, m), −102.91–−103.11 (F-4, m).

#### 4.1.3. Synthesis of 1-(2,4-Difluorophenyl)-2-(2-nitro-1H-imidazol-1-yl)ethanone (**6**)

A total of 500 mg (0.0026 mol) of intermediate **4**, 440 mg (1.5 eq.) of 2-nitroimidazole, 525 mg (2 eq.) of triethylamine and 6.0 mL of acetonitrile were added to a flask. The reaction was stirred at room temperature for 24 h. The reaction medium was concentrated and dissolved in dichloromethane. The organic phase was extracted with water. Then, the organic phase was dried with anhydrous sodium sulfate and concentrated on a rotary evaporator. The product was recrystallized in ethanol.

Yield: 72%; HPLC: 99.85% (λ = 265 nm); HRMS (+): exp.: *m/z* 268.0532/calc.: *m/z* 268.0528; GC–MS: *m/z* 222 (5), *m/z* 221 (41), *m/z* 142 (7), *m/z* 141 (100), *m/z* 113 (20); IRν (cm^−1^)/stretch: 1240 (CF), 1690 (C = O), 1359 (NO). Measured melting point: 128–130 °C; ^1^H NMR (400 MHz, DMSO-d_6_) δ 8.05 (H-6, td, *J* = 8.7, 8.5, 6.6 Hz, 1H), 7.63 (H-14, d, *J* = 1.0 Hz, 1H), 7.59 (H-3, ddd, *J* = 11.7, 9.3, 2.4 Hz, 1H), 7.35 (H-5, td, *J* = 8.4, 8.4, 2.5 Hz, 1H), 7.28 (H-13, d, *J* = 1.0 Hz, 1H), 5.97 (H-9, d, *J* = 2.9 Hz, 2H); ^13^C NMR (100 MHz, DMSO-d_6_) δ 189.1 (C-7, d, *J* = 4.9 Hz), 166.4 (C-2, dd, *J* = 256.0, 12.9 Hz), 163.0 (C-4, dd, *J* = 257.5, 13.4 Hz), 145.4 (C-11, s), 133.2 (C-6, dd, *J* = 11.2, 4.1 Hz), 129.0 (C-14, s), 128.4 (C-13, s), 119.7 (C-1, dd, *J* = 13.3, 3.5 Hz), 113.6 (C-5, dd, *J* = 21.9, 3.2 Hz), 106.0 (C-3, dd, *J* = 27.3, 26.5 Hz), 59.0 (C-9, d, *J* = 12.2 Hz). ^19^F NMR (377 MHz, DMSO-d_6_) δ −99.90–−100.06 (F-2, m), −103.45–−103.57 (F-4, m).

#### 4.1.4. Synthesis of 1-(2,4-Difluorophenyl)-2-(1*H*-1,2,4-triazol-1-yl)ethanone (**7**)

This compound was obtained as described by Upadhayaya et al. [19] with modifications. A total of 10 g (0.05263 mol) of intermediate **4**, 10,894 g (3 eq.) of 1,2,4-triazole, 8 g (1.1 eq.) of potassium carbonate, 2.45 g of tributylmethylammonium chloride and 150 mL of dichloromethane were added to a flask. The reaction was stirred at room temperature for 24 h. At the end of the reaction, 20 mL of concentrated hydrochloric acid was added, and the reaction medium was extracted with 50 mL of water until total removal of the product from the organic phase. The aqueous phase was basified (pH = 10), and sodium chloride solution was added. The product of interest was vacuum-filtered and washed with ice water.

Yield: 72%; GC–MS: *m/z* 223, *m/z* 195 (3), *m/z* 142 (7), *m/z* 141 (100), *m/z* 127 (2), *m/z* 113 (24); melting point: 104–105 °C (lit. 104–105 °C [19]); ^1^H NMR (400 MHz, DMSO-d_6_) δ 8.49 (H-14, s, 1H), 8.04 (H-6, td, *J* = 8.6, 6.6 Hz, 1H), 8.02 (H-12, s, 1H), 7.53 (H-3, ddd, *J* = 11.7, 9.3, 2.5 Hz, 1H), 7.31 (H-5, dddd, *J* = 8.9, 8.0, 2.5, 0.7 Hz, 1H), 5.82 (H-9, H-9′, d, *J* = 3.0 Hz, 2H); ^13^C NMR (101 MHz, DMSO-d_6_) δ 189.21 (C-7, d, *J* = 5.0 Hz), 165.59 (C-2, dd, *J* = 255.3, 12.8 Hz), 162.33 (C-4, dd, *J* = 257.8, 13.5 Hz), 151.19 (C-12, s), 145.49 (C-14, s), 132.52 (C-6, dd, *J* = 11.1, 4.1 Hz), 119.62 (C-1, dd, *J* = 13.2, 3.6 Hz), 112.67 (C-5, dd, *J* = 21.7, 3.3 Hz), 105.31 (C-3, dd, *J* = 27.0, 26.0 Hz), 57.69 (C-9, d, *J* = 10.8 Hz); ^19^F NMR (377 MHz, DMSO-d_6_) δ −100.78 (F-4, dq, *J* = 15.9, 7.9 Hz), −103.34 (F-2, td, *J* = 12.2, 8.7 Hz).

#### 4.1.5. Synthesis of 1-(2,4-Difluorophenyl)-2-(3-nitro-1*H*-1,2,4-triazol-1-yl)ethanol (**8**)

A total of 200 mg (0.000746 mol) of **5** and 4.5 mL of methanol were added to a flask. After complete solubilization, 31 mg (1.1 equivalents) of sodium tetrahydroborate was added. The reaction was stirred at room temperature for 1 h. The solvent was concentrated in vacuo, and the solid obtained was dissolved in ethyl acetate and extracted with acidified water and brine solution. The organic phase was collected, dried with anhydrous sodium sulfate, filtered by gravity and evaporated in vacuo.

Yield: 92%; melting point: 128–129 °C; HPLC: 100% (λ = 250 nm); HRMS (+): exp.: *m/z* 226.0790/calc.: *m/z* 226.0786; ESI–MS (+): 293 [M + Na]^+^, 309 [M + K]^+^; GC–MS: *m/z* 143 (100), *m/z* 128 (46), *m/z* 115 (34), *m/z* 95 (18), *m/z* 141 (11); ^1^H NMR (400 MHz, DMSO-d_6_) δ 8.83 (H-14, s, 1H), 7.54 (H-6, td, *J* = 8.6, 8.6, 6.6 Hz, 1H), 7.27 (H-3, ddd, *J* = 10.8, 9.3, 2.5 Hz, 1H), 7.14 (H-5, tdd, *J* = 8.5, 8.5, 2.5, 1.0 Hz, 1H), 6.09 (H-8, d, *J* = 4.9 Hz, 1H), 5.21 (H-7, q, *J* = 5.8 Hz, 1H), 4.49 (H-9, d, *J* = 6.1 Hz, 2H); ^13^C NMR (101 MHz, DMSO-d_6_) δ 161.9 (C-12, s), 161.8 (C-2, dd, *J* = 246.3, 12.3 Hz), 159.1 (C-4, dd, *J* = 247.2, 12.5 Hz), 147.5 (C-14, s), 129.2 (C-6, dd, *J* = 10.0, 6.0 Hz), 124.4 (C-1, dd, *J* = 14.1, 3.6 Hz), 111.6 (C-5, dd, *J* = 21.2, 3.5 Hz), 103.7 (C-3, t, *J* = 26.0 Hz), 64.5 (C-7, d, *J* = 1.7 Hz), 56.1 (C-9, s). ^19^F NMR (376 MHz, DMSO-d_6_) δ −110.95 (F-2, dt, *J* = 16.0, 8.6, 7.8 Hz), −114.94 (F-4, qd, *J* = 10.5, 8.6, 1.0 Hz).

#### 4.1.6. Synthesis of 1-(2,4-Difluorophenyl)-2-(2-nitro-1*H*-imidazol-1-yl)ethanol (**9**)

A total of 150 mg (0.000562 mol) of derivative **6** and 5 mL of methanol were added to a flask. The reaction medium was stirred for 5 min. After total solubilization, 3.4 mg (1.1 eq.) of sodium tetrahydroborate was added. The reaction was stirred at room temperature for 30 min. The precipitate was filtered and washed with ice water. There was no need for subsequent purification.

Yield: 83%; HPLC: 100% (λ = 265 nm); HRMS (+): exp.: *m/z* 270.0696/calc.: *m/z* 270.0685; GC–MS: *m/z* 143 (97), *m/z* 127 (100), *m/z* 115 (50), *m/z* 97 (91), *m/z* 95 (31); IRν (cm^−1^)/stretch: 3394 (OH), 1066 (C-OH), 1271 (CF), 1356 (NO); melting point: 184–186 °C; ^1^H NMR (400 MHz, DMSO-d_6_) δ 7.49 (H-14, d, *J* = 1.0 Hz, 1H), 7.44 (H-6, td, *J* = 8.7, 8.5, 6.9 Hz, 1H), 7.21 (H-3, ddd, *J* = 10.7, 9.4, 2.6 Hz, 1H), 7.13 (H-13, d, *J* = 1.0 Hz, 1H), 7.10 (H-5, td, *J* = 9.0, 8.7, 2.6 Hz, 1H), 5.98 (H-8, d, *J* = 4.9 Hz, 1H), 5.18 (H-7, td, *J* = 7.7, 4.9, 4.3 Hz, 1H), 4.63 (H-9, H-9′, dd, *J* = 13.6, 4.3 Hz, 1H), 4.55 (H-9, H-9′, dd, *J* = 13.6, 7.7 Hz, 1H); ^13^C NMR (100 MHz, DMSO-d_6_) δ 162.3 (C-2, dd, *J* = 246.1, 12.3 Hz), 159.6 (C-4, dd, *J* = 247.3, 12.4 Hz), 145.3 (C-11, s), 129.6 (C-6, dd, *J* = 10.0, 6.1 Hz), 129.2 (C-14, s), 127.7 (C-13, s), 125.2 (C-1, dd, *J* = 14.4, 3.6 Hz), 112.1 (C-5, dd, *J* = 21.2, 3.5 Hz), 104.0 (C-3, t, *J* = 26.0 Hz), 65.0 (C-7, d, *J* = 1.4 Hz), 54.9 (C-9, s); ^19^F NMR (377 MHz, DMSO-d_6_) δ −111.12 (F-2, dt, *J* = 16.0, 8.6, 7.8 Hz), −114.97 (F-4, qd, *J* = 10.5, 8.6, 1.0 Hz).

#### 4.1.7. Synthesis of 1-(2,4-Difluorophenyl)-2-(1*H*-1,2,4-triazol-1-yl)ethanol (**10**)

This compound was obtained as described by Nelson et al. [18] with modifications. A total of 1 g (0.0045 mol) of intermediate **7** and 15 mL of methanol were added to a flask. The reaction medium was stirred for 5 min. After total solubilization, 190 mg (1.1 eq.) of sodium tetrahydroborate was added. The reaction was stirred at room temperature for 30 min. The precipitate was filtered and washed with ice water. There was no need for subsequent purification.

Yield: 82%; HPLC: 98.70% (λ = 265 nm); HRMS (+): exp.: *m/z* 226.0790/calc.: *m/z* 226.0786; GC–MS: *m/z* 143 (49), *m/z* 115 (28), *m/z* 95 (19), *m/z* 83 (100), *m/z* 82 (37); IRν (cm^−1^)/stretch: 3118 (OH), 1131 (C-OH), 1279 (CF); Melting point: 119–121 °C (lit. 118–120 °C [18]); ^1^H NMR (400 MHz, DMSO-d_6_) δ 8.41 (H-14, s, 1H), 7.93 (H-12, s, 1H), 7.52 (H-6, td, *J* = 8.5, 8.5, 7.0 Hz, 1H), 7.21 (H-3, ddd, *J* = 10.8, 9.5, 2.4 Hz, 1H), 7.10 (H-5, td, *J* = 8.5, 8.5, 2.4 Hz, 1H), 5.94 (H-8, s, 1H), 5.16 (H-7, dd, *J* = 7.2, 5.0 Hz, 1H), 4.40–4.28 (H-9, H-9′, m, 2H); ^13^C NMR (100 MHz, DMSO-d_6_) δ 162.2 (C-2, dd, *J* = 244.2, 10.7 Hz), 159.6 (C-4, dd, *J* = 245.3, 10.8 Hz), 151.7 (C-12, s), 145.2 (C-14, s), 129.7 (C-6, dd, *J* = 9.9, 6.2 Hz), 125.7 (C-1, dd, *J* = 14.2, 3.6 Hz), 112.0 (C-5, dd, *J* = 21.1, 3.5 Hz), 104.0 (C-3, t, *J* = 26.0 Hz), 65.2 (C-7, d, *J* = 1.3 Hz), 55.1 (C-9, s); ^19^F NMR (377 MHz, DMSO-d_6_) δ −111.46 (F-2, dt, *J* = 16.0, 8.6, 7.8 Hz), −115.27 (F-4, qd, *J* = 10.5, 8.6, 1.0 Hz).

### 4.2. Biological Evaluation

#### 4.2.1. In Vitro Anti-*T. cruzi* Evaluation Protocol in an Intracellular Model against the Tulahuen Strain

This assay was performed as previously described by Buckner et al. (1996) [45] using *T. cruzi* (Tulahuen strain), which expresses the β-galactosidase gene of *Escherichia coli*. Infectious trypomastigote forms were obtained by culturing fibroblast monolayers of L929 mice in RPMI-1640 medium (pH 7.2–7.4) without phenol red (Gibco BRL), containing 10% fetal bovine serum and 2 mM glutamine. For the bioassay, 4000 L929 cells in 80 µL of supplemented medium were added to each well of a 96-well microtiter plate. After overnight incubation, 40,000 trypomastigotes in 20 µL were added to the cells and incubated for 2 h. The medium containing parasites that did not penetrate the cells was replaced by 200 µL of fresh medium, and the plate was incubated for another 48 h to establish the infection. The medium was then replaced with compound solutions at 1.0 µg/mL in fresh medium (200 µL), and the plate was incubated for 96 h at 37 °C. After this period, 50 µL of chlorophenol D-galactopyranoside red (500 µM) in 0.5% Nonidet P40 was added to each well, and the plate was incubated for 18 h at 37 °C. After this, the absorbance was measured at 570 nm. Controls with uninfected cells and infected cells treated with benznidazole were performed in parallel. The results are expressed as the percentage of *T. cruzi* growth inhibition in cells tested with compost compared to infected and untreated cells. The tests were performed in triplicate.

#### 4.2.2. Protocol Used to Evaluate the In Vitro Cytotoxicity of L929 Cells

For this bioassay, 4000 mammalian cells in 200 µL of RPMI-1640 medium (pH 7.2–7.4) (Gibco BRL) plus 10% fetal bovine serum and 2 mM glutamine were added to each well of a 96-well microtiter plate, which was incubated for three days at 37 °C. The medium was then replaced with solutions of the compounds (diluted in 200 µL of supplemented medium without phenol red) at concentrations 50 times above the IC_50_ found in the anti-*T. cruzi* activity assay, and the plate was incubated for four days at 37 °C. After this period, 20 µL of AlamarBlue^TM^ was added to each well, and the plate was incubated for another 4–6 h. Then, the absorbance was measured at 570 nm [45]. Controls with cells not treated and treated with benznidazole were performed in parallel, and triplicates were performed on the same plate. The results are expressed as the percent difference in the reduction between treated (CT) and untreated cells (UT) using the following equation:

(1) $$\frac{\left(117.216\right)\left(Abs570\;TC\right)-\left(80.586\right)(Abs600\;TC)}{\left(117.216\right)\left(Abs570\;UT\right)-\left(80.586\right)(Abs600\;UT)}\;\times \;100$$

#### 4.2.3. Kinetic Assay with *Trypanosoma cruzi* Nitroreductase Enzyme (TcNTR)

The protocols for expressing, purifying and conducting kinetic analysis with the recombinant TcNTR enzyme were adapted from the methods originally developed by Shane Wilkinson, with certain modifications [46]. For this study, we employed a longer and more stable construct, encompassing residues 72 to 312 [5].

For the kinetics assays, it was prepared a 200 μL solution which included 50 mM Tris-Base at pH 7.5, with a constant concentration of both NADH (50 μM) and the substances **5**, **6**, **8**, **9** or BZN. Specifically, two distinct fixed concentrations (25 and 50 μM) of the substances **5**, **6**, **8**, **9** or BZN were tested. These prepared mixtures were then incubated for 5 min at room temperature. Subsequently, the reaction was initiated by adding NTR to achieve a final concentration of 10 μg/mL. The reaction rates were monitored using a Hitachi U-2900 spectrophotometer, measuring the consumption of the primary substrate, NADH (λ = 340 nm; ε = 6.220 M^−1^ cm^−1^), over a 2-min interval. The resulting data curves were analyzed through linear regression using OriginPro 2016 software. To evaluate the reaction rate (Kobs) obtained from the 25 and 50 μM concentrations of each substrate, comparisons were made with the reference values for BZN. Measurement errors were calculated using the formula: Error = Z × σ/√n, where ‘Z’ denotes the critical value, ‘σ’ represents the standard deviation and ‘n’ is the number of test measurements, which were performed at least in triplicate.

#### 4.2.4. In Vitro Cytotoxicity Evaluation Protocol in HepG2 Cells

The Lactate Desidrogenase (LDH) Cytotoxicity Detection Kit^PLUS^ and the Cell Proliferation Reagent WST-1 assays were performed according to the manufacturer’s protocols [47,48]. The human hepatocellular carcinoma cell line HepG2 was cultured in T25/175 cm^2^ flasks with Dulbecco’s modified Eagle’s medium (DMEM, Gibco BRL) supplemented with 10% fetal bovine serum, 3.7 g/L sodium bicarbonate and 100 µg/mL penicillin–streptomycin under standard conditions (37 ± 1 °C, 95% relative humidity and 5% CO_2_). Cells were split using trypsin-EDTA (0.05%) with phenol red solution (Gibco BRL) and seeded in a 96-well flat-bottomed plate at a density of 1 × 10^4^ cells/well. After overnight incubation, the cells were washed with 1X PBS, and the medium was replaced with different compound dilutions (0–5000 µM) in fresh medium (100 µL) followed by 24 h of incubation. DMSO 1% was used as the negative control, and Triton X-100 4% was used as the positive control. For the LDH assay, after treatment, 50 µL of supernatant from each well was transferred to a 96-well flat-bottomed plate, and 50 µL of freshly prepared reaction mixture was added to each well. After 10 min of incubation in the dark at room temperature, 25 µL of stop solution was added to each well, and the absorbance was measured at 492 nm on a microplate reader (Polaris, Celer, MG, Brazil). For the WST-1 assay, after exposure, the supernatant was replaced by 100 µL of WST-1 2% in fresh medium and incubated for 3 h in the dark under standard conditions. The absorbance was measured at 440 nm. The lethal concentration for 50% (LC_50_) was calculated by nonlinear regression of log dose versus normalized response (agonist behavior). For cytotoxicity assays, significant differences between the groups were analyzed by one-way ANOVA and Tukey’s post hoc tests (*p* < 0.01).

#### 4.2.5. Mutagenicity Evaluation Protocol by the Salmonella Reverse Mutation Assay

The compounds were tested in a reverse mutation assay preincubation protocol in the absence and presence of metabolic activation following standard protocols previously proposed by Maron and Ames [49]. The histidine-dependent (*his*^−^) strains TA98 and TA100 of *Salmonella typhimurium* were inoculated in 10 mL of Oxoid nutrient broth No. 2 containing 25 µg/mL ampicillin and incubated overnight (15–18 h) at 37 ± 1 °C on an orbital shaker (CT-712, Cientec, Belo Horizonte, MG, Brazil) at 150 rpm, obtaining a concentration of 1–2 × 10^9^ cells/mL, confirmed by titration in nutrient agar plates. Briefly, 100 µL of stationary growth cultures of each strain were preincubated for 20 min on an orbital shaker (150 rpm) at 37 ± 1 °C, with 500 µL of 0.2 M phosphate buffer pH 7.4, or 500 µL of S9 mix 4% (Molecular Toxicology, Baltimore, MD, USA), and 100 µL of compound dilutions (0–5000 µM) or controls. DMSO 1% was used as a negative control. Positive controls without metabolic activation (−S9) were 4-nitroquinoline 1-oxide (4-NQO, 0.5 µg/plate) for TA98 and sodium azide (SA, 5 µg/plate) for TA100; with metabolic activation (+S9), 2-aminoanthracene (2-AA, 1 µg/plate) was used for both strains. After incubation, 2 mL of top agar (0.7% agar, 0.5% NaCl, 0.05 mM L-histidine and D-biotin) at 45 ± 3 °C was added and gently mixed. The final mixture was poured on glucose minimal agar plates (1.5% agar, 10 g/L MgSO_4_·7H_2_O; 100 g/L of C_6_H_8_O_7_·H_2_O; 500 g/L K_2_HPO_4_; 175 g/L Na(NH_4_)HPO_4_·4H_2_O, 2% glucose) and incubated for 72 h at 37 ± 1 °C. The results were expressed as the mean of revertant colonies (*His*^+^) per plate, standard deviation (SD) and mutagenicity induction fold (IF), calculated as the ratio between the number of colonies in the test group and the number of colonies in the negative control group [50]. Mutagenic potency was calculated as the slope of the linear portion of the concentration–response function and expressed as revertants/µM. All assays were performed in triplicate and repeated at least two times. Statistical analyses were carried out in GraphPad Prism software (version 5.00, GraphPad Software, Boston, MA, USA). Samples were considered mutagenic when a dose-dependent increase in the number of revertants was observed, IF > 2, and statistical analysis by one-way ANOVA followed by Dunnet’s post hoc test showed *p* < 0.01.

### 4.3. Molecular Modeling and Docking Simulation

#### 4.3.1. Cytochrome P450 14α-demethylase (CYP51) and Derivatives **5**–**10** Structure Preparation

The CYP51:Fluconazole complex structure (PDBid. 2 WUZ) [41] from *Trypanosoma cruzi* (*T. cruzi*) was selected for molecular docking simulation. The cognate ligand fluconazole (TPF) and other ligands were removed from CYP51 (14α-demethylase structure), except for the heme molecule. The 14α-demethylase structure was first prepared by the PDB2PQR web server [51] using the AMBER force field with the PROPKA 3.0 program [52] set at pH = 7.4.

The chemical structures of the compounds were drawn in ChemDraw [53], and their structures were optimized using the RESP/ESP charge derive server [54] (https://upjv.q4md-forcefieldtools.org/REDServer-Development (accessed on 4 April 2023)).

#### 4.3.2. Docking Simulation

The protein–ligand docking program GalaxyDock2-HEME [42] was used to predict the binding poses and energies of derivatives **5**–**10** to the 14α-demethylase protein. Default parameters with a docking box of 22.5 × 22.5 × 22.5 Å^3^ were used for all simulations. The coordinates of the center grid box were centered around the Fe^2+^ of the heme group. Predicted binding poses were estimated using the GalaxyDock-HEME score, a GalaxyDock BP2 score with a metal–ligand coordination bond energy term [42].

## 5. Conclusions

In summary, six azole derivatives (**5**–**10**) were synthesized in this study, and five of the derivatives showed anti-*T. cruzi* activity on the trypomastigote and amastigote forms of the parasite. The results obtained in the anti-*T. cruzi* activity test clearly demonstrate the superior potency of the derivatives containing the 3-nitro-1,2,4-triazole heterocycle compared to their corresponding 2-nitroimidazoles and 1,2,4-triazoles. This higher potency of nitrotriazole derivatives may result from their faster activation by the nitroreductase enzyme. In addition, it was observed that the nitro group more greatly influences the anti-*T. cruzi* activity of these derivatives, since the derivatives with nitrated heterocycles showed greater potency than the derivatives with heterocycles without nitro groups. Another relevant aspect is that nitrotriazole derivatives showed potential as artificial substrates of the TcNTR enzyme, as the derivatives were consumed at higher rates than BZN. The results obtained from the molecular docking studies suggest that CYP51 is not a potential target for derivative **8**, which possibly acts as a substrate of the TcNTR enzyme. Among them, derivative **8** was the most potent in terms of anti-*T. cruzi* activity with an IC_50_ of 0.39 µM and selectivity index of 3077; compared to BZN, derivative **8** was 4-fold more potent and 2-fold more selective in addition to having a favorable in silico ADMET profile. Moreover, this derivative showed a higher consumption rate in the evaluation of the TcNTR enzyme. Furthermore, this substance was not mutagenic at any concentration evaluated, whether with or without external metabolism; in addition, the substance exhibited a low potential for hepatotoxicity, as evidenced by the high CC_50_ values in HepG2 cells. Studies on an animal model of *T. cruzi* infection may confirm the potential of this new substance, which may also be a good prototype for future structural modifications in the search for a new chemotherapeutic agent against CD. Finally, this discovery helps us contribute to the next generation of anti-Chagas disease medicines, promoting the fight against this public health disease.

## Figures and Tables

**Table 1:** Anti-*T. cruzi* activity and cytotoxicity of derivatives **5**–**10** in L929 cells.

Compound	IC_50_ *T. cruzi* (µM)	CC_50_ L929 (µM)	SI
**5**	1.80 ± 0.07	1200.00 ± 0.01	667
**6**	91 ± 4	629 ± 61	7
**7**	>200	NT	NC
**8**	0.39 ± 0.01	1200.00 ± 0.01	3077
**9**	3.05 ± 0.07	>400	>131
**10**	120 ± 90	>3200	>27
**BZN**	1.5 ± 0.4	2113 ± 270	1409

NT = not tested; NC = not calculated.

**Table 2:** Evaluation of enzymatic activity in the TcNTR enzyme with a fixed concentration of NADH at 50 µM. Kobs in µmol of oxidized NADH/min/mg TcNTR. Ratio Kobs Deriv./Kobs **BZN** considered the mean values of Kobs obtained for each representative.

Derivative	25 µM Derivative + 50 µM NADH		K_obs_ Deriv./K_obs_ BZN 25 µM	50 µM Derivative + 50 µM NADH		K_obs_ Deriv./K_obs_ BZN 50 µM
	K_obs_	Error		K_obs_	Error	
**5**	0.81	0.02	2.3	0.59	0.06	1.6
**6**	ND	ND	ND	0.09	0.01	0.3
**8**	0.93	0.01	2.6	1.08	0.08	3.0
**9**	0.27	0.04	0.8	0.44	0.08	1.2
**BZN**	0.36	0.02	NA	0.36	0.03	NA

ND: not determinated; NA: not applicable.

**Table 3:** Hepatocytotoxicity of Anti-*Trypanosoma cruzi* compounds against HepG2 cells.

	LC_50_ (µM)					
	24 h		48 h		72 h	
	WST-1	LDH	WST-1	LDH	WST-1	LDH
**8**	>500	>500	426 ± 66	>500	377 ± 30	449 ± 72
**9**	>500	>500	>500	>500	>500	>500
**BZN**	>500	>500	>500	>500	>500	>500

WST-1: water-soluble tetrazolium; LDH: lactate dehydrogenase. Lethal concentration of 50% of the cell population (LC_50_) values were calculated by nonlinear regression fit of dose–response (agonist behavior) curves.

**Table 4:** Mean values ± SDs (IF) of revertant *His+* colonies of *Salmonella enterica* serovar Typhimurium strains used in the *Salmonella*/microsome assay after incubation with the compounds.

Entry	µM	−S9			+S9		
		TA1535	TA98	TA100	TA1535	TA98	TA100
**8**	0	18 ± 4 (1.0)	27 ± 3 (1.0)	71 ± 3 (1.0)	11 ± 2 (1.0)	24 ± 4 (1.0)	106 ± 3 (1.0)
	0.5	21 ± 2 (1.0)	23 ± 4 (0.9)	77 ± 5 (1.1)	11 ± 1 (1.0)	24 ± 2 (1.0)	129 ± 21 (1.2)
	5	26 ± 3 (1.0)	23 ± 1 (0.9)	77 ± 15 (1.1)	12 ± 4 (1.1)	24 ± 7 (1.0)	128 ± 6 (1.2)
	50	23 ± 6 (1.0)	30 ± 3 (1.1)	77 ± 13 (1.1)	14 ± 4 (1.3)	23 ± 3 (1.0)	150 ± 14 (1.4)
	500	**Cytotoxic**	**Cytotoxic**	91 ± 8 (1.3)	15 ± 5 (1.4)	24 ± 5 (1.0)	145 ± 8 (1.4)
	5000	–	–	108 ± 10 (1.5)	15 ± 3 (1.4)	26 ± 1 (1.1)	183 ± 9 (1.7)
**9**	0	8 ± 1 (1.0)	27 ± 3 (1.0)	71 ± 3 (1.0)	11 ± 2 (1.0)	24 ± 4 (1.0)	106 ± 3 (1.0)
	0.5	14 ± 1 (1.8)	18 ± 6 (0.7)	73 ± 5 (1.0)	14 ± 2 (1.3)	18 ± 2 (0.8)	159 ± 7 (1.5)
	5	14 ± 4 (1.8)	18 ± 2 (0.7)	88 ± 7 (1.2)	**23 ± 1 (2.1)**	18 ± 2 (0.8)	**218 ± 14 (2.1)**
	50	**22 ± 1 (2.8)**	29 ± 3 (1.1)	**164 ± 16 (2.3)**	**24 ± 1 (2.2)**	22 ± 8 (0.9)	**233 ± 48 (2.2)**
	500	**16 ± 3 (2.0)**	49 ± 5 (1.8)	**347 ± 24 (4.9)**	**Cytotoxic**	24 ± 5 (1.0)	**1025 ± 16 (9.7)**
	5000	**Cytotoxic**	**173 ± 21 (6.5)**	**1195 ± 160 (16.7)**	–	28 ± 2 (1.2)	**1216 ± 113 (11.5)**
**BZN**	0	7 ± 1 (1.0)	27 ± 3 (1.0)	137 ± 10 (1.0)	11 ± 2 (1.0)	24 ± 4 (1.0)	106 ± 3 (1.0)
	0.5	11 ± 2 (1.5)	28 ± 6 (1.1)	130 ± 6 (1.0)	13 ± 1 (1.3)	21 ± 2 (0.9)	95 ± 3 (0.9)
	5	11 ± 5 (1.5)	28 ± 4 (1.1)	217 ± 13 (1.6)	17 ± 3 (1.6)	17 ± 2 (0.9)	110 ± 8 (1.0)
	50	11 ± 1 (1.5)	28 ± 2 (1.1)	**852 ± 40 (6.2)**	**22 ± 3 (2.1)**	18 ± 1 (0.7)	**269 ± 13 (2.5)**
	500	**18 ± 4 (2.5)**	37 ± 5 (1.4)	**3848 ± 11 (28.2)**	**31 ± 2 (2.9)**	31 ± 6 (1.3)	**907 ± 10 (8.6)**
	5000	**36 ± 5 (4.9)**	**67 ± 4 (2.5)**	**7264 ± 62 (53.0)**	**32 ± 4 (3.0)**	33 ± 4 (1.4)	**6608 ± (113)**

SD: standard deviation; IF: induction factor; −S9: absence of metabolic activation; +S9: presence of metabolic activation. Bold denotes IF > 2 and *p* > 0.05 in one-way ANOVA and Dunnet’s post hoc test; Positive controls without S9: 4NQO (0.5 µg/plate) for TA98 226 ± 2 revertants; AS (5 µg/plate) for TA1535 142 ± 16 revertants and TA100 339 ± 14 revertants. With S9: 2AA (2 µg/plate) for TA98 61 ± 5 revertants, for TA1535 57 ± 5 revertants and for TA100 427 ± 12 revertants.

**Table 5:** Docking scores of the compounds in the ligand binding pocket of 14α-demethylase (CYP51) (PDBid.: 2WUZ).

Compounds	Distance (Å)	Energy (Galaxydock2-Heme Score)
**TPF** (**PDB Coordinates**)	2.2	
**TPF** (**Redocking Coordinates**)	2.2	−17.38
**5**	2.4	−15.54
**6**	2.3	−17.09
**7**	2.1	−17.42
**8**	2.6	−16.40
**9**	2.5	−16.99
**10**	2.4	−16.83

## Data Availability

Data are contained within the article and Supplementary Materials.

## Supplementary Materials

The following supporting information can be downloaded at: https://www.mdpi.com/article/10.3390/molecules28227461/s1. Table S1. ADMET properties of 8 and BZN.
