# Supplementary material for: Anti-Trypanosoma cruzi Activity, Mutagenicity, Hepatocytotoxicity and Nitroreductase Enzyme Evaluation of 3-Nitrotriazole, 2-Nitroimidazole and Triazole Derivatives
Source: Molecules. 2023 Nov 7;28(22):7461. doi: 10.3390/molecules28227461 (PMC10672842; doi:10.3390/molecules28227461)

SUPPLEMENTARY DATA

Anti-*Trypanosoma cruzi* activity, mutagenicity, hepatocytotoxicity and nitroreductase enzyme evaluation of 3-nitrotriazole, 2-nitroimidazole and triazole derivatives

Cheyene Almeida Celestino Menozzi,[a,b] Rodolfo Rodrigo Florido França,[a,b] Pedro Henrique Luccas,[c] Mayara dos Santos Baptista,[b] Tácio Vinício Amorim,[b] Lucas Villas Bôas Hoelz,[b] Policarpo Ademar Sales Junior,[d] Silvane Maria Fonseca Murta,[d] Alvaro Romanha,[d†] Bárbara Verena Dias Galvão,[e] Marcela de Oliveira Macedo[f], Alana da Cunha Goldstein,[e] Carlos Fernando Araujo-Lima,[e,f] Israel Felzenszwalb,[e] Maria Cristina Nonato,[c*] Frederico Silva Castelo-Branco,[b] and Nubia Boechat,[b*].

[a] Programa de Pós-Graduação em Farmacologia e Química Medicinal – PPGFQM- Instituto de Ciências Biomédicas, Universidade Federal do Rio de Janeiro, Bloco J, Ilha do Fundão, 21941-902, Rio de Janeiro – RJ, Brazil; [b] Laboratório de Síntese de Fármacos – LASFAR, Instituto de Tecnologia em Fármacos, Fundação Oswaldo Cruz, Farmanguinhos – Fiocruz, Manguinhos, 21041-250 Rio de Janeiro – RJ, Brazil. [c] Laboratório de Cristalografia de Proteínas – LCP-RP, Departamento de Ciências BioMoleculares, Faculdade de Ciências Farmacêuticas de Ribeirão Preto, Universidade de São Paulo FCFRP- USP, Monte Alegre, 14040-903 – Ribeirão Preto, SP – Brazil [d]Centro de Pesquisas René Rachou/CPqRR – Fiocruz, Belo Horizonte 30190-009 – MG, Brazil. [e] Laboratório de Mutagênese Ambiental, Programa de Pós-Graduação em Biociências – PPGB – Instituto de Biologia Roberto Alcantara Gomes, Universidade do Estado do Rio de Janeiro, 20551-030, Rio de Janeiro – RJ, Brazil. [f] Programa de Pós-Graduação em Biologia Molecular e Celular – PPGBMC – Instituto Biomédico, Universidade Federal do Estado do Rio de Janeiro, 20211-010 Rio de Janeiro – RJ, Brazil † in memoriam

Table S1. ADMET properties of 8 and BZN.

SMILES	8	BZN
MOL_WEIGHT	270.195	260.253
LOGP	11.981	11.077
#ROTATABLE_BONDS	4	5
#ACCEPTORS	6	5
#DONORS	1	1
SURFACE_AREA	104.824	108.586
Water solubility	-3.296	-2.782
Caco2 permeability	-0.053	0.542
Intestinal absorption (human)	88.452	75.834
Skin Permeability	-2.742	-2.768
P-glycoprotein substrate	No	Yes
P-glycoprotein I inhibitor	No	No
P-glycoprotein II inhibitor	No	No
VDss (human)	-0.931	-0.364
Fraction unbound (human)	0.331	0.299
BBB permeability	-1.189	-0.49
CNS permeability	-2.833	-2.731
CYP2D6 substrate	No	No
CYP3A4 substrate	No	No
CYP1A2 inhibitior	No	No
CYP2C19 inhibitior	No	No
CYP2C9 inhibitior	No	No
CYP2D6 inhibitior	No	No
CYP3A4 inhibitior	No	No
Total Clearance	0.106	0.539
Renal OCT2 substrate	No	No
AMES toxicity	Yes	Yes
Max. tolerated dose (human)	0.815	0.733
hERG I inhibitor	No	No
hERG II inhibitor	No	No
Oral Rat Acute Toxicity (LD50)	1.565	2.251
Oral Rat Chronic Toxicity (LOAEL)	1.012	1.594
Hepatotoxicity	Yes	Yes
Skin Sensitisation	No	No
T. Pyriformis toxicity	0.427	0.285
Minnow toxicity	1.251	0.803

1-(2,4-difluorophenyl)-2-(3-nitro-1*H*-1,2,4-triazol-1-yl)ethanone (5)

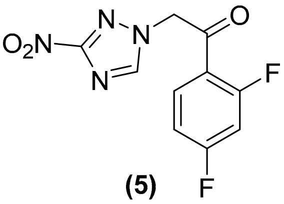

(5)

Compound Spectrum SmartFormula Report

Analysis Info

Analysis Name	D:\Data\2017\SINTESE 1\INFUSAO\108350 - PFCT 001-17.d	Acquisition Date	10/17/2017 1:56:57 PM	
Method	Tune_pos_Standard.m	Operator	Alexandre	
Sample Name	108350 - PFCT 001-17	Instrument	compact	8255754.10035
Comment				

Acquisition Parameter

Source Type	ESI	Ion Polarity	Positive	Set Nebulizer	0.4 Bar
Focus	Not active	Set Capillary	4000 V	Set Dry Heater	200 °C
Scan Begin	80 m/z	Set End Plate Offset	-400 V	Set Dry Gas	6.0 l/min
Scan End	1000 m/z	Set Charging Voltage	2000 V	Set Divert Valve	Source
		Set Corona	0 nA	Set APCI Heater	0 °C

+MS, 0.1-0.9min #3-53

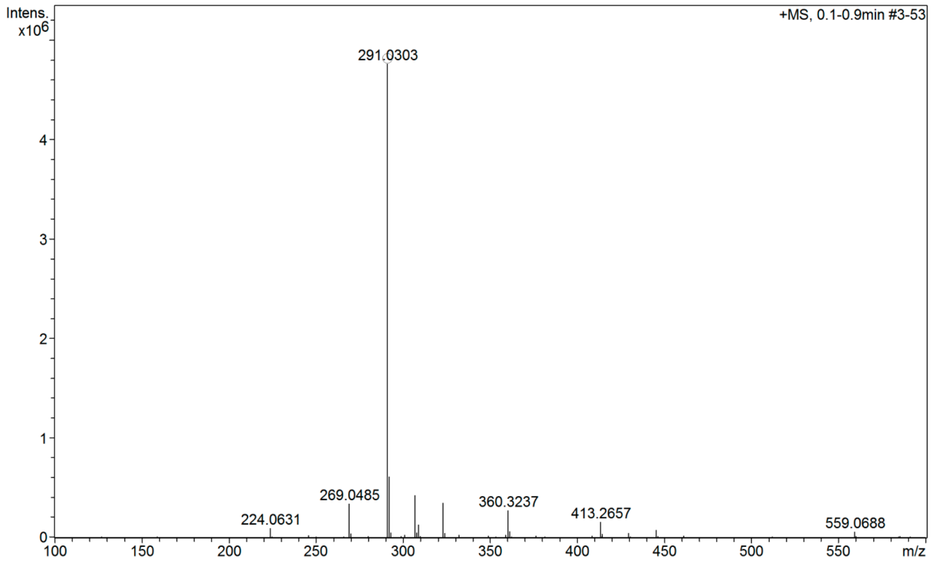

Meas. m/z	#	Ion Formula	m/z	err [ppm]	Mean err [ppm]	rdb	N-Rule	e⁻ Conf	mSigma	Std I	Std Mean m/z	Std I VarNorm	Std m/z Diff	Std Comb Dev
291.030271	1	C10H6F2N4NaO3	291.030017	-0.9	0.1	8.5	ok	even	2.8	4.3	n.a.	n.a.	n.a.	n.a.

Unknown Spectrum based on Apex

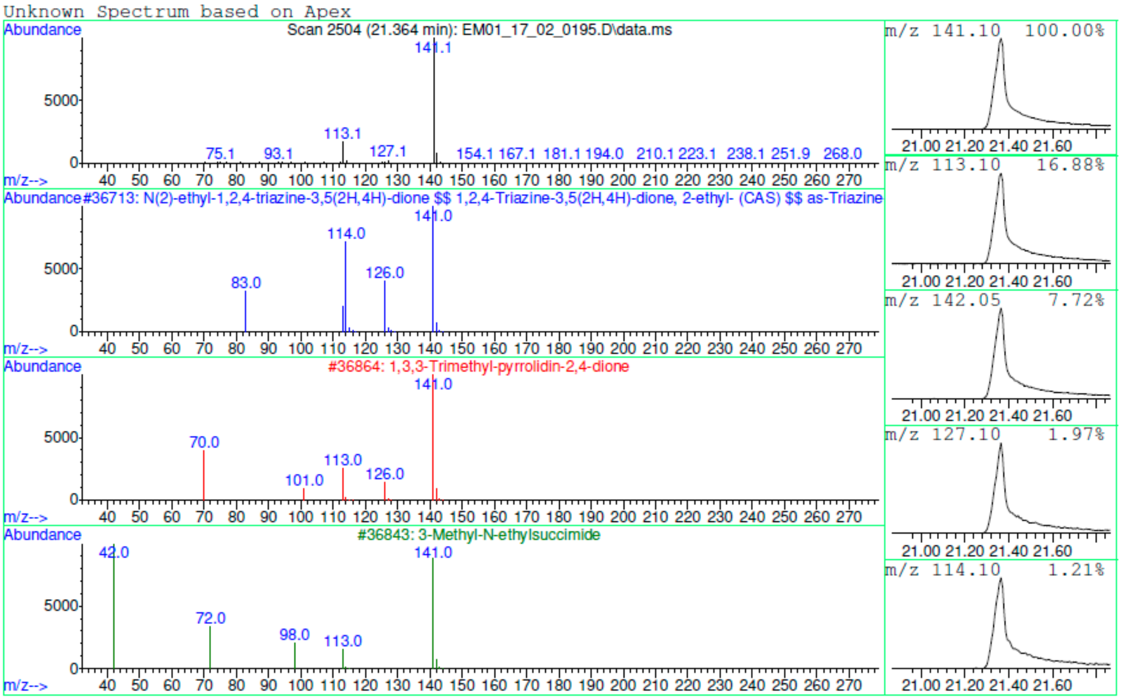

Data File: C:\Users\FFAR-21014\Documents\BACKUP DOS SISTEMAS\CG-EM-01\2017\02_FEVEREIRO_17\EM01_
... 17_02_0195.D
Sample : PFCT 001-17

Peak Number: 1 at 21.364 min Area: 200261525 Area % 100.00

The 3 best hits from each library.	Ref\#	CAS\#	Qual
C:\Database\wiley7n.l			
1 N(2)-ethyl-1,2,4-triazine-3,5(2H...	36713	089179-85-1	72
2 1,3,3-Trimethyl-pyrrolidin-2,4-d...	36864	000000-00-0	72
3 3-Methyl-N-ethylsuccimide	36843	065168-02-7	56

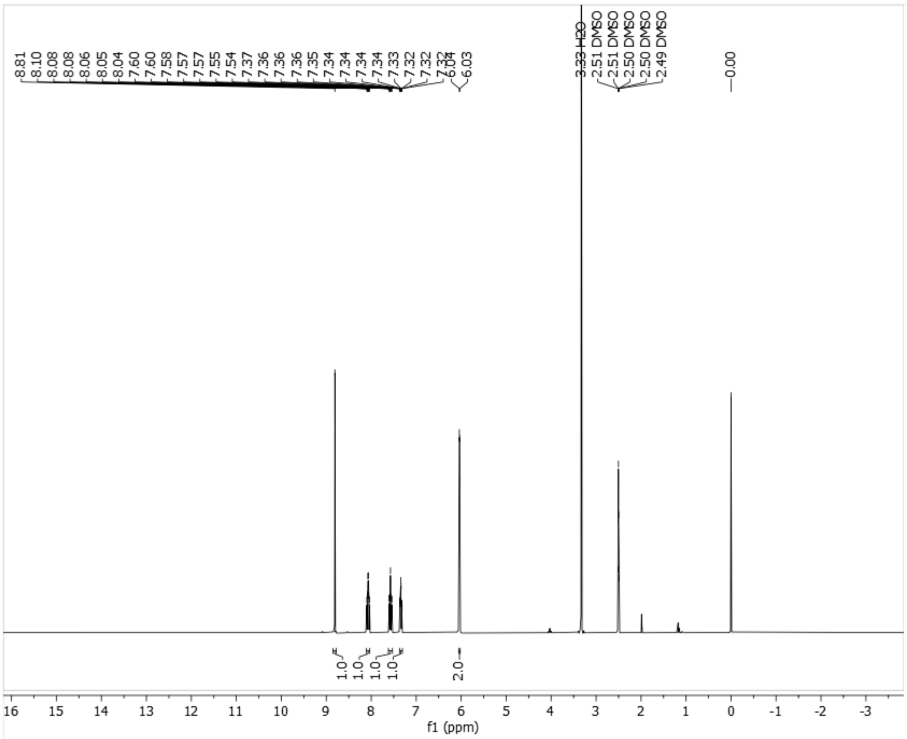

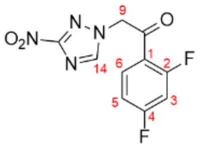

Parameter		Value
1	Origin	Bruker BioSpin GmbH
2	Owner	nmr
3	Instrument	spect
4	Solvent	DMSO
5	Temperature	298.0
6	Pulse Sequence	zg30
7	Experiment	1D
8	Probe	5 mm CPPBBO BB-1H/ 19F/ D Z-GRD Z122623/ 0041
9	Number of Scans	32
10	Receiver Gain	185.5
11	Relaxation Delay	1.0000
12	Pulse Width	11.5000
13	Acquisition Time	2.0447
14	Acquisition Date	2017-02-03T16:53:00
15	Modification Date	2018-07-16T18:32:37
16	Spectrometer Frequency	400.15
17	Spectral Width	8012.8
18	Lowest Frequency	-1537.2
19	Nucleus	1H
20	Acquired Size	16384
21	Spectral Size	65536
22	Digital Resolution	0.12

^{1}H NMR (400 MHz, DMSO-d_6) δ 8.81 (s, 0H), 8.07 (td, J = 8.7, 6.6 Hz, 1H), 7.57 (ddd, J = 11.7, 9.3, 2.4 Hz, 1H), 7.34 (dddd, J = 8.7, 7.9, 2.5, 0.7 Hz, 1H), 6.04 (d, J = 3.0 Hz, 1H).

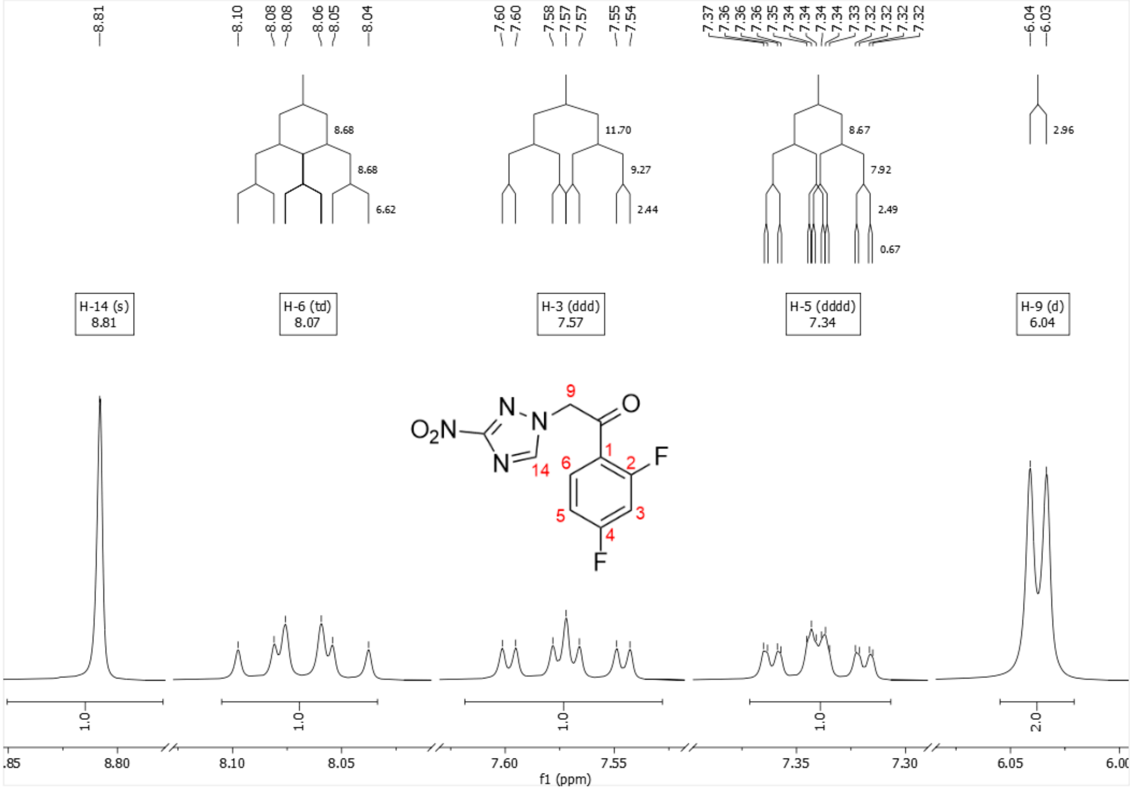

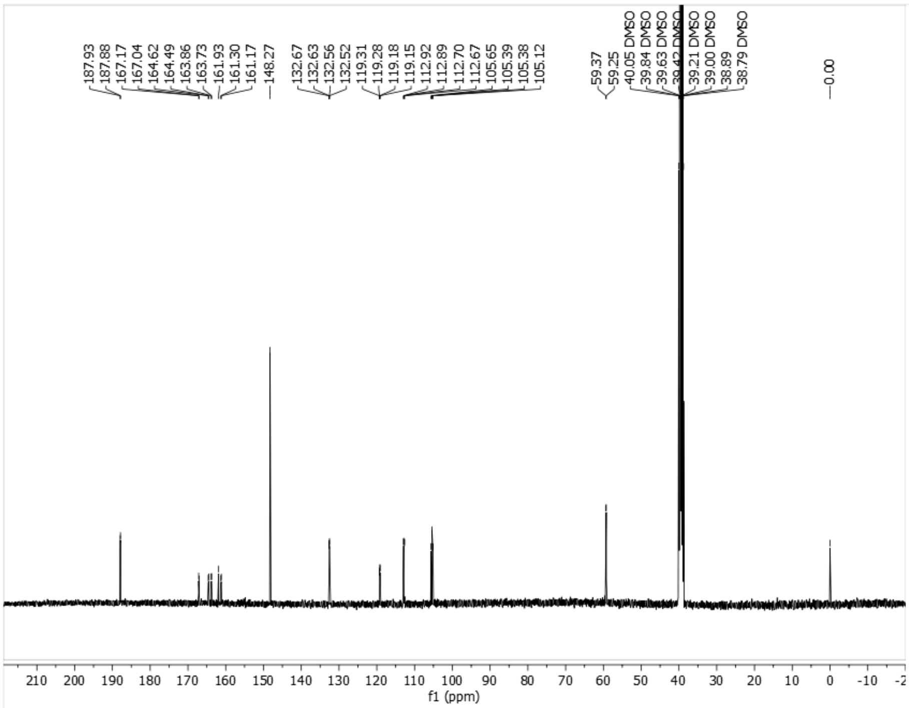

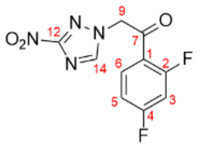

Parameter		Value
1	Origin	Bruker BioSpin GmbH
2	Owner	nmr
3	Instrument	spect
4	Solvent	DMSO
5	Temperature	298.0
6	Pulse Sequence	zgpg30
7	Experiment	1D
8	Probe	5 mm CPPBBO BB-1H/ 19F/ D Z-GRD Z122623/ 0041
9	Number of Scans	2048
10	Receiver Gain	66.7
11	Relaxation Delay	2.0000
12	Pulse Width	10.0000
13	Acquisition Time	0.6816
14	Acquisition Date	2017-02-03T18:28:00
15	Modification Date	2018-07-21T14:49:50
16	Spectrometer Frequency	100.63
17	Spectral Width	24038.5
18	Lowest Frequency	-2015.5
19	Nucleus	13C
20	Acquired Size	16384
21	Spectral Size	65536
22	Digital Resolution	0.37

^{13}C NMR (101 MHz, DMSO-d_6) δ 187.90 (d, J = 4.7 Hz), 165.83 (dd, J = 255.9, 13.0 Hz), 162.52 (dd, J = 258.1, 13.3 Hz), 161.93, 148.27, 132.59 (dd, J = 11.1, 4.0 Hz), 119.23 (dd, J = 13.0, 3.5 Hz), 112.79 (dd, J = 21.9, 3.3 Hz), 105.38 (t, J = 27.0, 26.0 Hz), 59.31 (d, J = 11.8 Hz).

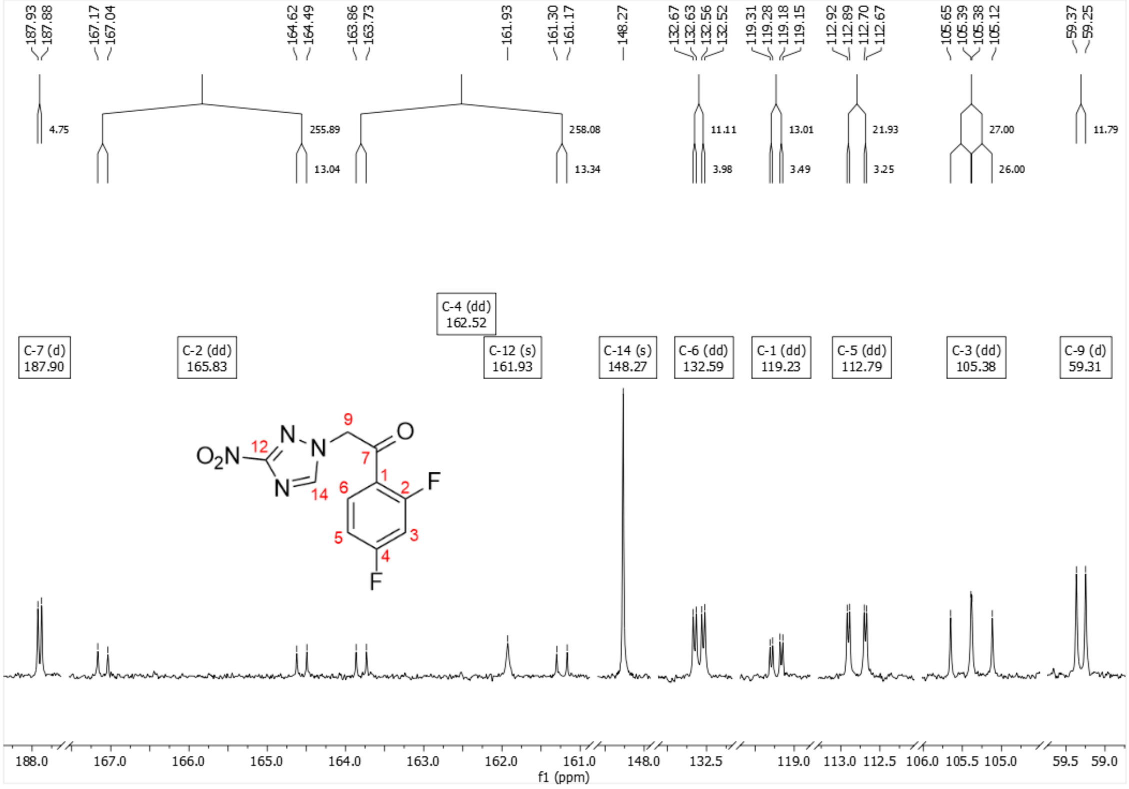

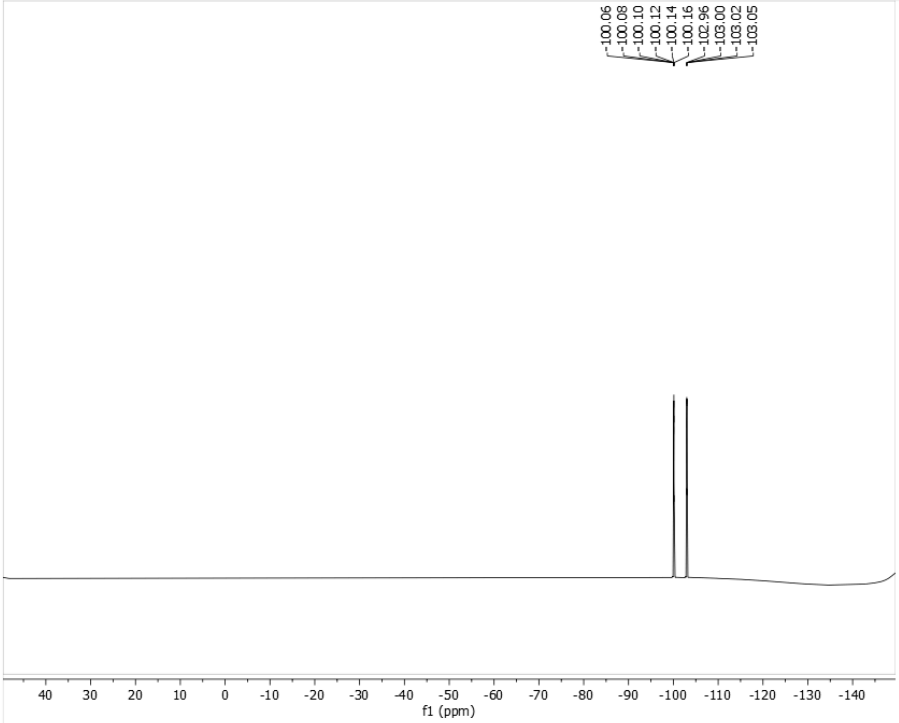

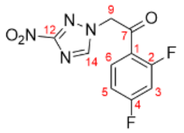

	Parameter	Value
1	Origin	Bruker BioSpin GmbH
2	Owner	nmrsu
3	Instrument	spect
4	Solvent	DMSO
5	Temperature	298.0
6	Pulse Sequence	zg30
7	Experiment	1D
8	Probe	Z122623_0041 (CPP BBO 400S1 BB-H&F-D-05 Z)
9	Number of Scans	64
10	Receiver Gain	25.4
11	Relaxation Delay	1.0000
12	Pulse Width	15.0000
13	Acquisition Time	0.8738
14	Acquisition Date	2021-04-16T04:28:46
15	Modification Date	2021-04-16T04:28:48
16	Spectrometer Frequency	376.60
17	Spectral Width	75000.0
18	Lowest Frequency	-56331.0
19	Nucleus	19F
20	Acquired Size	65536
21	Spectral Size	131072
22	Digital Resolution	0.57

^{19}F NMR (377 MHz, DMSO-d_6) δ -100.01 – -100.22 (m), -102.91 – -103.11 (m).

SERVIÇO DE MÉTODOS ANALÍTICOS

SAMPLE DESCRIPTION: PFCT 001-17
INJECTION VOLUME: 20 **SEQUENCE:** C:\CLASS-VP\Sequence\2017\Síntese\Frederico Branco\CLAE 02 - 20171113.seq **VIAL:** 2
METHOD: C:\CLASS-VP\Methods\2017\Sintese 1\Frederico Branco\Difluorofeniltriazol 1.met
FILE NAME: C:\CLASS-VP\Data\2017\Síntese\Frederico Branco\11-17\PFCT 001-17 rep2-Rep1
SAMPLE ID: PFCT 001-17 rep2 **ACQUIRED:** 13/11/2017 20:55:20 **PRINTED:** 14/11/2017 10:53:19
ANALISTA: Leandro/Thiago

Sistema de Cromatografia a Líquido: Shimadzu com 2 bombas LC-10AD, degaseificador DGU-12A, injetor automático SIL-10AD, forno de colunas CTO-10A, detector por arranjo de diodos SPD-M10A. Os dados foram adquiridos por interface SCL-10A. Aquisição de dados e controle pelo software Shimadzu CLASS-VP versão 6.13 SP2.

Coluna: Hypersil BDS-C18 150x4,6mm, 5µm - (CL0021)
Pré-Coluna: Supelguard C18 - 2cm

Fase Móvel
Eluente (A): Tampão Acetato de amônio 5mM pH5,0
Eluente (B): Acetonitrila

Programação:

Time	Module	Command	Value
0,01	Pumps	Pump B	Conc. 10
25,00	Pumps	Pump B	Conc. 90
30,00	Pumps	Pump B	Conc. 90
35,00	Pumps	Pump B	Conc. 10
40,00	Pumps	Pump B	Conc. 10
40,01	Controller	Stop	

Temperatura do forno: 25ºC **Vazão:** 1,0 mL/min

Preparo da amostra:
A 1mg de amostra, foram adicionados 1000µL de acetonitrila, seguindo de banho ultrassônico por 5 min. Uma alíquota de 400µL de solução foi transferida para um balão volumétrico de 2mL e avolumado com metanol(concentração: 200µg/mL-1). Posteriormente a solução foi filtrada em membrana PVDF 0,22µm e injetada.

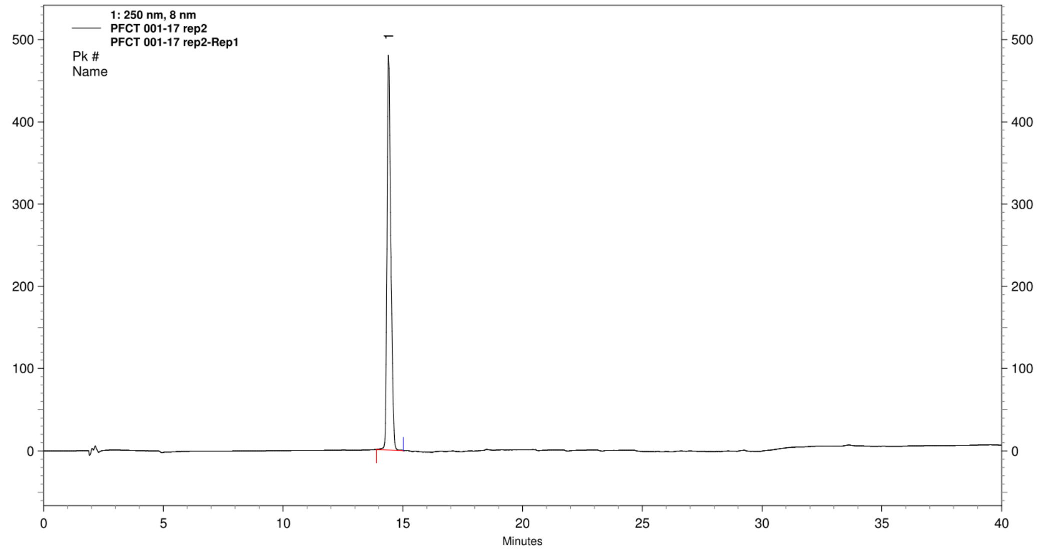

1: 250 nm, 8 nm

Pk #	Retention Time	Area	Area %	Height	Height %	Width
1	14.400	5333085	100.000	479725	100.000	1.13
Totals		5333085	100.000	479725	100.000	

SERVIÇO DE MÉTODOS ANALÍTICOS

SAMPLE DESCRIPTION: PFCT 001-17
INJECTION VOLUME: 20 **SEQUENCE**: C:\CLASS-VP\Sequence\2017\Síntese\Frederico Branco\CLAE 02 - 20171113.seq **VIAL:** 2
METHOD: C:\CLASS-VP\Methods\2017\Sintese 1\Frederico Branco\Difluorofeniltriazol 1.met
FILE NAME: C:\CLASS-VP\Data\2017\Síntese\Frederico Branco\11-17\PFCT 001-17 rep2-Rep1
SAMPLE ID: PFCT 001-17 rep2 **ACQUIRED:** 13/11/2017 20:55:20 **PRINTED:** 14/11/2017 10:53:19
ANALISTA: Leandro/Thiago

Spectrum at time 14.40 min.

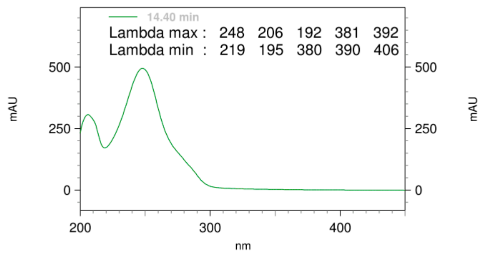

1-(2,4-difluorophenyl)-2-(2-nitro-1*H*-imidazol-1-yl)ethanone (6)

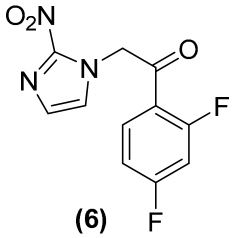

(6)

Compound Spectrum SmartFormula Report

Analysis Info

Analysis Name	D:\Data\2016\SINTESE 1\INFUSAO\80857 - PRCH 043-15.d	Acquisition Date	3/3/2016 8:44:28 AM	
Method	Tune_pos_Standard.m	Operator	BDAL@DE	
Sample Name	80857 - PRCH 043-15	Instrument	compact	8255754.10035
Comment				

Acquisition Parameter

Source Type	ESI	Ion Polarity	Positive	Set Nebulizer	0.4 Bar
Focus	Not active	Set Capillary	4500 V	Set Dry Heater	200 °C
Scan Begin	50 m/z	Set End Plate Offset	-300 V	Set Dry Gas	4.0 l/min
Scan End	1500 m/z	Set Charging Voltage	2000 V	Set Divert Valve	Source
		Set Corona	0 nA	Set APCI Heater	0 °C

+MS, 0.0-0.5min #2-30

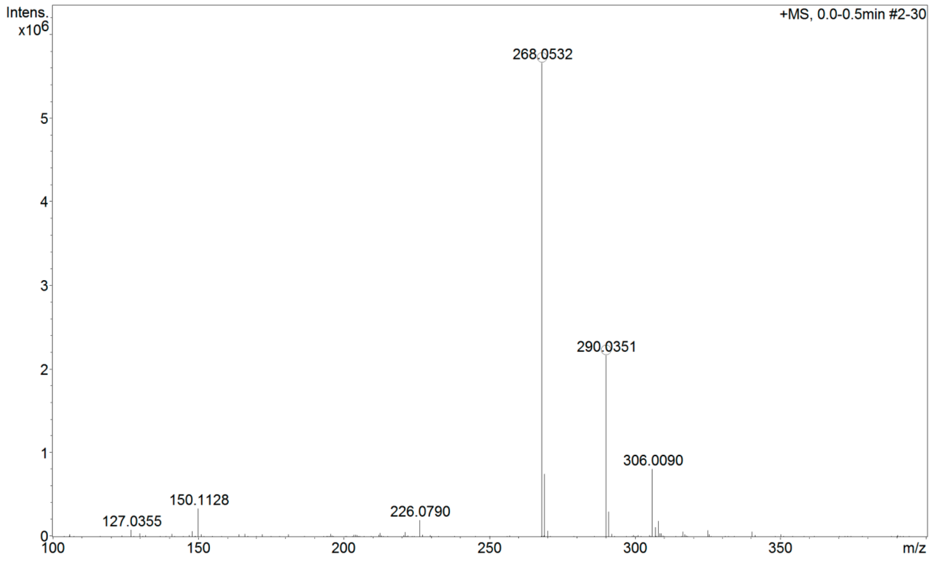

Meas. m/z	#	Ion Formula	m/z	err [ppm]	Mean err [ppm]	rdb	N-Rule	e⁻ Conf	mSigma	Std I	Std Mean m/z	Std I VarNorm	Std m/z Diff	Std Comb Dev
268.053176	1	C11H8F2N3O3	268.052824	-1.3	-1.1	8.5	ok	even	1.1	2.3	n.a.	n.a.	n.a.	n.a.
	2	C7H4F2N9O	268.050139	-11.3	-13.7	9.5	ok	even	13.2	27.6	n.a.	n.a.	n.a.	n.a.
	3	C16H8F2NO	268.056847	13.7	15.2	12.5	ok	even	26.8	50.4	n.a.	n.a.	n.a.	n.a.
290.035105	1	C11H7F2N3NaO3	290.034768	-1.2	-1.6	8.5	ok	even	3.0	5.3	n.a.	n.a.	n.a.	n.a.
	2	C7H3F2N9NaO	290.032083	-10.4	-13.4	9.5	ok	even	16.4	34.1	n.a.	n.a.	n.a.	n.a.
	3	C16H7F2NNaO	290.038791	12.7	13.4	12.5	ok	even	23.7	43.9	n.a.	n.a.	n.a.	n.a.

Unknown Spectrum based on Apex

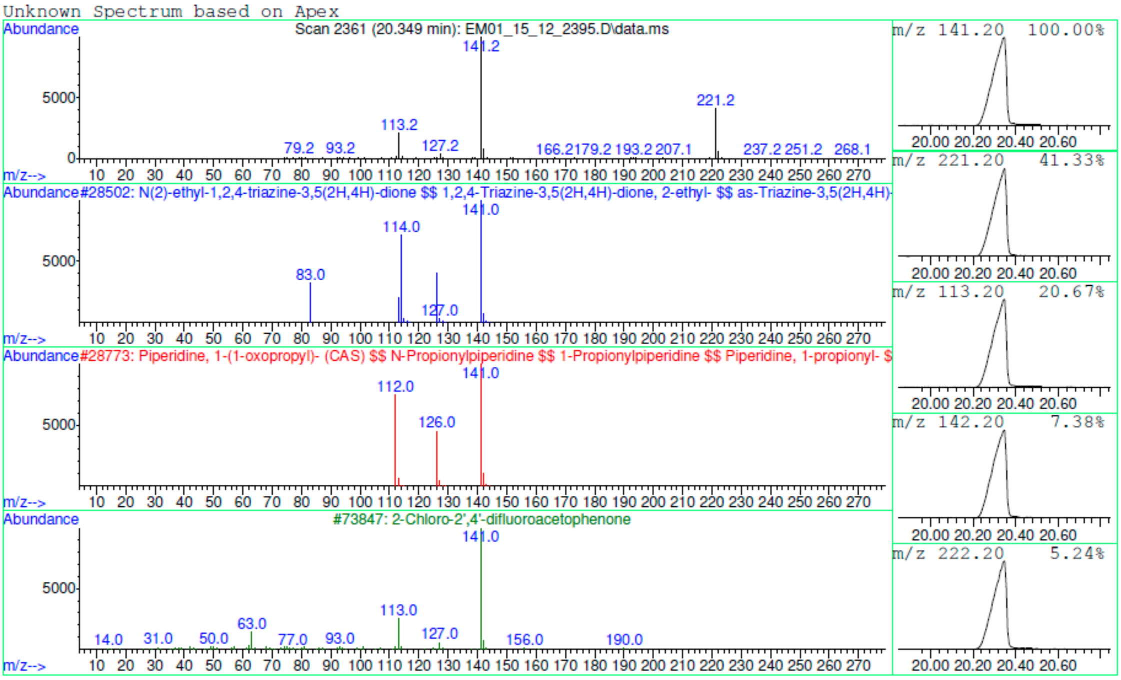

Data File: D:\Backup dos Sistemas\CG-EM-01\2015\12_DEZEMBRO_15\EM01_15_12_2395.D
Sample : PRCH 043-15

Peak Number: 1 at 20.349 min Area: 310107825 Area % 100.00

The 3 best hits from each library.	Ref\#	CAS\#	Qual
C:\Database\WILEY275.L			
1 N(2)-ethyl-1,2,4-triazine-3,5(2H...	28502	089179-85-1	50
2 Piperidine, 1-(1-oxopropyl)- (CA...	28773	014045-28-4	37
3 2-Chloro-2',4'-difluoroacetophenone	73847	051336-94-8	36

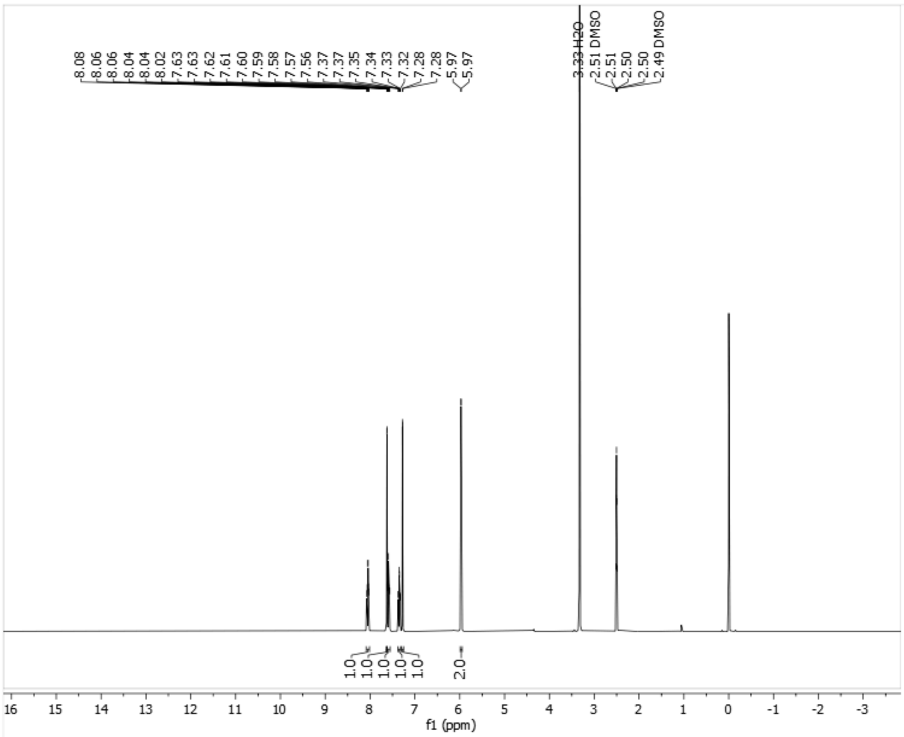

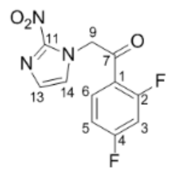

	Parameter	Value
1	Origin	Bruker BioSpin GmbH
2	Owner	nmrsu
3	Instrument	spect
4	Solvent	DMSO
5	Temperature	298.0
6	Pulse Sequence	zg30
7	Experiment	1D
8	Probe	5 mm CPPBBO BB-1H/ 19F/ D Z-GRD Z122623/ 0041
9	Number of Scans	64
10	Receiver Gain	163.2
11	Relaxation Delay	1.0000
12	Pulse Width	11.5000
13	Acquisition Time	4.0894
14	Acquisition Date	2015-12-17T11:36:00
15	Modification Date	2015-12-17T12:38:26
16	Spectrometer Frequency	400.15
17	Spectral Width	8012.8
18	Lowest Frequency	-1537.5
19	Nucleus	1H
20	Acquired Size	32768
21	Spectral Size	65536
22	Digital Resolution	0.12

^{1}H NMR (400 MHz, DMSO-d_6) δ 8.05 (td, J = 8.7, 6.6 Hz, 1H), 7.63 (d, J = 1.0 Hz, 1H), 7.59 (ddd, J = 11.7, 9.3, 2.4 Hz, 1H), 7.35 (td, J = 8.4, 2.3 Hz, 2H), 7.28 (d, J = 1.0 Hz, 1H), 5.97 (d, J = 2.9 Hz, 3H).

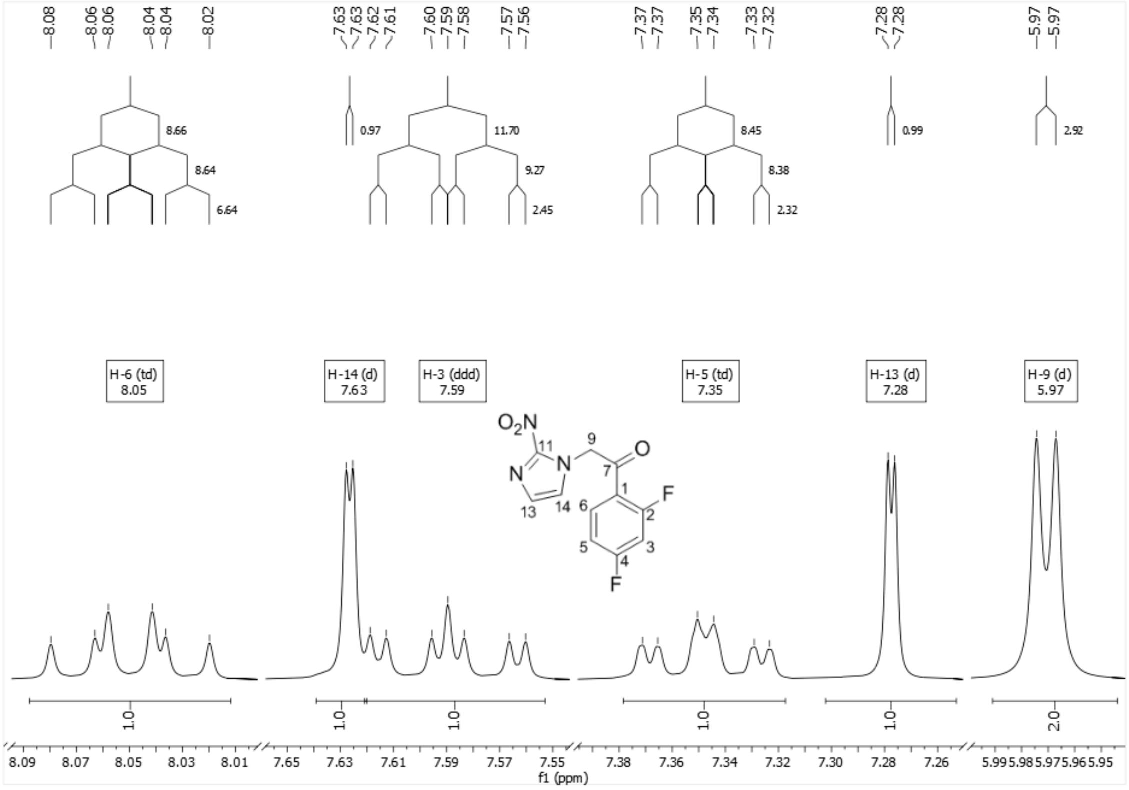

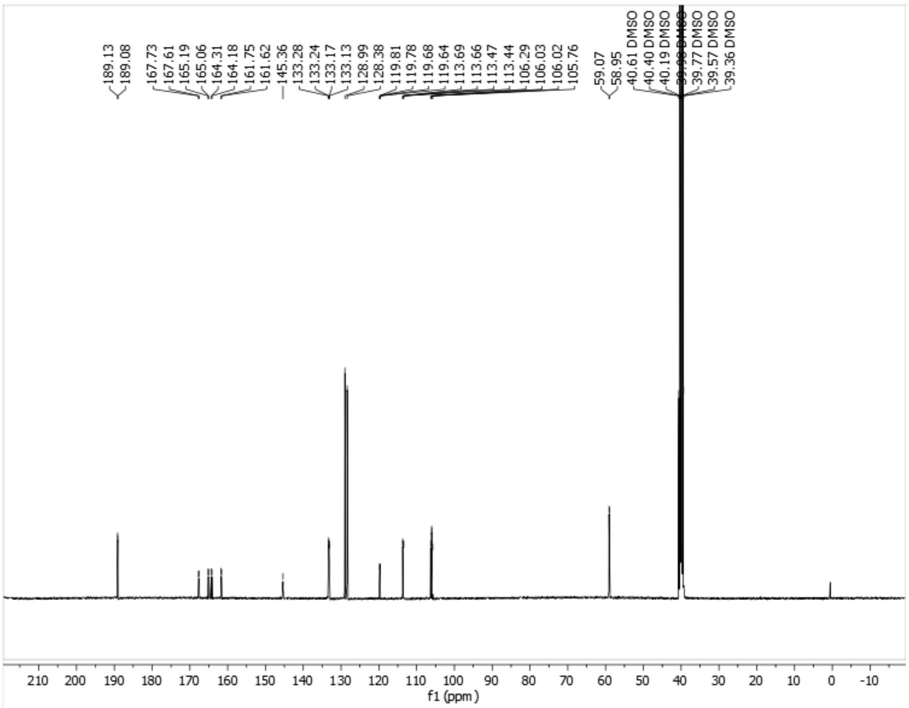

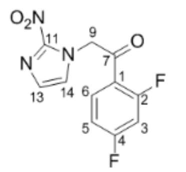

	Parameter	Value
1	Origin	Bruker BioSpin GmbH
2	Owner	nmr
3	Instrument	spect
4	Solvent	DMSO
5	Temperature	298.0
6	Pulse Sequence	zgpg30
7	Experiment	1D
8	Probe	5 mm CPPBBO BB-1H/ 19F/ D Z-GRD Z122623/ 0041
9	Number of Scans	4096
10	Receiver Gain	66.7
11	Relaxation Delay	1.0000
12	Pulse Width	10.0000
13	Acquisition Time	1.3631
14	Acquisition Date	2016-03-03T07:29:00
15	Modification Date	2016-03-03T07:38:16
16	Spectrometer Frequency	100.63
17	Spectral Width	24038.5
18	Lowest Frequency	-1957.5
19	Nucleus	13C
20	Acquired Size	32768
21	Spectral Size	65536
22	Digital Resolution	0.37

^{13}C NMR (101 MHz, DMSO-d_6) δ 189.10 (d, J = 4.9 Hz), 166.40 (dd, J = 256.0, 12.9 Hz), 162.96 (dd, J = 257.5, 13.4 Hz), 145.36, 133.20 (dd, J = 11.2, 4.1 Hz), 128.99, 128.38, 119.73 (dd, J = 13.3, 3.5 Hz), 113.57 (dd, J = 21.9, 3.2 Hz), 106.42 – 105.53 (m), 59.01 (d, J = 12.2 Hz).

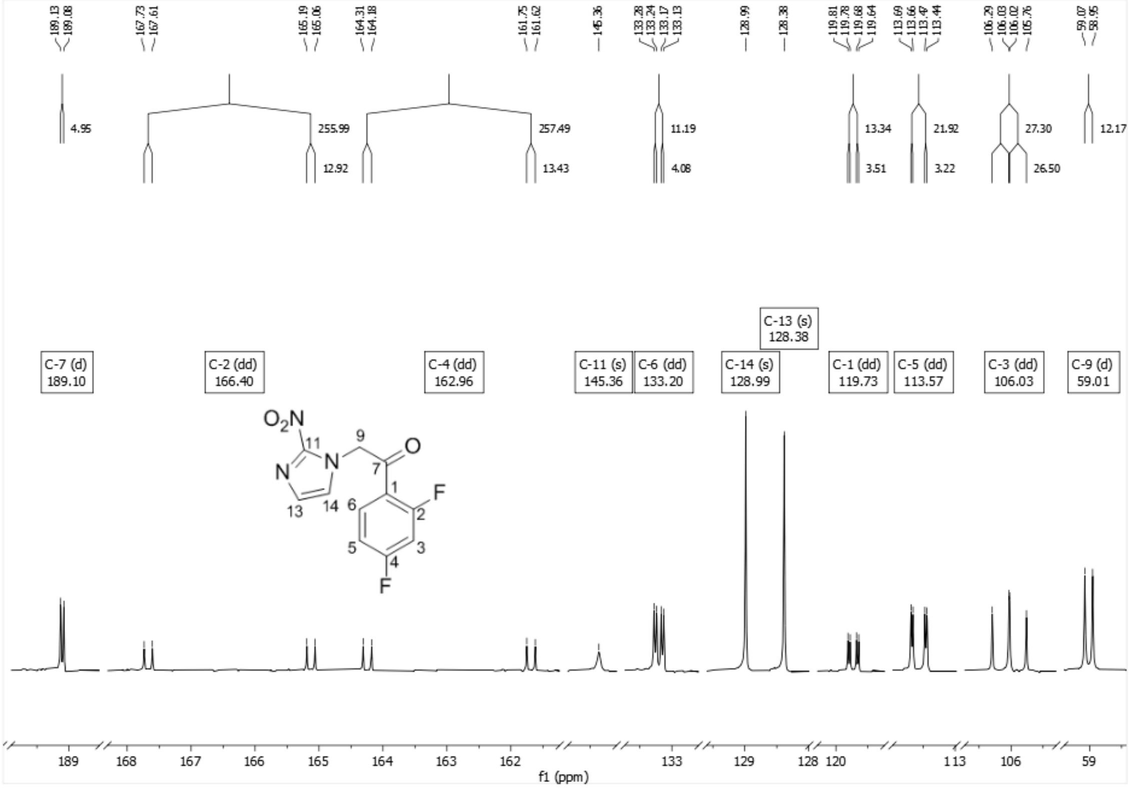

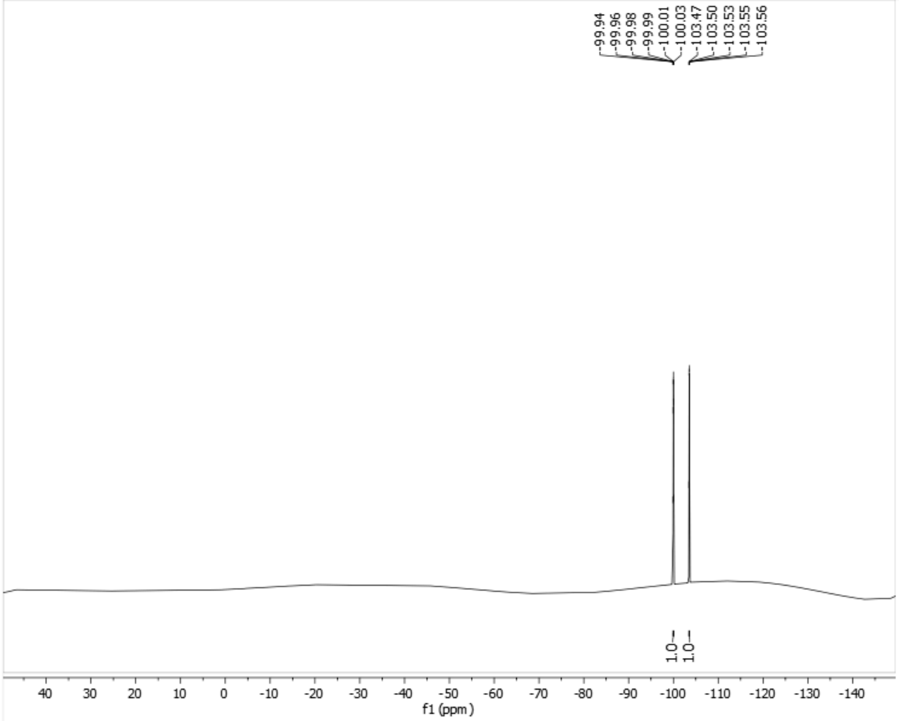

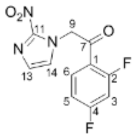

	Parameter	Value
1	Origin	Bruker BioSpin GmbH
2	Owner	nmr
3	Instrument	spect
4	Solvent	DMSO
5	Temperature	298.0
6	Pulse Sequence	zg30
7	Experiment	1D
8	Probe	5 mm CPPBBO BB-1H/ 19F/ D Z-GRD Z122623/ 0041
9	Number of Scans	32
10	Receiver Gain	32.3
11	Relaxation Delay	1.0000
12	Pulse Width	15.0000
13	Acquisition Time	0.8738
14	Acquisition Date	2016-03-03T07:34:00
15	Modification Date	2016-03-03T07:34:30
16	Spectrometer Frequency	376.52
17	Spectral Width	75000.0
18	Lowest Frequency	-56325.9
19	Nucleus	19F
20	Acquired Size	65536
21	Spectral Size	131072
22	Digital Resolution	0.57

^{19}F NMR (377 MHz, DMSO-d_6) δ -99.99 (dt, J = 15.8, 8.2 Hz), -103.45 – -103.57 (m).

Determinação de pureza - série PRCH

Sistema de Cromatografia a Líquido Agilent 1100 Series com bomba binária G1312A, degaseificador G1322A, injetor automático G1313A, forno de colunas G1316A e detector por arranjo de diodos G1315B. Aquisição de dados e controle pelo software Agilent ChemStation 1100 Series.

Coluna: XDB C18 – 150 x 4,6 mm; 5 µm
Pré-coluna: XDB C18 – 12,5 x 4,6 mm; 5 µm

Fase Móvel
Eluente (A): Tampão Acetato de amônio 5 mM, pH 5 **Eluente (B):** Acetonitrila

Programação do gradiente: De 0 a 25 min - 10 a 90% de B, 25 a 30 min - 90% de B, 30 a 35 min - 90 a 10% de B, 35 a 40 min mantendo 10% de B.

Temperatura do forno: 25 °C
Vazão: 1,0 mL min^{-1}

Procedimento geral para preparo de amostra: A 1 mg de amostra, foram adicionados 1000 µL de acetonitrila, seguido de banho ultrassônico por 2-5 min. Alíquotas de 400 µL de solução foram transferidas para balões volumétricos de 2,0 mL e avolumadas com metanol (concentração: 200 µg mL^{-1}). Posteriormente, as soluções foram filtradas em membrana PVDF 0,45 µm e injetadas (20 µL).

Amostra: Branco

Perfil Cromatográfico (λ = 225 nm)

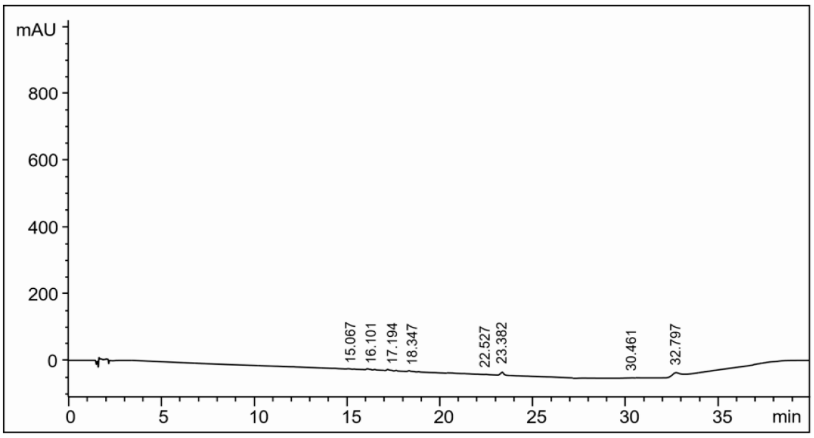

Branco (225 nm, 4 nm)

Tr (min)	Área	% Área
15,067	14,6423	2,65
16,101	40,805	7,38
17,194	50,503	9,13
18,347	33,7213	6,10
22,527	5,42051	0,98
23,382	90,0231	16,28
30,461	25,2882	4,57
32,797	292,705	52,92
	553,1084	100,00

Obs: Os picos identificados na corrida do branco foram descontados em todas as amostras seguintes.

LaQAFA
Laboratório de Química Analítica Fundamental e Aplicada

Amostra: PRCH 043/15

Perfil Cromatográfico (λ = 225 nm)

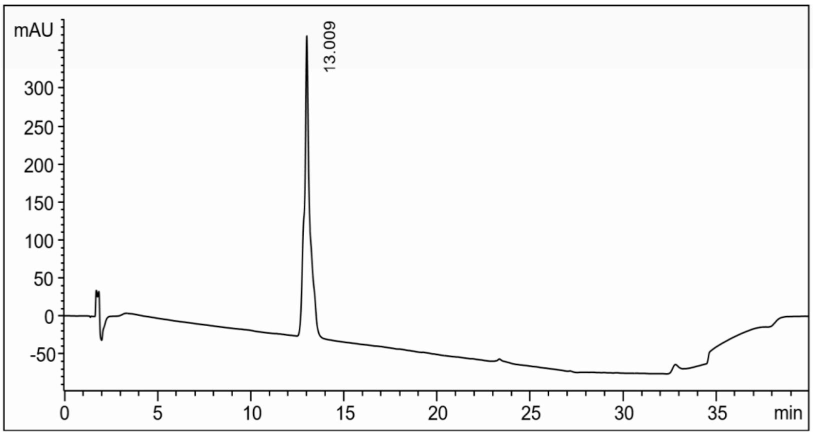

PRCH 043/15 (225 nm)

Tr (min)	Área	% Área
13,01	7990,1	**100,0**
	7990,1	100,0

Perfil Cromatográfico (λ = 245 nm)

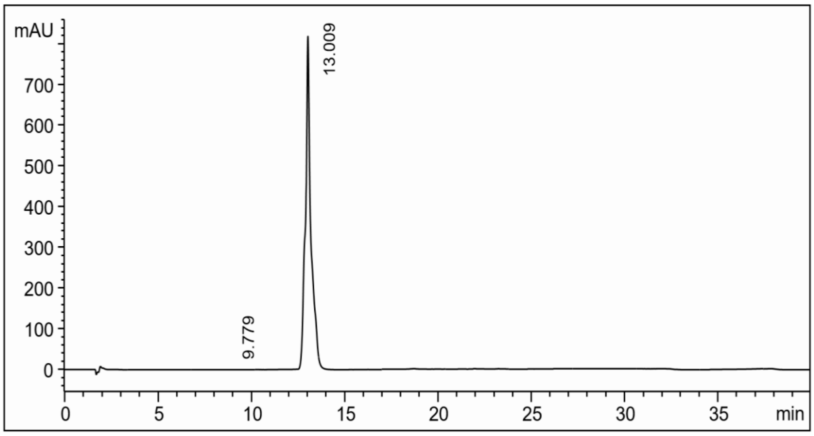

PRCH 043/15 (245 nm)		
**Tr (min)**	**Área**	**% Área**
9,78	26,746	0,16
13,01	16642,6	**99,84**
	16669,3	100,0

Perfil Cromatográfico (λ = 265 nm)

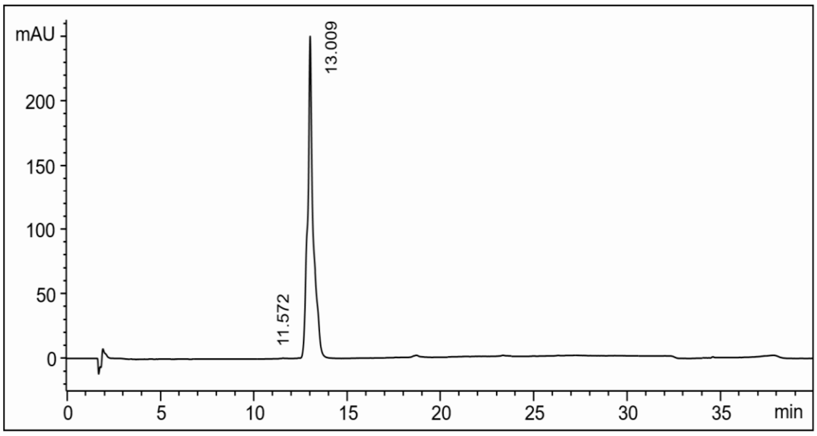

PRCH 043/15 (265 nm)		
**Tr (min)**	**Área**	**% Área**
11,57	7,711	0,15
13,01	5077,2	**99,85**
	5084,9	100,0

Perfil Cromatográfico (λ = 290 nm)

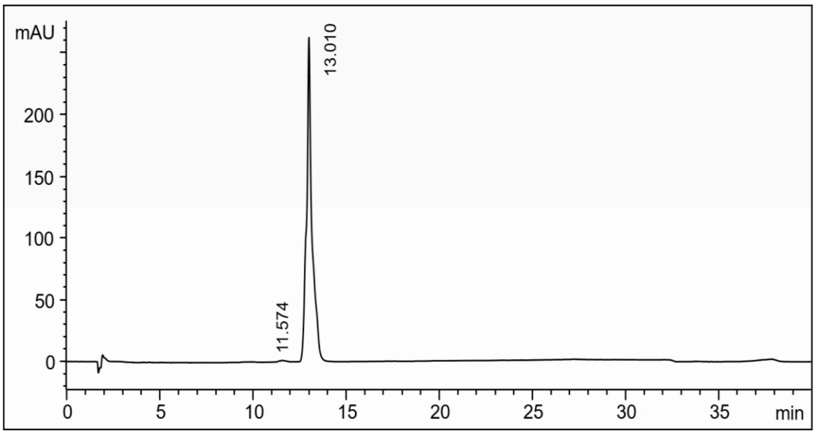

1-(2,4-difluorphenyl)-2-(1 *H*-1,2,4-triazol-1-yl)ethanone (7)

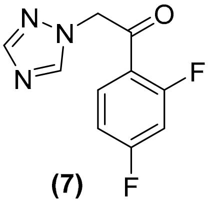

(7)

Compound Spectrum SmartFormula Report

Analysis Info

Analysis Name	D:\Data\2022\SINTESE 1\INFUSAO\2351-22 - TZ 045-22.d	Acquisition Date	6/20/2022 9:57:52 AM
Method	Tune_pos_Standard.m	Operator	ALEXANDRE
Sample Name	TZ 045-22	Instrument	compact 8255754.10035
Comment			

Acquisition Parameter

Source Type	ESI	Ion Polarity	Positive	Set Nebulizer	0.4 Bar
Focus	Not active	Set Capillary	4000 V	Set Dry Heater	200 °C
Scan Begin	100 m/z	Set End Plate Offset	-400 V	Set Dry Gas	4.0 l/min
Scan End	1000 m/z	Set Charging Voltage	2000 V	Set Divert Valve	Source
		Set Corona	0 nA	Set APCI Heater	0 °C

+MS, 0.2-0.3min #9-16

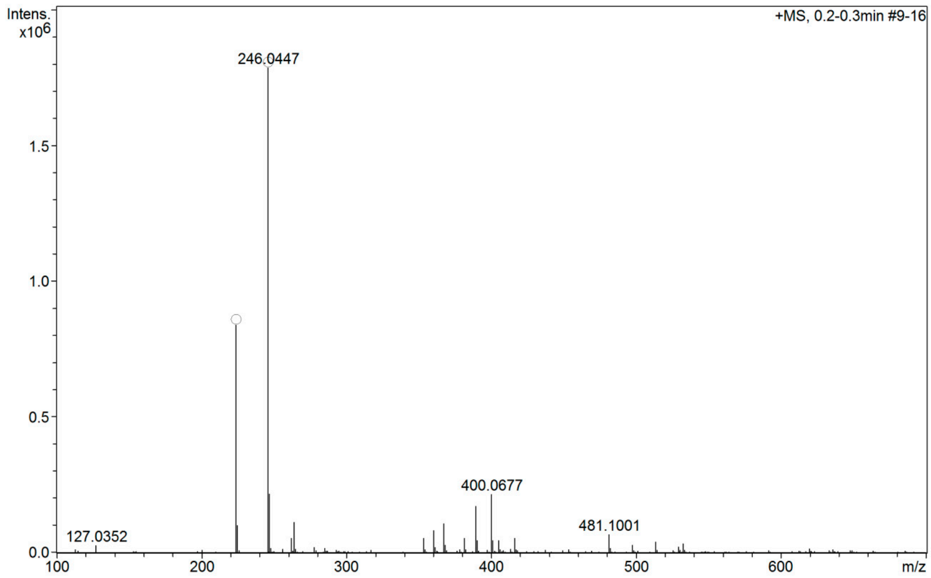

Meas. m/z	#	Ion Formula	m/z	err [ppm]	Mean err [ppm]	rdb	N-Rule	e⁻ Conf	mSigma	Std I	Std Mean m/z	Std I VarNorm	Std m/z Diff	Std Comb Dev
224.062941	1	C10H8F2N3O	224.062995	0.2	0.2	7.5	ok	even	0.7	1.4	n.a.	n.a.	n.a.	n.a.
246.044714	1	C10H7F2N3NaO	246.044939	0.9	-0.6	7.5	ok	even	0.7	1.7	n.a.	n.a.	n.a.	n.a.

Library Search Report - ChemStation Integrator

Unknown Spectrum based on Apex

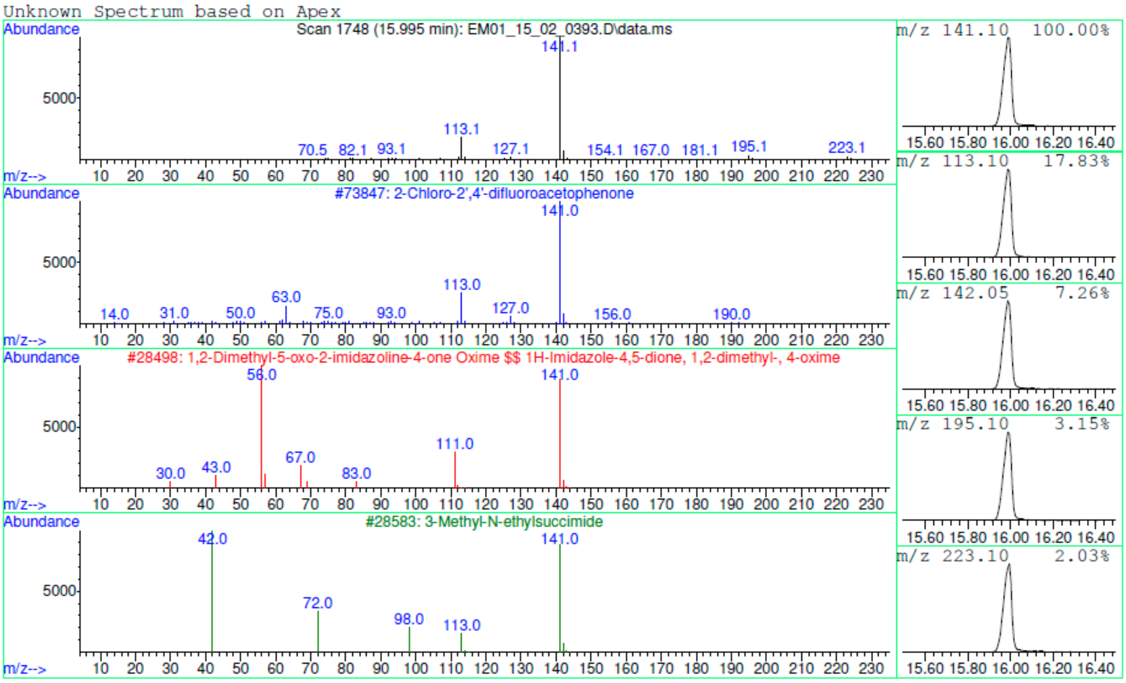

Data File: D:\Backup dos Sistemas\CG-EM-01\2015\02_FEVEREIRO_15\EM01_15_02_0393.D
Sample : PRCH009-15

Peak Number: 1 at 15.995 min Area: 106223675 Area % 100.00

The 3 best hits from each library.	Ref\#	CAS\#	Qual
C:\Database\WILEY275.L			
1 2-Chloro-2',4'-difluoroacetophenone	73847	051336-94-8	40
2 1,2-Dimethyl-5-oxo-2-imidazoline...	28498	110578-75-1	9
3 3-Methyl-N-ethylsuccimide	28583	065168-02-7	9

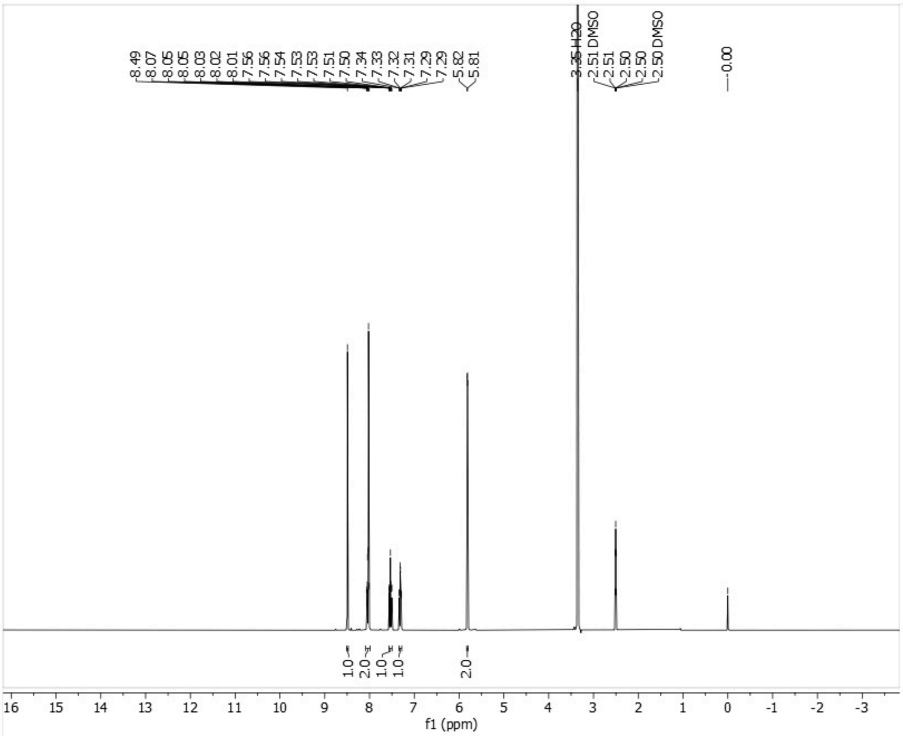

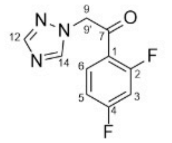

Parameter		Value
1	Origin	Bruker BioSpin GmbH
2	Owner	nmrsu
3	Instrument	spect
4	Solvent	DMSO
5	Temperature	298.0
6	Pulse Sequence	zg30
7	Experiment	1D
8	Probe	Z122623_0041 (CPP BBO 400S1 BB-H&F-D-05 Z)
9	Number of Scans	32
10	Receiver Gain	149.0
11	Relaxation Delay	1.0000
12	Pulse Width	12.4000
13	Acquisition Time	4.0894
14	Acquisition Date	2021-12-06T05:47:16
15	Modification Date	2021-12-06T05:47:18
16	Spectrometer Frequency	400.26
17	Spectral Width	8012.8
18	Lowest Frequency	-1535.7
19	Nucleus	1H
20	Acquired Size	32768
21	Spectral Size	65536
22	Digital Resolution	0.12

^{1}H NMR (400 MHz, DMSO-d_6) δ 8.49 (s, 1H), 8.04 (td, J = 8.6, 6.6 Hz, 1H), 8.02 (s, 1H), 7.53 (ddd, J = 11.7, 9.3, 2.5 Hz, 1H), 7.31 (td, J = 8.4, 2.3 Hz, 1H), 5.82 (d, J = 3.0 Hz, 2H).

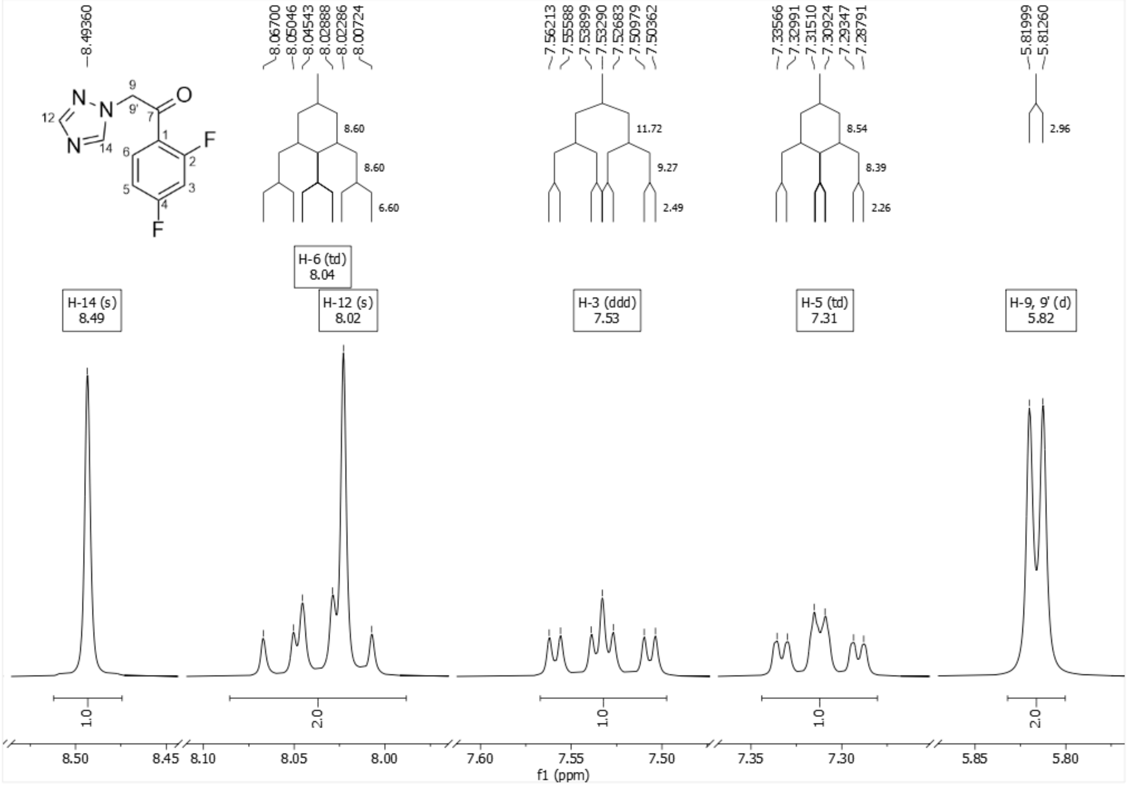

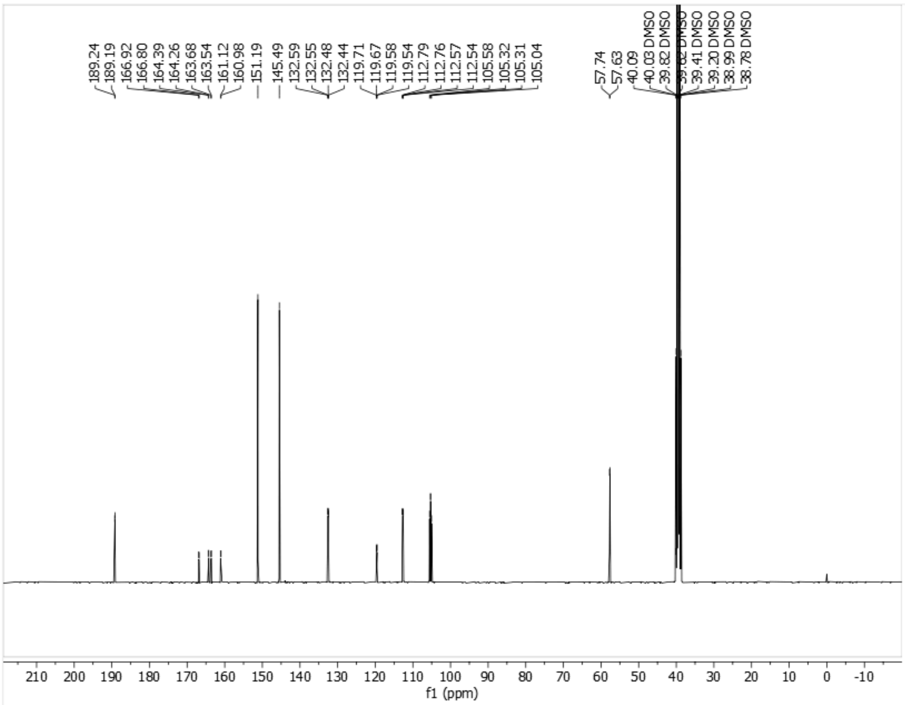

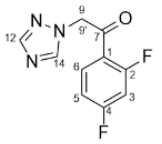

	Parameter	Value
1	Origin	Bruker BioSpin GmbH
2	Owner	nmrsu
3	Instrument	spect
4	Solvent	DMSO
5	Temperature	298.0
6	Pulse Sequence	zgpg30
7	Experiment	1D
8	Probe	Z122623_0041 (CPP BBO 400S1 BB-H&F-D-05 Z)
9	Number of Scans	12288
10	Receiver Gain	46.5
11	Relaxation Delay	1.0000
12	Pulse Width	11.2500
13	Acquisition Time	1.3631
14	Acquisition Date	2021-12-06T05:43:11
15	Modification Date	2021-12-07T09:16:12
16	Spectrometer Frequency	100.66
17	Spectral Width	24038.5
18	Lowest Frequency	-2011.6
19	Nucleus	13C
20	Acquired Size	32768
21	Spectral Size	65536
22	Digital Resolution	0.37

^{13}C NMR (101 MHz, DMSO-d_6) δ 189.21 (d, J = 5.0 Hz), 165.59 (dd, J = 255.3, 12.8 Hz), 162.33 (dd, J = 257.8, 13.5 Hz), 151.19, 145.49, 132.52 (dd, J = 11.1, 4.1 Hz), 119.62 (dd, J = 13.2, 3.6 Hz), 112.67 (dd, J = 21.7, 3.3 Hz), 105.31 (dd, J = 27.3, 26.5 Hz), 57.69 (d, J = 10.8 Hz).

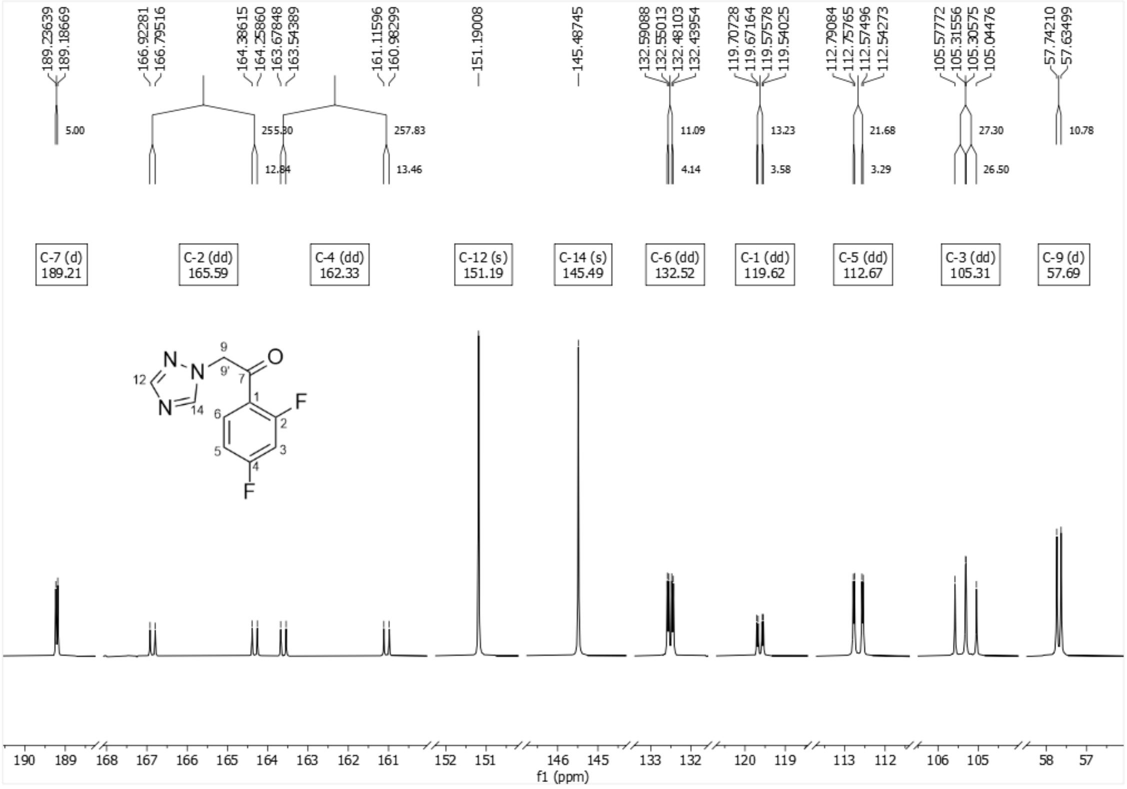

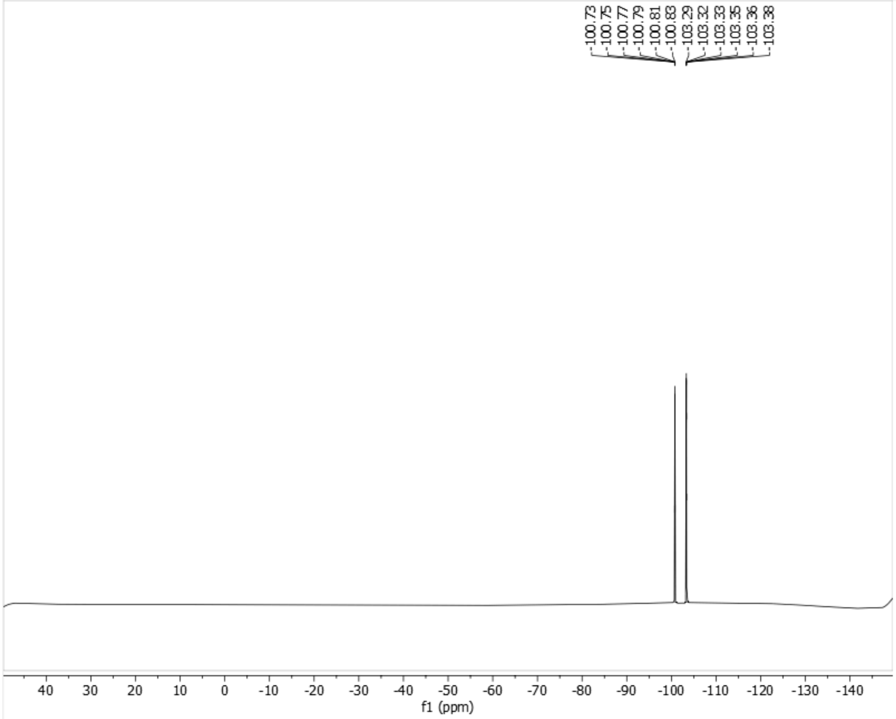

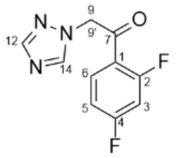

	Parameter	Value
1	Origin	Bruker BioSpin GmbH
2	Owner	nmrsu
3	Instrument	spect
4	Solvent	DMSO
5	Temperature	298.0
6	Pulse Sequence	zg30
7	Experiment	1D
8	Probe	Z122623_0041 (CPP BBO 400S1 BB-H&F-D-05 Z)
9	Number of Scans	64
10	Receiver Gain	28.4
11	Relaxation Delay	1.0000
12	Pulse Width	15.0000
13	Acquisition Time	0.8738
14	Acquisition Date	2021-12-06T05:51:04
15	Modification Date	2021-12-06T05:51:06
16	Spectrometer Frequency	376.60
17	Spectral Width	75000.0
18	Lowest Frequency	-56331.0
19	Nucleus	19F
20	Acquired Size	65536
21	Spectral Size	131072
22	Digital Resolution	0.57

^{19}F NMR (377 MHz, DMSO-d_6) δ -100.78 (dq, J = 15.9, 8.0 Hz), -103.34 (td, J = 12.2, 8.7 Hz).

Farmanguinhos - Fiocruz
Serviço de Métodos Analíticos

TZ 045-22 - Rep 1.lcd
2022 - Sintese 1 - Rodolfo França - 2-7/2-11-5 - TZ 045-22 - Rep 1.lcd
04/07/2022, 17:49:14

Sample Name : TZ 045-22 - Rep 1
Sample ID : LC 0313
Sample Full Path : 2022 - Sintese 1 - Rodolfo França - 2-7/2-11-5 - TZ 045-22 - Rep 1.lcd
Data Filename : TZ 045-22 - Rep 1.lcd
Method Filename : Difluorofeniltriazol_1.lcm
Batch File : 2022 - Sintese 1 - Rodolfo França - CLAE-10 - CLAE 10 - 2022_0704.lcb
Vial # : 1-2 Sample Type : Unknown
Injection Volume : 20 uL
Date Acquired : 04/07/2022 17:49:44 Acquired by : Marcio Soares
Date Processed : 05/07/2022 09:28:49 Processed by : Marcio Soares
Data Comment : Método: Difluorofeniltriazol_1

Preparo da Amostra: Em 2mg de amostra, foram adicionados 2000µL de acetonitrila, seguido de banho ultrassônico por 5 minutos. Uma aliquota de 400µL de solução foram transferidas para balão volumétrico de 2,0mL e avolumado com metanol. Posteriormente filtrada em membrana PVDF de 0,22µm e injetada. Protocolo enviado pelo solicitante.

Pré coluna : Supelguard LC-18 2cm x 2,1mm , 5µm

Method

<<Header>>

<<Pump>>
Total Flow : 1,0000 mL/min
Pump A : LC-20ADXR

<<Mobile Phase Name>>
Pump A Mobile Phase A : Acetato de NH4-5mM - pH5,0
Pump A Mobile Phase C : Acetonitrila

<<LC Time Program>>

Time	Module	Command	Value	Comment
0,01	Pumps	Solvent C Conc.	10	
0,01	Pumps	Total Pump A Flow	1	
25,00	Pumps	Solvent C Conc.	90	
30,00	Pumps	Solvent C Conc.	90	
35,00	Pumps	Solvent C Conc.	10	
40,00	Pumps	Solvent C Conc.	10	
40,01	Controller	Stop		

<<Data Acquisition>>
LC Stop Time : 40,01 min

<<Oven>>
Oven Temperature : 25 C

Instrumentation

<<Instrument>>

<<Pump A>>
Pump A Type : LC-20ADXR

<<System Controller>>
SCL Type : CBM-20A

<<Autosampler>>
Autosampler : SIL-30AC

<<Oven>>
Oven Type : CTO-20AC

<<Column>>
Column Name : Hypersil BDS - C18 5 µm
Column ID : CL 0021
Length : 150 mm
Internal Diameter : 4,6 mm
Max Number of Injections : 9999
Expiration Date : 28/04/2015
Max Temperature : 80,0 C
Installation Date : 28/04/2030

<<PDA>>
Detector Name : PDA
Detector Type : SPD-M20A

<<Degasser>>
Degasser : DGU-20A5R

Farmanguinhos - Fiocruz
Serviço de Métodos Analíticos

TZ 045-22 - Rep 1.lcd
2022 - Sintese 1 - Rodolfo França - 2-7/2-11-5 - TZ 045-22 - Rep 1.lcd
04/07/2022, 17:49:14

Chromatogram

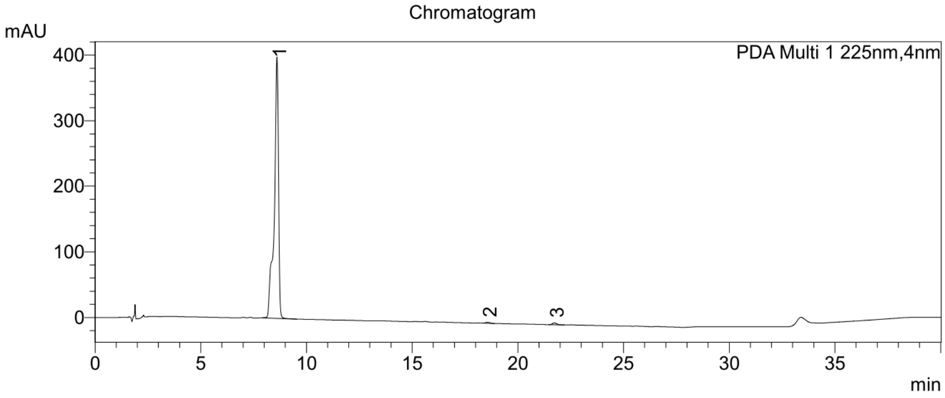

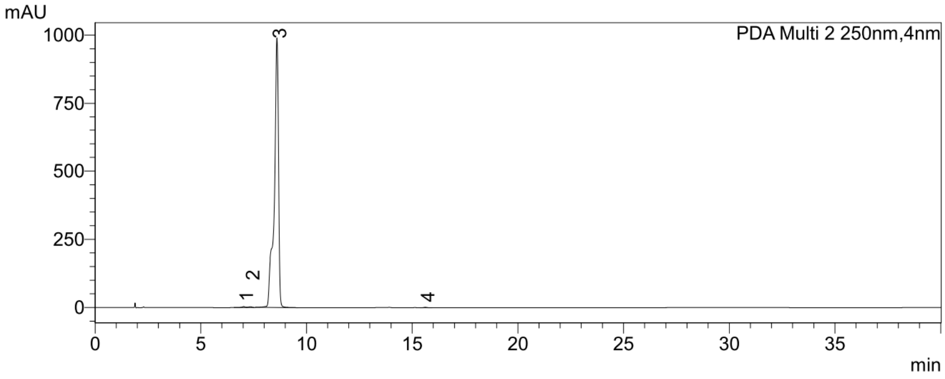

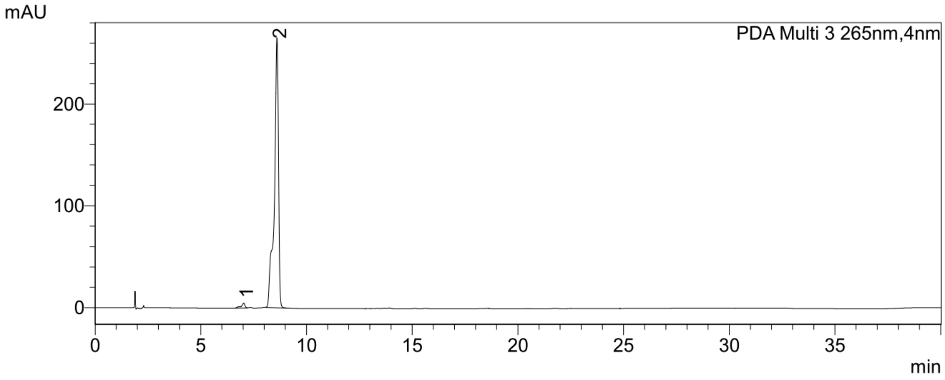

Farmanguinhos - Fiocruz
Serviço de Métodos Analíticos

TZ 045-22 - Rep 1.lcd
2022 - Sintese 1 - Rodolfo França - 2-7/2-11-5 - TZ 045-22 - Rep 1.lcd
04/07/2022, 17:49:14

Chromatogram

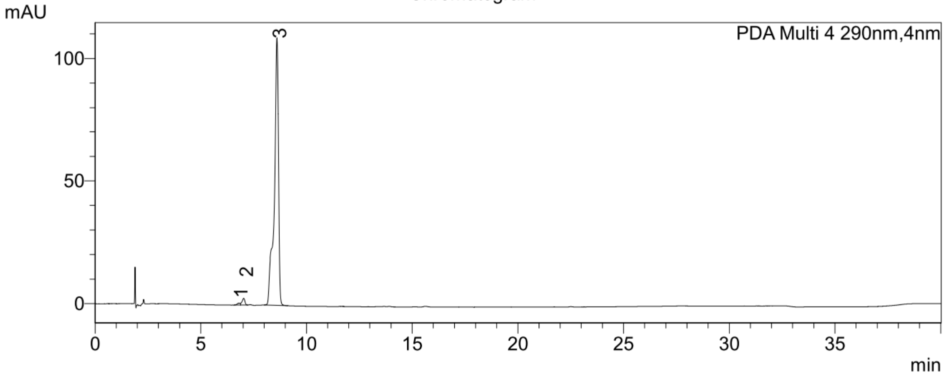

Peak Table

PDA Ch1 225nm

Peak#	Ret. Time	Name	Area	Area%	Theoretical Plates/meter(USP)	Tailing Factor	Resolution(USP)	Capacity Factor(k')
1	8,60		5504660	98,9	82057	0,698	--	--
2	18,56		20436	0,4	256486	1,223	28,905	1,157
3	21,73		38004	0,7	323543	1,177	8,202	1,525
Total			5563101	100,0				

PDA Ch2 250nm

Peak#	Ret. Time	Name	Area	Area%	Theoretical Plates/meter(USP)	Tailing Factor	Resolution(USP)	Capacity Factor(k')
1	7,04		37870	0,3	67220	--	--	--
2	7,34		26754	0,2	67433	--	1,076	0,044
3	8,60		13810039	99,4	81607	0,698	4,181	0,223
4	15,62		18076	0,1	261819	1,082	22,397	1,220
Total			13892739	100,0				

PDA Ch3 265nm

Peak#	Ret. Time	Name	Area	Area%	Theoretical Plates/meter(USP)	Tailing Factor	Resolution(USP)	Capacity Factor(k')
1	7,03		57250	1,6	76124	0,667	--	--
2	8,60		3618627	98,4	83899	0,697	5,511	0,223
Total			3675877	100,0				

PDA Ch4 290nm

Peak#	Ret. Time	Name	Area	Area%	Theoretical Plates/meter(USP)	Tailing Factor	Resolution(USP)	Capacity Factor(k')
1	6,77		7955	0,5	3956	--	--	--
2	7,03		25021	1,6	75802	--	0,377	0,038
3	8,60		1486364	97,8	83628	0,697	5,502	0,270
Total			1519340	100,0				

Farmanguinhos - Fiocruz
Serviço de Métodos Analíticos

TZ 045-22 - Rep 1.lcd
2022 - Sintese 1 - Rodolfo França - 2-7/2-11-5 - TZ 045-22 - Rep 1.lcd
04/07/2022, 17:49:14

UV Spectrum

Retention time: 8,604 min
Lambda max: 243/275/619/727 nm

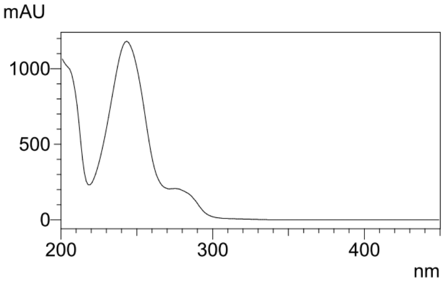

Retention time: 18,557 min
Lambda max: 257/484/397/592/622 nm

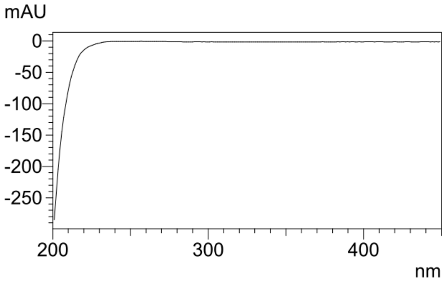

Retention time: 21,727 min
Lambda max: 239/484/404/592/619 nm

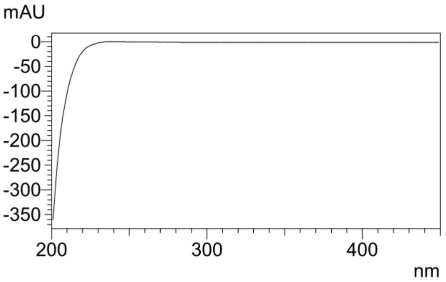

Retention time: 7,035 min
Lambda max: 268/655/528/421 nm

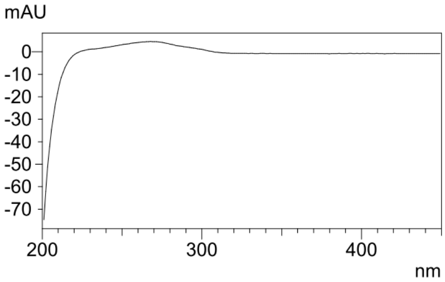

Retention time: 7,343 min
Lambda max: 244/522/655 nm

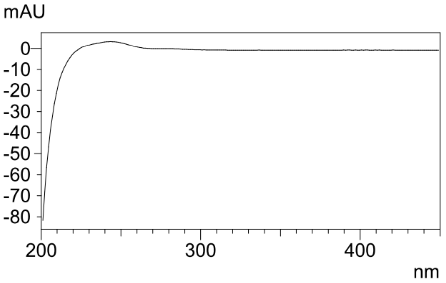

Retention time: 15,618 min
Lambda max: 242/507/619/591/421 nm

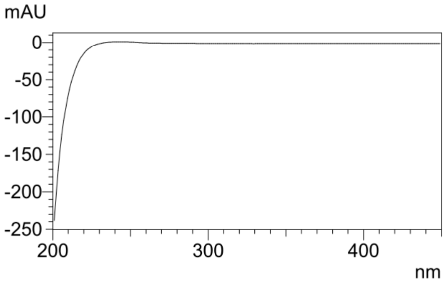

Farmanguinhos - Fiocruz
Serviço de Métodos Analíticos

TZ 045-22 - Rep 1.lcd
2022 - Sintese 1 - Rodolfo França - 2-7/2-11-5 - TZ 045-22 - Rep 1.lcd
04/07/2022, 17:49:14

Retention time: 6,773 min
Lambda max: 267/655/522 nm

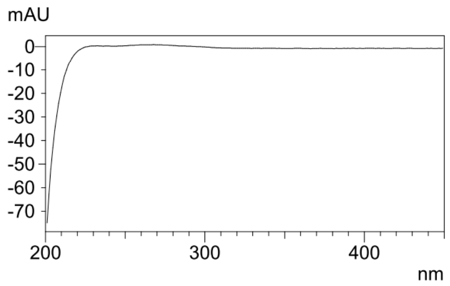

1-(2,4-difluorophenyl)-2-(3-nitro-1*H*-1,2,4-triazol-1-yl)ethanol (8)

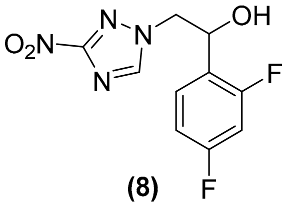

(8)

Compound Spectrum SmartFormula Report

Analysis Info

Analysis Name	D:\Data\2017\SINTESE 1\INFUSAO\108351 - PFCT 002-17.d	Acquisition Date	10/17/2017 2:03:15 PM
Method	Tune_pos_Standard.m	Operator	Alexandre
Sample Name	108351 - PFCT 002-17	Instrument	compact 8255754.10035
Comment			

Acquisition Parameter

Source Type	ESI	Ion Polarity	Positive	Set Nebulizer	0.4 Bar
Focus	Not active	Set Capillary	4000 V	Set Dry Heater	200 °C
Scan Begin	80 m/z	Set End Plate Offset	-400 V	Set Dry Gas	6.0 l/min
Scan End	1000 m/z	Set Charging Voltage	2000 V	Set Divert Valve	Source
		Set Corona	0 nA	Set APCI Heater	0 °C

+MS, 0.1-0.3min #4-17

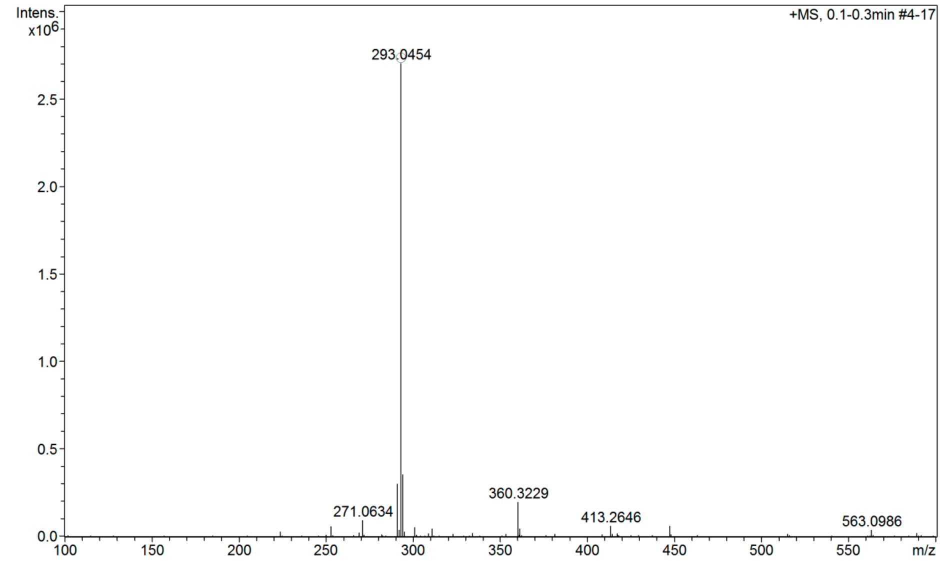

Meas. m/z	#	Ion Formula	m/z	err [ppm]	Mean err [ppm]	rdb	N-Rule	e⁻ Conf	mSigma	Std I	Std Mean m/z	Std I VarNorm	Std m/z Diff	Std Comb Dev
293.045361	1	C10H8F2N4NaO3	293.045667	1.0	1.9	7.5	ok	even	3.8	6.0	n.a.	n.a.	n.a.	n.a.
	2	C9H12F2NaO7	293.044330	-3.5	-2.0	2.5	ok	even	17.6	30.1	n.a.	n.a.	n.a.	n.a.

Library Search Report - ChemStation Integrator

Unknown Spectrum based on Apex

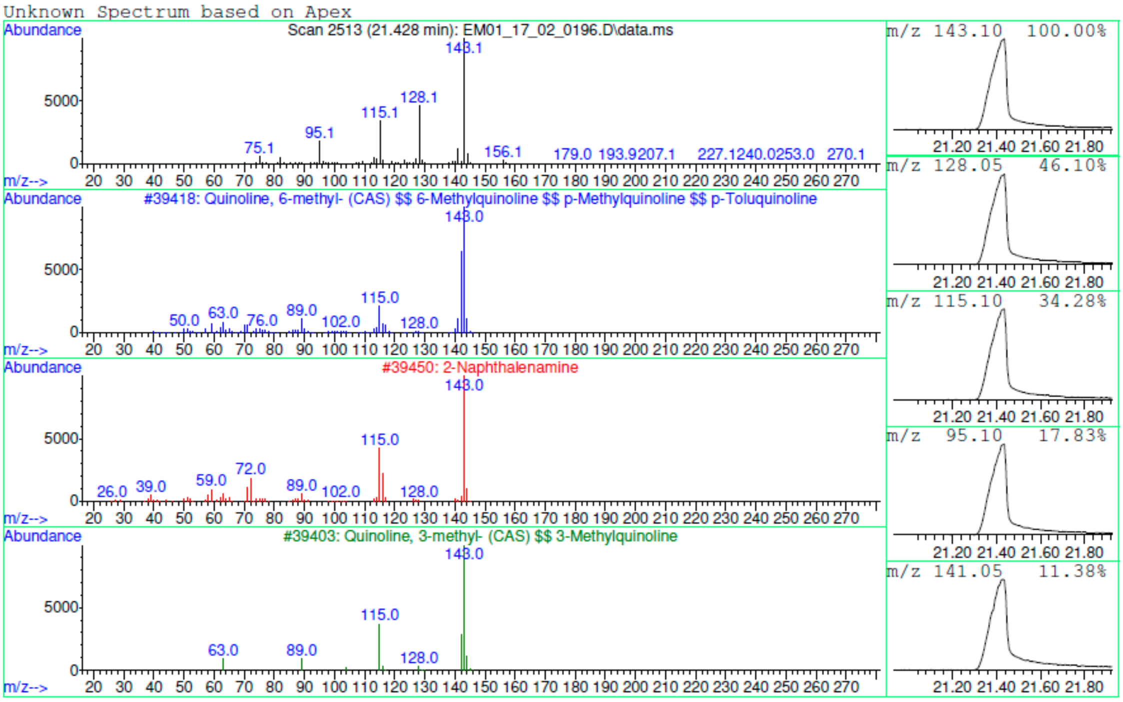

Data File: C:\Users\FFAR-21014\Documents\BACKUP DOS SISTEMAS\CG-EM-01\2017\02_FEVEREIRO_17\EM01_
... 17_02_0196.D
Sample : PFCT 002-17

Peak Number: 1 at 21.428 min Area: 296779786 Area % 100.00

The 3 best hits from each library.	Ref\#	CAS\#	Qual
C:\Database\wiley7n.l			
1 Quinoline, 6-methyl- (CAS) $$ 6-...	39418	000091-62-3	50
2 2-Naphthalenamine	39450	000091-59-8	47
3 Quinoline, 3-methyl- (CAS) $$ 3-...	39403	000612-58-8	43

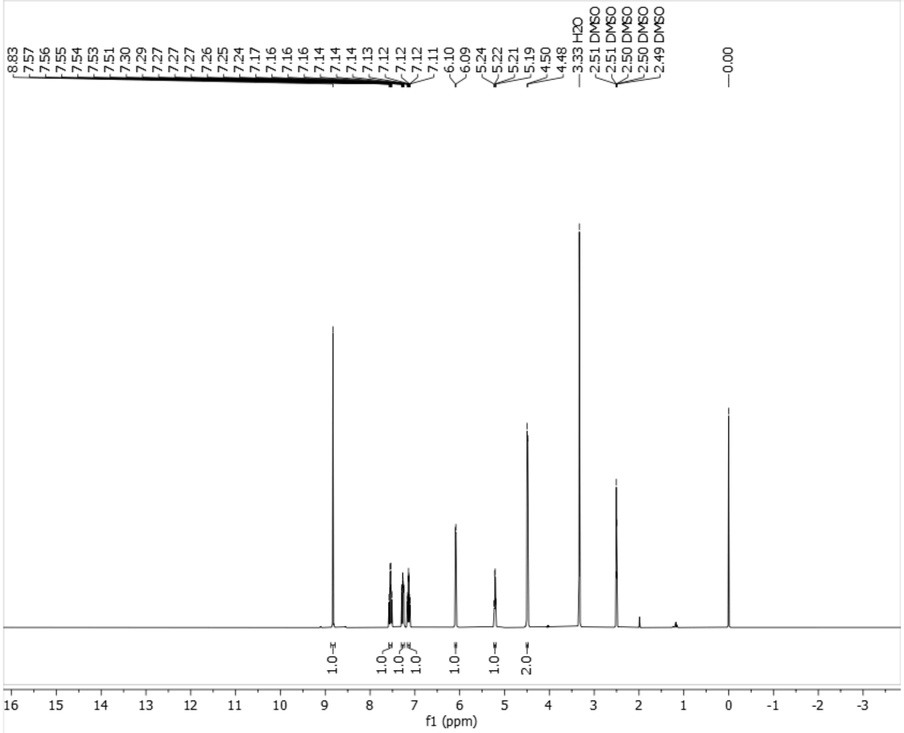

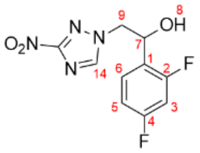

	Parameter	Value
1	Origin	Bruker BioSpin GmbH
2	Owner	nmr
3	Instrument	spect
4	Solvent	DMSO
5	Temperature	298.0
6	Pulse Sequence	zg30
7	Experiment	1D
8	Probe	5 mm CPPBBO BB-1H/ 19F/ D Z-GRD Z122623/ 0041
9	Number of Scans	32
10	Receiver Gain	149.0
11	Relaxation Delay	1.0000
12	Pulse Width	11.5000
13	Acquisition Time	2.0447
14	Acquisition Date	2017-02-02T15:27:00
15	Modification Date	2018-07-19T19:35:13
16	Spectrometer Frequency	400.15
17	Spectral Width	8012.8
18	Lowest Frequency	-1537.1
19	Nucleus	1H
20	Acquired Size	16384
21	Spectral Size	65536
22	Digital Resolution	0.12

^{1}H NMR (400 MHz, DMSO-d_6) δ 8.83 (s, 1H), 7.54 (td, J = 8.6, 6.6 Hz, 1H), 7.27 (ddd, J = 10.8, 9.3, 2.5 Hz, 1H), 7.14 (tdd, J = 8.5, 2.5, 1.0 Hz, 1H), 6.09 (d, J = 4.9 Hz, 1H), 5.21 (q, J = 5.8 Hz, 1H), 4.49 (d, J = 6.1 Hz, 2H).

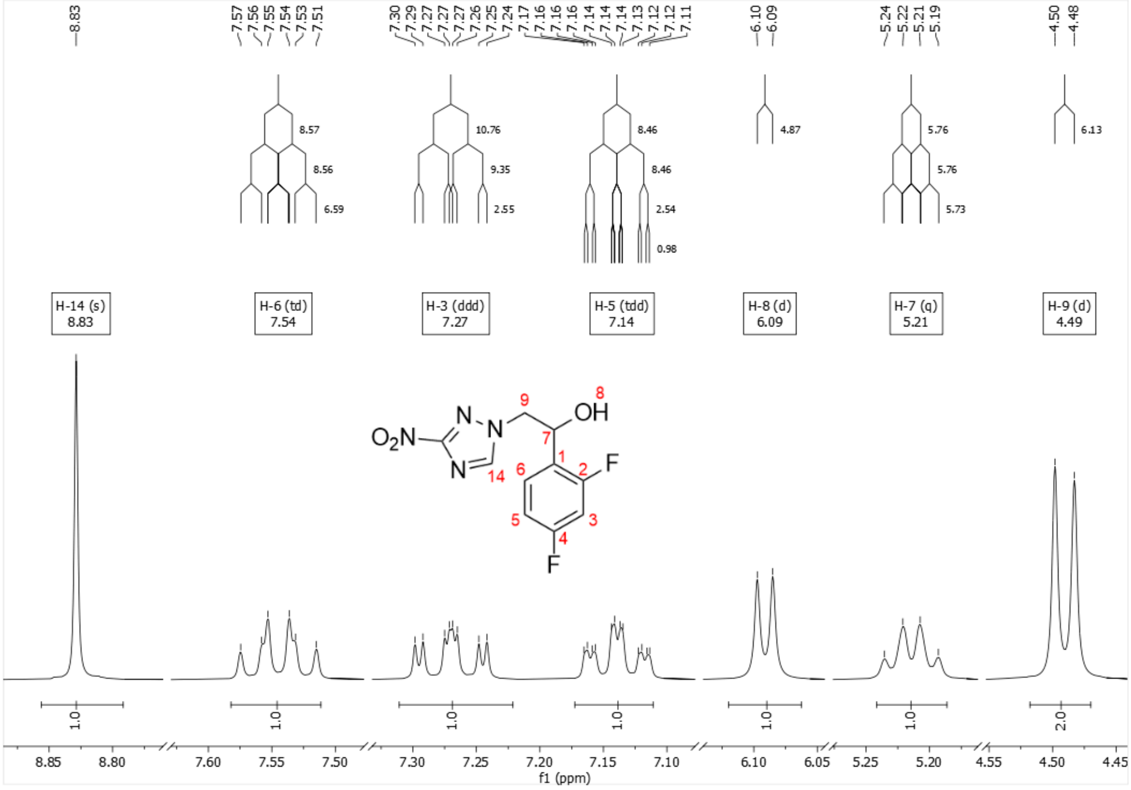

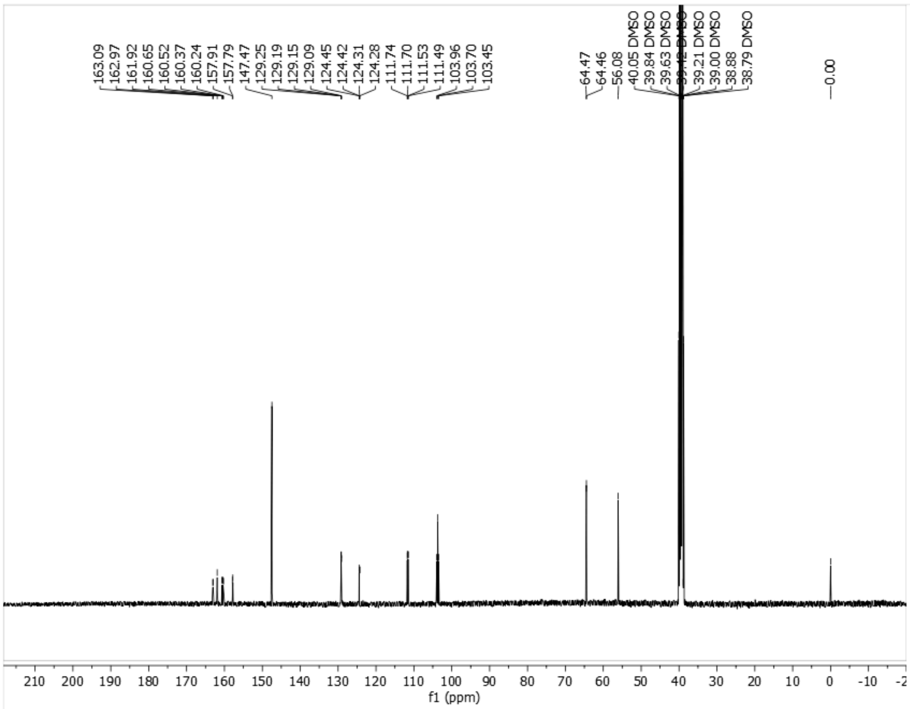

Parameter	Value
1 Origin	Bruker BioSpin GmbH
2 Owner	nmr
3 Instrument	spect
4 Solvent	DMSO
5 Temperature	298.0
6 Pulse Sequence	zgpg30
7 Experiment	1D
8 Probe	5 mm CPPBBO BB-1H/ 19F/ D Z-GRD Z122623/ 0041
9 Number of Scans	2048
10 Receiver Gain	46.5
11 Relaxation Delay	2.0000
12 Pulse Width	10.0000
13 Acquisition Time	0.6816
14 Acquisition Date	2017-02-02T16:13:00
15 Modification Date	2018-07-21T15:54:51
16 Spectrometer Frequency	100.63
17 Spectral Width	24038.5
18 Lowest Frequency	-2015.1
19 Nucleus	13C
20 Acquired Size	16384
21 Spectral Size	65536
22 Digital Resolution	0.37

^{13}C NMR (101 MHz, DMSO-d_6) δ 161.92, 161.81 (dd, J = 246.3, 12.3 Hz), 159.08 (dd, J = 247.2, 12.5 Hz), 147.47, 129.17 (dd, J = 10.0, 6.0 Hz), 124.37 (dd, J = 14.1, 3.6 Hz), 111.62 (dd, J = 21.2, 3.5 Hz), 103.70 (t, J = 26.0 Hz), 64.47 (d, J = 1.7 Hz), 56.08.

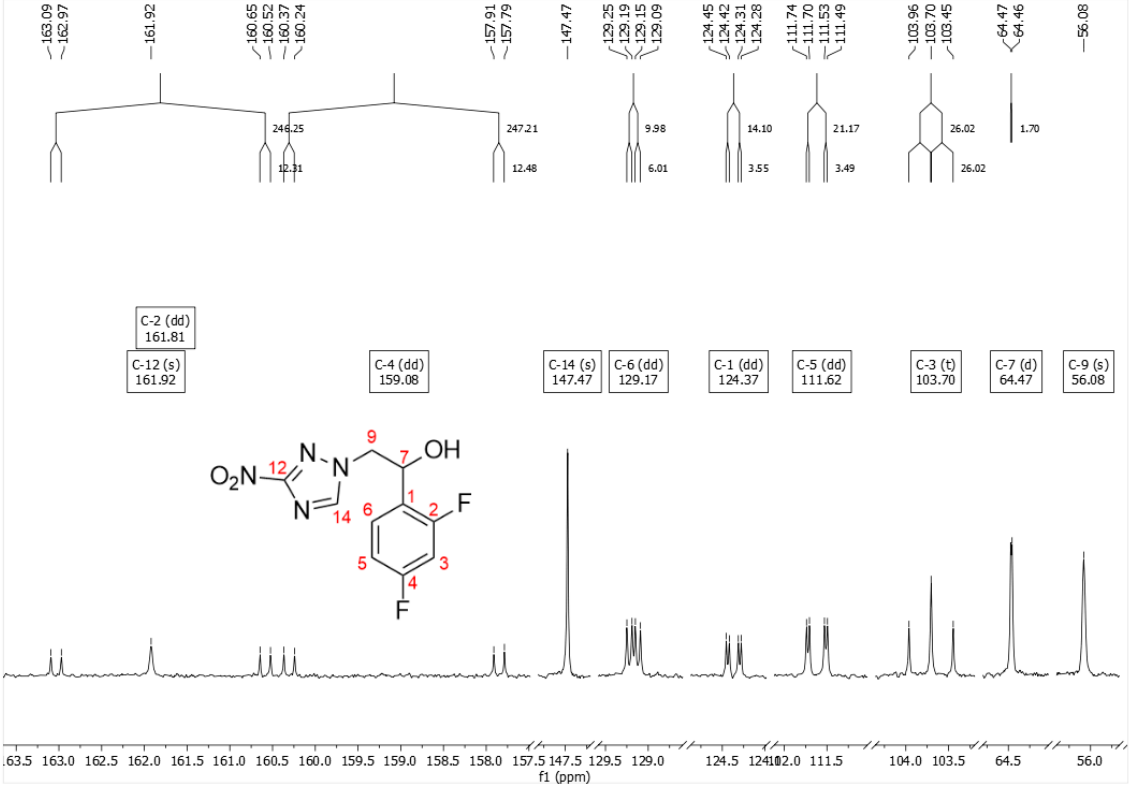

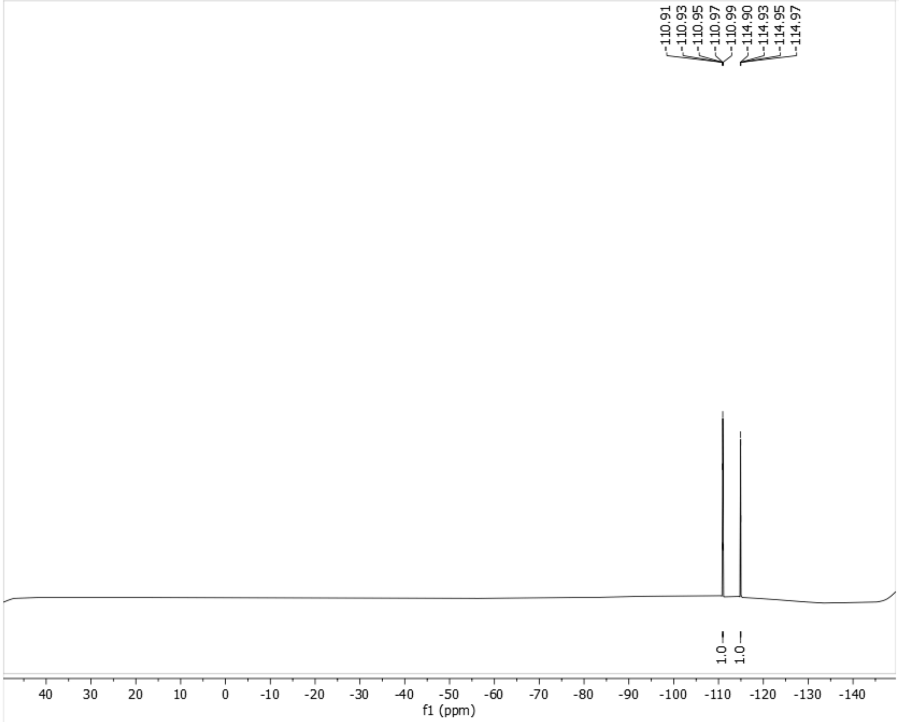

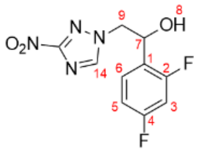

	Parameter	Value
1	Origin	Bruker BioSpin GmbH
2	Owner	nmrsu
3	Instrument	spect
4	Solvent	DMSO
5	Temperature	298.0
6	Pulse Sequence	zg30
7	Experiment	1D
8	Probe	Z122623_0041 (CPP BBO 400S1 BB-H&F-D-05 Z)
9	Number of Scans	256
10	Receiver Gain	25.4
11	Relaxation Delay	1.0000
12	Pulse Width	15.0000
13	Acquisition Time	0.8738
14	Acquisition Date	2021-08-05T10:50:32
15	Modification Date	2021-08-05T10:50:34
16	Spectrometer Frequency	376.60
17	Spectral Width	75000.0
18	Lowest Frequency	-56331.0
19	Nucleus	19F
20	Acquired Size	65536
21	Spectral Size	131072
22	Digital Resolution	0.57

^{19}F NMR (377 MHz, DMSO-d_6) δ -110.95 (dt, J = 16.0, 8.6, 7.8 Hz), -114.94 (qd, J = 10.5, 8.6, 1.0 Hz).

SERVIÇO DE MÉTODOS ANALÍTICOS

SAMPLE DESCRIPTION: PFCT 002-17
INJECTION VOLUME: 20 **SEQUENCE:** C:\CLASS-VP\Sequence\2017\Síntese\Frederico Branco\CLAE 02 - 20171113.seq **VIAL:** 3
METHOD: C:\CLASS-VP\Methods\2017\Sintese 1\Frederico Branco\Difluorofeniltriazol 1.met
FILE NAME: C:\CLASS-VP\Data\2017\Síntese\Frederico Branco\11-17\PFCT 002-17 rep2-Rep1
SAMPLE ID: PFCT 002-17 rep2 **ACQUIRED:** 13/11/2017 22:19:08 **PRINTED:** 14/11/2017 10:59:14
ANALISTA: Leandro/Thiago

Sistema de Cromatografia a Líquido: Shimadzu com 2 bombas LC-10AD, degaseificador DGU-12A, injetor automático SIL-10AD, forno de colunas CTO-10A, detector por arranjo de diodos SPD-M10A. Os dados foram adquiridos por interface SCL-10A. Aquisição de dados e controle pelo software Shimadzu CLASS-VP versão 6.13 SP2.

Coluna: Hypersil BDS-C18 150x4,6mm, 5µm - (CL0021)
Pré-Coluna: Supelguard C18 - 2cm

Fase Móvel
Eluente (A): Tampão Acetato de amônio 5mM pH5,0
Eluente (B): Acetonitrila

Programação:

Time	Module	Command	Value
0,01	Pumps	Pump B	Conc. 10
25,00	Pumps	Pump B	Conc. 90
30,00	Pumps	Pump B	Conc. 90
35,00	Pumps	Pump B	Conc. 10
40,00	Pumps	Pump B	Conc. 10
40,01	Controller	Stop	

Temperatura do forno: 25ºC **Vazão:** 1,0 mL/min

Preparo da amostra:
A 1mg de amostra, foram adicionados 1000µL de acetonitrila, seguindo de banho ultrassônico por 5 min. Uma alíquota de 400µL de solução foi transferida para um balão volumétrico de 2mL e avolumado com metanol(concentração: 200µg/mL-1). Posteriormente a solução foi filtrada em membrana PVDF 0,22µm e injetada.

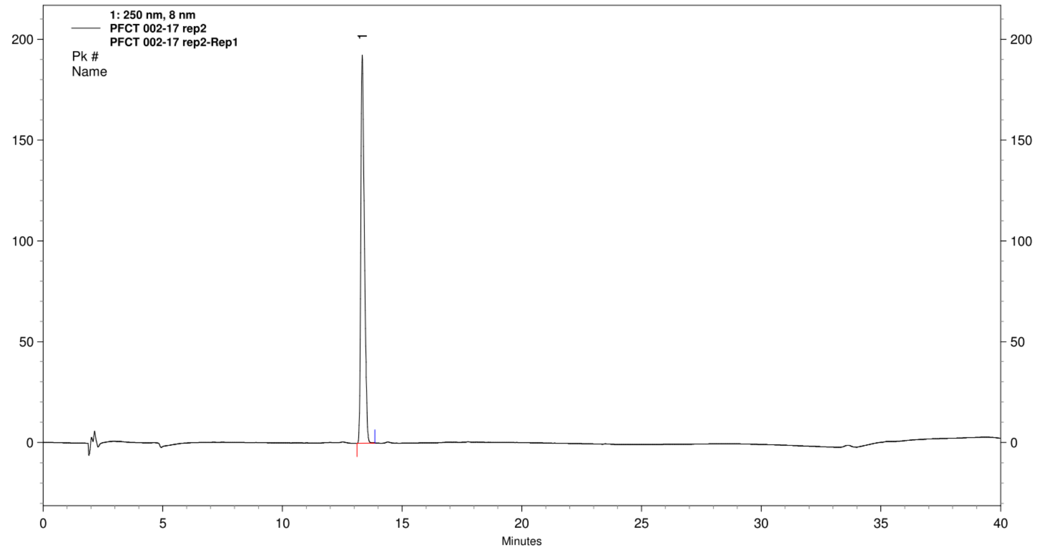

1: 250 nm, 8 nm

Pk #	Retention Time	Area	Area %	Height	Height %	Width
1	13.333	1993412	100.000	192462	100.000	0.75

Totals						
		1993412	100.000	192462	100.000	

SERVIÇO DE MÉTODOS ANALÍTICOS

SAMPLE DESCRIPTION: PFCT 002-17
INJECTION VOLUME: 20 **SEQUENCE**: C:\CLASS-VP\Sequence\2017\Síntese\Frederico Branco\CLAE 02 - 20171113.seq **VIAL:** 3
METHOD: C:\CLASS-VP\Methods\2017\Sintese 1\Frederico Branco\Difluorofeniltriazol 1.met
FILE NAME: C:\CLASS-VP\Data\2017\Síntese\Frederico Branco\11-17\PFCT 002-17 rep2-Rep1
SAMPLE ID: PFCT 002-17 rep2 **ACQUIRED:** 13/11/2017 22:19:08 **PRINTED:** 14/11/2017 10:59:14
ANALISTA: Leandro/Thiago

Spectrum at time 13.33 min.

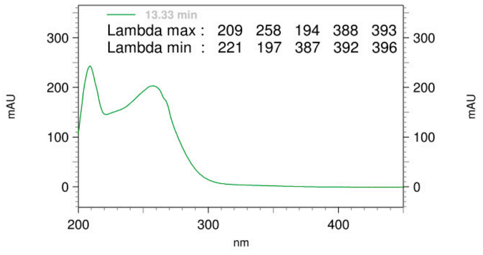

1-(2,4-difluorophenyl)-2-(2-nitro-1*H*-imidazol-1-yl)ethanol (9)

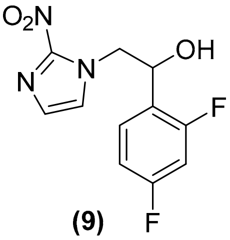

(9)

Compound Spectrum SmartFormula Report

Analysis Info

Acquisition Date 3/9/2016 1:57:03 PM

Analysis Name D:\Data\2016\SINTESE 1\INFUSAO\81193 - PRCH 015-16.d
Method Tune_pos_Standard.m
Sample Name 81193 - PRCH 015-16
Comment

Operator BDAL@DE
Instrument compact 8255754.10035

Acquisition Parameter

Source Type	ESI	Ion Polarity	Positive	Set Nebulizer	0.4 Bar
Focus	Not active	Set Capillary	4500 V	Set Dry Heater	200 °C
Scan Begin	50 m/z	Set End Plate Offset	-300 V	Set Dry Gas	4.0 l/min
Scan End	1500 m/z	Set Charging Voltage	2000 V	Set Divert Valve	Source
		Set Corona	0 nA	Set APCI Heater	0 °C

+MS, 0.0-1.0min #2-57

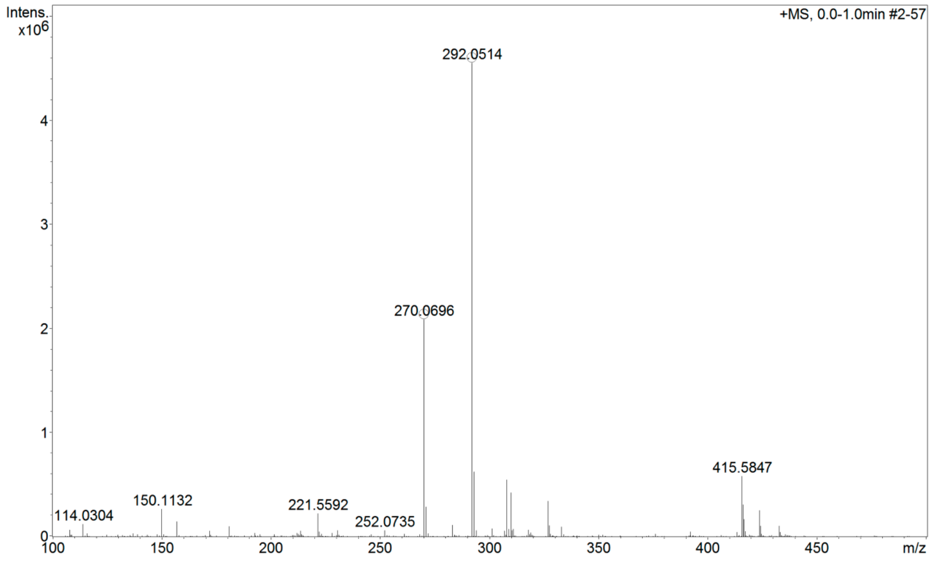

Meas. m/z	#	Ion Formula	m/z	err [ppm]	Mean err [ppm]	rdb	N-Rule	e⁻ Conf	mSigma	Std I	Std Mean m/z	Std I VarNorm	Std m/z Diff	Std Comb Dev
270.069570	1	C11H10F2N3O3	270.068474	-4.1	-3.9	7.5	ok	even	2.7	4.9	n.a.	n.a.	n.a.	n.a.
	2	C7H6F2N9O	270.065789	-14.0	-16.5	8.5	ok	even	16.2	33.8	n.a.	n.a.	n.a.	n.a.
	3	C16H10F2NO	270.072497	10.8	12.2	11.5	ok	even	23.9	44.2	n.a.	n.a.	n.a.	n.a.
292.051450	1	C11H9F2N3NaO3	292.050418	-3.5	-3.8	7.5	ok	even	2.9	4.9	n.a.	n.a.	n.a.	n.a.
	2	C7H5F2N9NaO	292.047733	-12.7	-15.5	8.5	ok	even	16.2	33.5	n.a.	n.a.	n.a.	n.a.
	3	C16H9F2NNaO	292.054441	10.2	11.1	11.5	ok	even	23.9	44.5	n.a.	n.a.	n.a.	n.a.

Library Search Report - ChemStation Integrator

Unknown Spectrum based on Apex

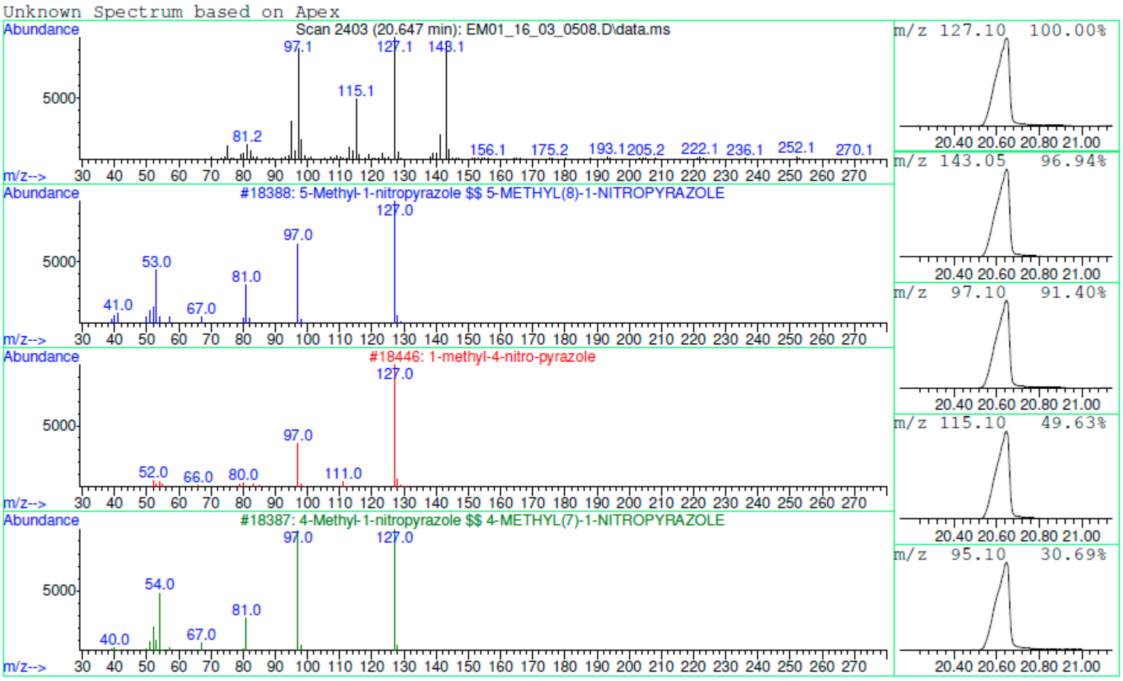

Data File: D:\Backup dos Sistemas\CG-EM-01\2016\03_MARCO_16\EM01_16_03_0508.D
Sample : PRCH014-16

Peak Number: 1 at 20.647 min Area: 176182145 Area % 100.00

The 3 best hits from each library.	Ref\#	CAS\#	Qual
C:\Database\WILEY275.L			
1 5-Methyl-1-nitropyrazole $$ 5-ME...	18388	031163-85-6	38
2 1-methyl-4-nitro-pyrazole	18446	000000-00-0	35
3 4-Methyl-1-nitropyrazole $$ 4-ME...	18387	038858-82-1	25

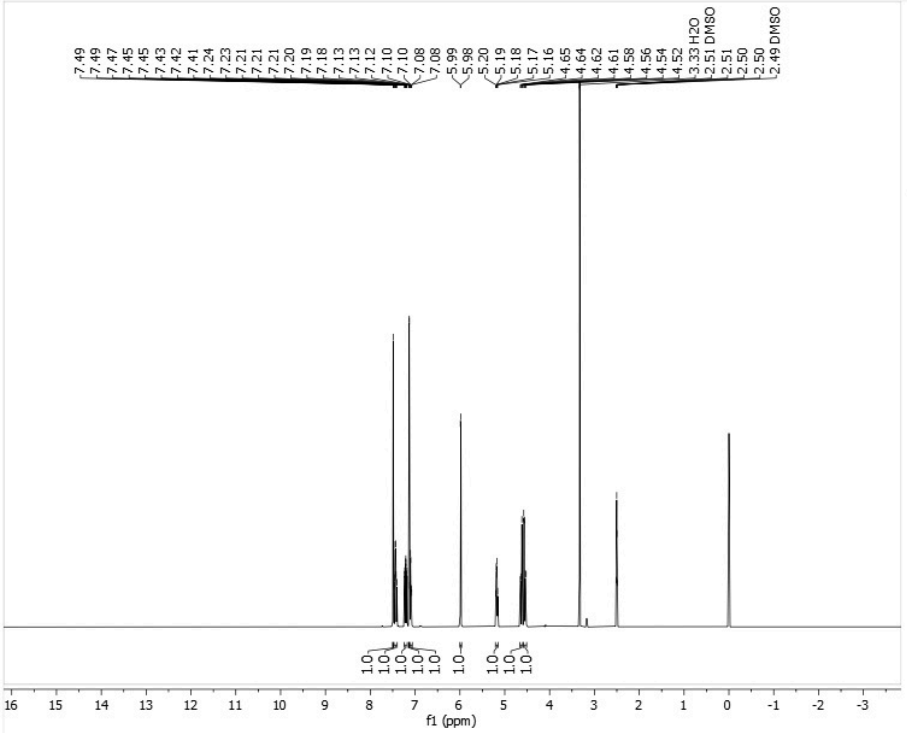

	Parameter	Value
1	Origin	Bruker BioSpin GmbH
2	Owner	nmr
3	Instrument	spect
4	Solvent	DMSO
5	Temperature	298.0
6	Pulse Sequence	zg30
7	Experiment	1D
8	Probe	5 mm CPPBBO BB-1H/ 19F/ D Z-GRD Z122623/ 0041
9	Number of Scans	32
10	Receiver Gain	163.2
11	Relaxation Delay	1.0000
12	Pulse Width	11.5000
13	Acquisition Time	4.0894
14	Acquisition Date	2016-03-09T17:17:00
15	Modification Date	2016-03-09T17:17:04
16	Spectrometer Frequency	400.15
17	Spectral Width	8012.8
18	Lowest Frequency	-1538.0
19	Nucleus	1H
20	Acquired Size	32768
21	Spectral Size	65536
22	Digital Resolution	0.12

^{1}H NMR (400 MHz, DMSO-d_6) δ 7.49 (d, J = 1.0 Hz, 2H), 7.44 (td, J = 8.6, 6.9 Hz, 2H), 7.21 (ddd, J = 10.7, 9.4, 2.5 Hz, 1H), 7.13 (d, J = 1.0 Hz, 1H), 7.14 – 7.05 (m, 1H), 5.98 (d, J = 4.9 Hz, 1H), 5.18 (dt, J = 8.8, 4.6 Hz, 2H), 4.63 (dd, J = 13.6, 4.3 Hz, 1H), 4.55 (dd, J = 13.6, 7.7 Hz, 1H).

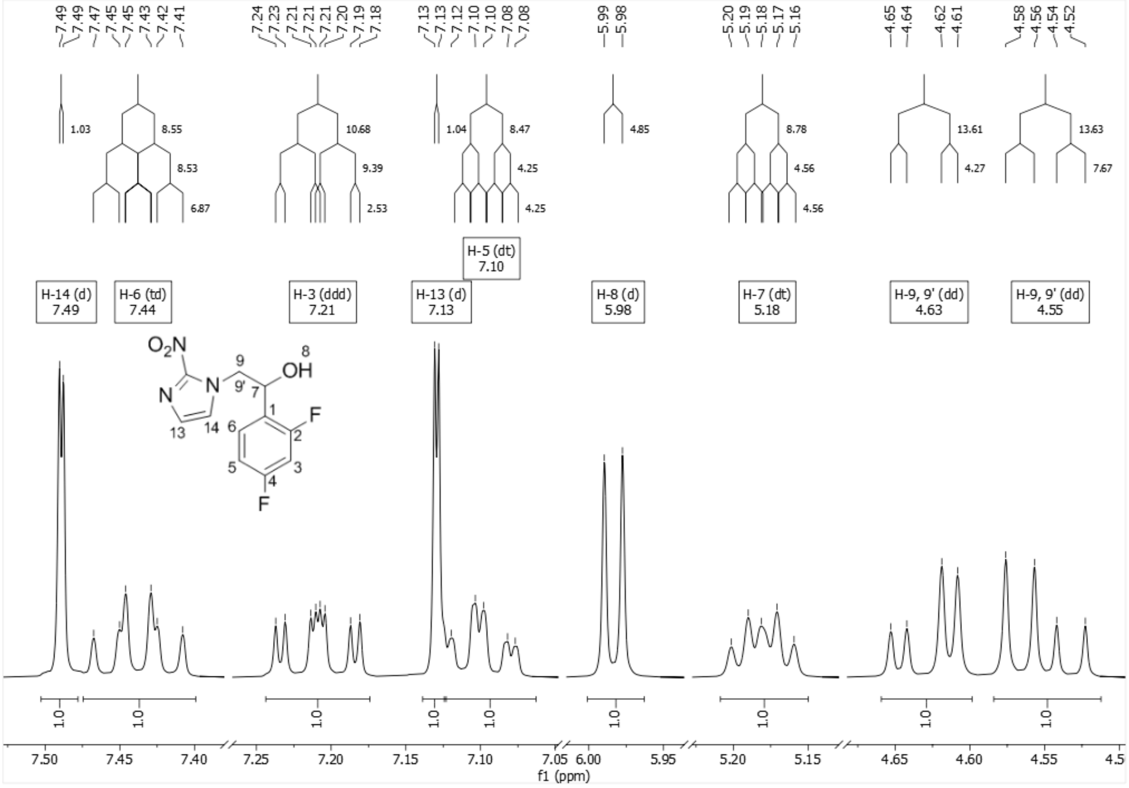

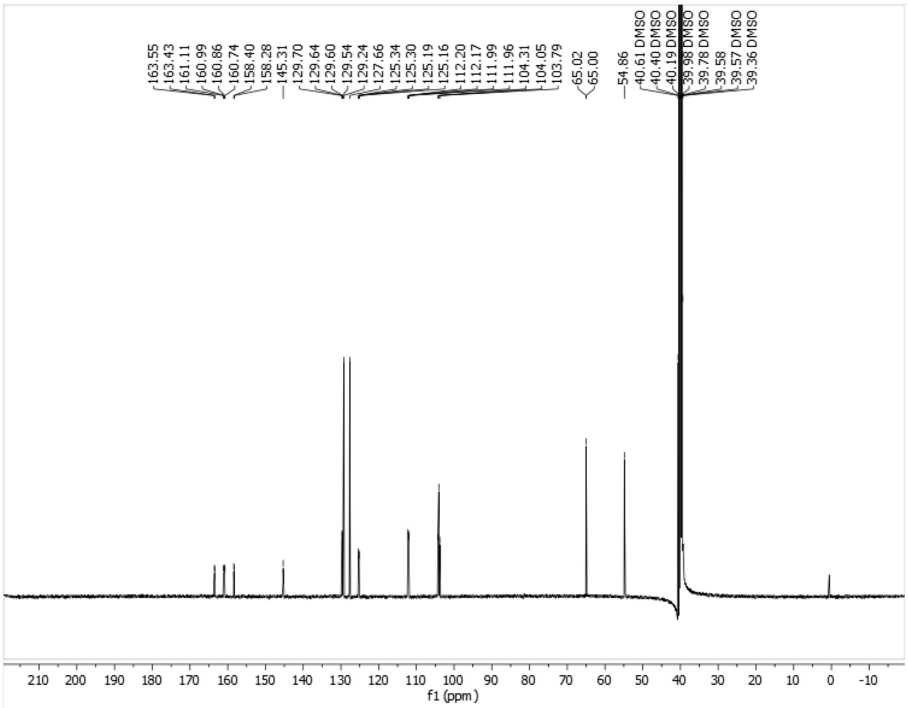

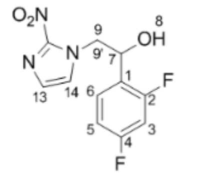

Parameter	Value
1 Origin	Bruker BioSpin GmbH
2 Owner	nmr
3 Instrument	spect
4 Solvent	DMSO
5 Temperature	298.0
6 Pulse Sequence	zgpg30
7 Experiment	1D
8 Probe	5 mm CPPBBO BB-1H/ 19F/ D Z-GRD Z122623/ 0041
9 Number of Scans	2048
10 Receiver Gain	66.7
11 Relaxation Delay	1.0000
12 Pulse Width	10.0000
13 Acquisition Time	1.3631
14 Acquisition Date	2016-03-09T18:41:00
15 Modification Date	2016-03-10T08:13:50
16 Spectrometer Frequency	100.63
17 Spectral Width	24038.5
18 Lowest Frequency	-1957.5
19 Nucleus	13C
20 Acquired Size	32768
21 Spectral Size	65536
22 Digital Resolution	0.37

^{13}C NMR (101 MHz, DMSO-d_6) δ 162.27 (dd, J = 246.1, 12.3 Hz), 159.57 (dd, J = 247.3, 12.4 Hz), 145.31, 129.62 (dd, J = 10.0, 6.1 Hz), 129.24, 127.66, 125.25 (dd, J = 14.4, 3.6 Hz), 112.08 (dd, J = 21.2, 3.5 Hz), 104.05 (t, J = 26.0 Hz), 65.01 (d, J = 1.4 Hz), 54.86.

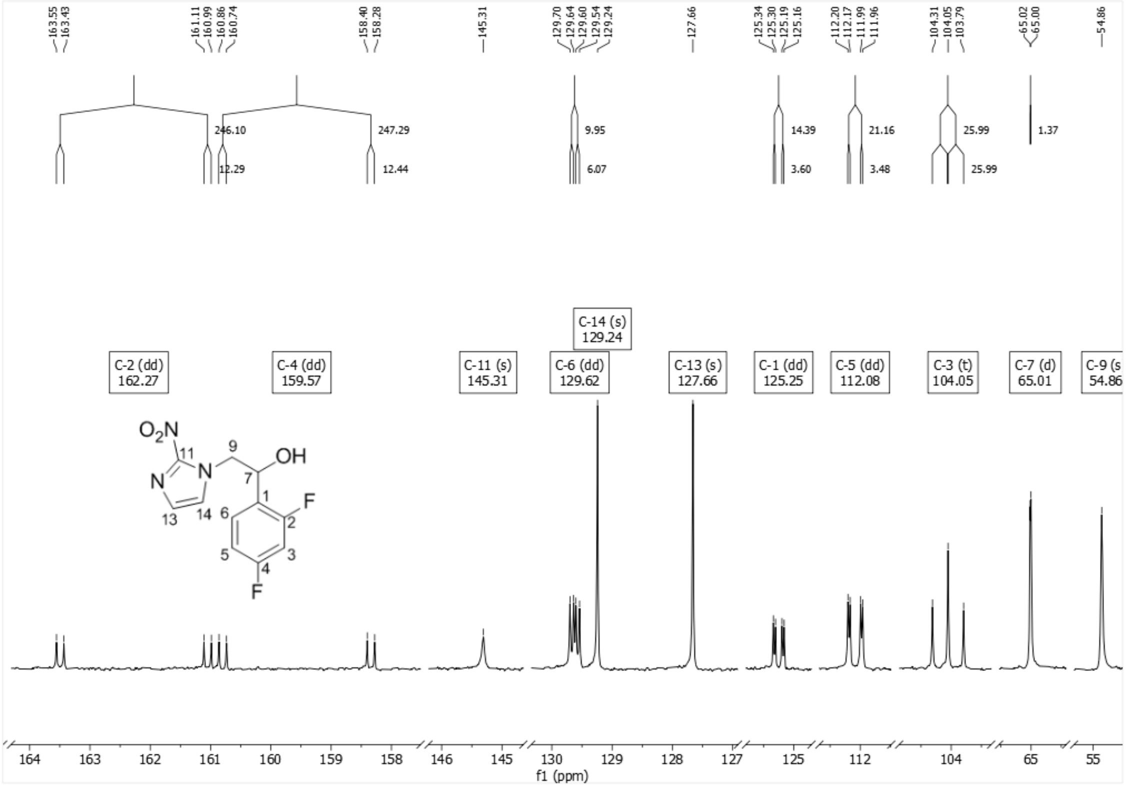

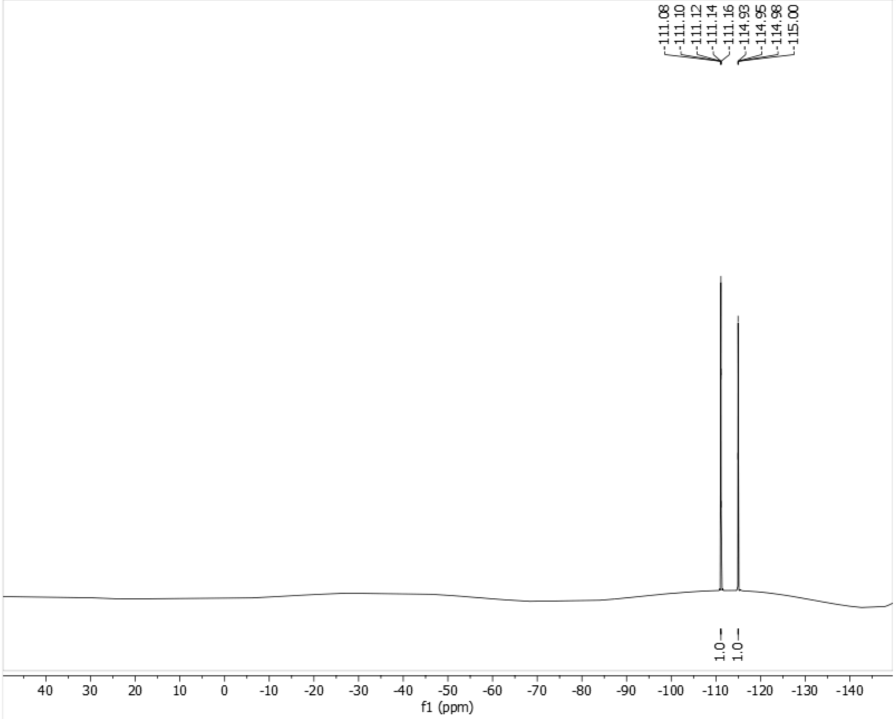

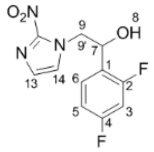

	Parameter	Value
1	Origin	Bruker BioSpin GmbH
2	Owner	nmr
3	Instrument	spect
4	Solvent	DMSO
5	Temperature	298.0
6	Pulse Sequence	zg30
7	Experiment	1D
8	Probe	5 mm CPPBBO BB-1H/ 19F/ D Z-GRD Z122623/ 0041
9	Number of Scans	32
10	Receiver Gain	32.3
11	Relaxation Delay	1.0000
12	Pulse Width	15.0000
13	Acquisition Time	0.8738
14	Acquisition Date	2016-03-09T18:45:00
15	Modification Date	2016-03-09T18:45:58
16	Spectrometer Frequency	376.52
17	Spectral Width	75000.0
18	Lowest Frequency	-56325.9
19	Nucleus	19F
20	Acquired Size	65536
21	Spectral Size	131072
22	Digital Resolution	0.57

^{19}F NMR (377 MHz, DMSO-d_6) δ -111.07 – -111.17 (m), -114.97 (dd, J = 18.1, 8.2 Hz).

Amostra: PRCH 015/16

Perfil Cromatográfico (λ = 225 nm)

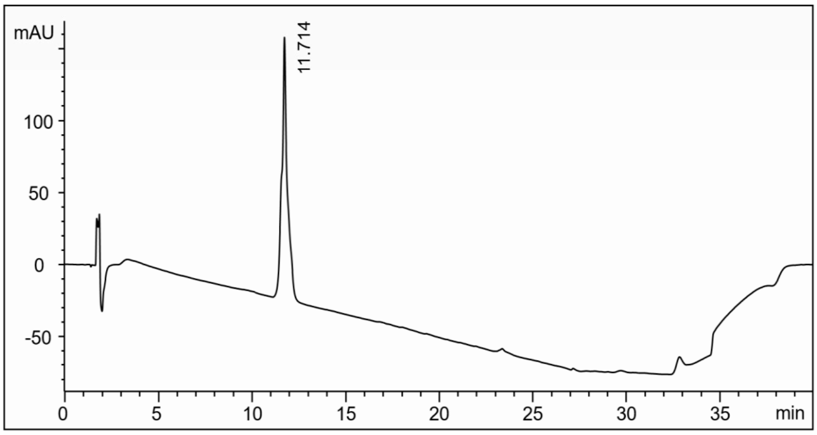

PRCH 015/16 (225 nm)

Tr (min)	Área	%
11,71	3754,9	**100,0**
	3754,9	**100,0**

Perfil Cromatográfico (λ = 245 nm)

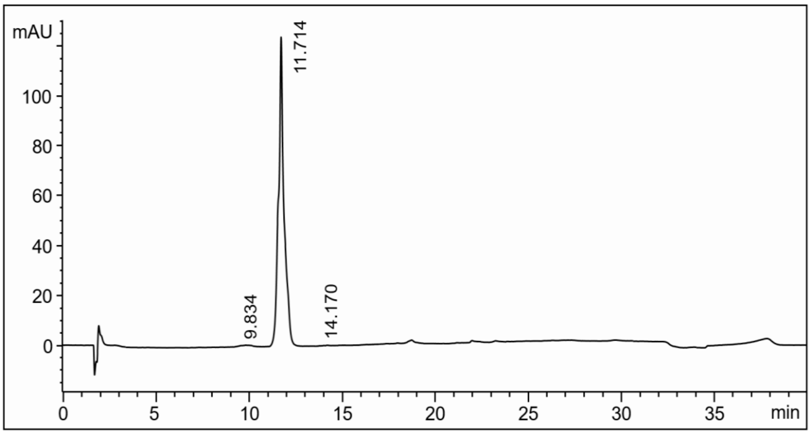

PRCH 015/16 (245 nm)

Tr (min)	Área	%
9,83	32,109	1,24
11,71	2561,4	**98,56**
14,17	5,44	0,21
	2598,949	100,0

Perfil Cromatográfico (λ = 265 nm)

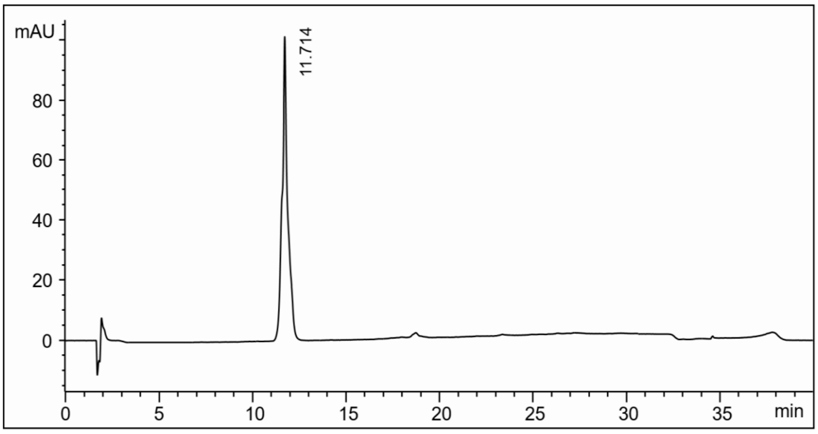

PRCH 015/16 (265 nm)

Tr	Área	%
11,71	2096,5	**100,0**
	2096,5	100,0

Perfil Cromatográfico (λ = 290 nm)

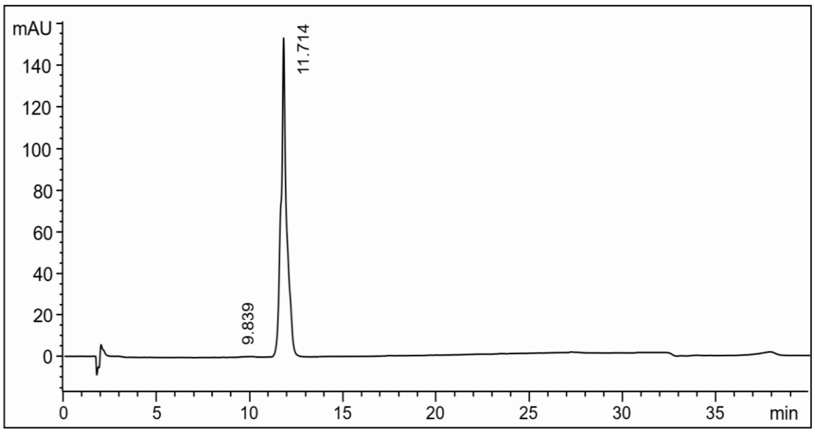

PRCH 015/16 (290 nm)

Tr	Área	%
9,83	12,375	0,39
11,71	3184,3	**99,61**
	3196,7	100,0

Espectro de UV (Tr = 11,71 min)

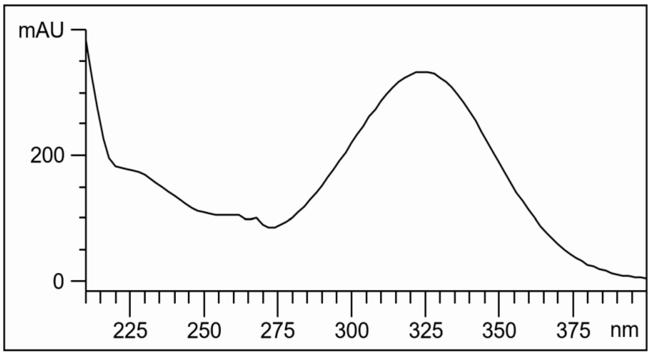

1-(2,4-difluorphenyl)-2-(1 *H*-1,2,4-triazol-1-yl)ethanol (10)

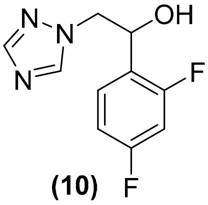

(10)

Compound Spectrum SmartFormula Report

Analysis Info

		Acquisition Date	3/3/2016 8:35:43 AM	
Analysis Name	D:\Data\2016\SINTESE 1\INFUSAO\80852 - PRCH 021-15.d			
Method	Tune_pos_Standard.m	Operator	BDAL@DE	
Sample Name	80852 - PRCH 021-15	Instrument	compact	8255754.10035
Comment				

Acquisition Parameter

Source Type	ESI	Ion Polarity	Positive	Set Nebulizer	0.4 Bar
Focus	Not active	Set Capillary	4500 V	Set Dry Heater	200 °C
Scan Begin	50 m/z	Set End Plate Offset	-300 V	Set Dry Gas	4.0 l/min
Scan End	1500 m/z	Set Charging Voltage	2000 V	Set Divert Valve	Source
		Set Corona	0 nA	Set APCI Heater	0 °C

+MS, 0.1-1.0min #3-56

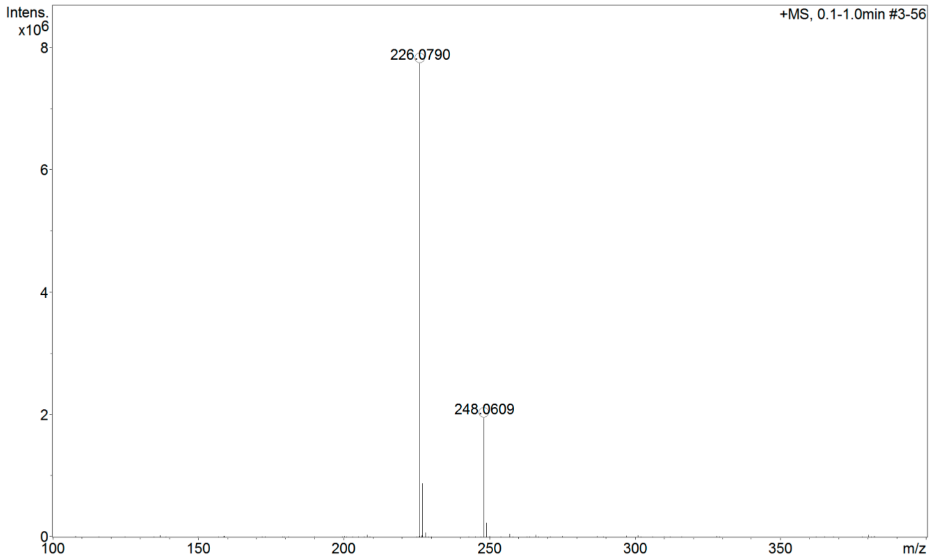

Meas. m/z	#	Ion Formula	m/z	err [ppm]	Mean err [ppm]	rdb	N-Rule	e⁻ Conf	mSigma	Std I	Std Mean m/z	Std I VarNorm	Std m/z Diff	Std Comb Dev
226.078954	1	C10H10F2N3O	226.078645	-1.4	-1.1	6.5	ok	even	4.0	7.5	n.a.	n.a.	n.a.	n.a.
248.060944	1	C10H9F2N3NaO	248.060589	-1.4	-1.2	6.5	ok	even	0.2	0.5	n.a.	n.a.	n.a.	n.a.

Library Search Report - ChemStation Integrator

Unknown Spectrum based on Apex

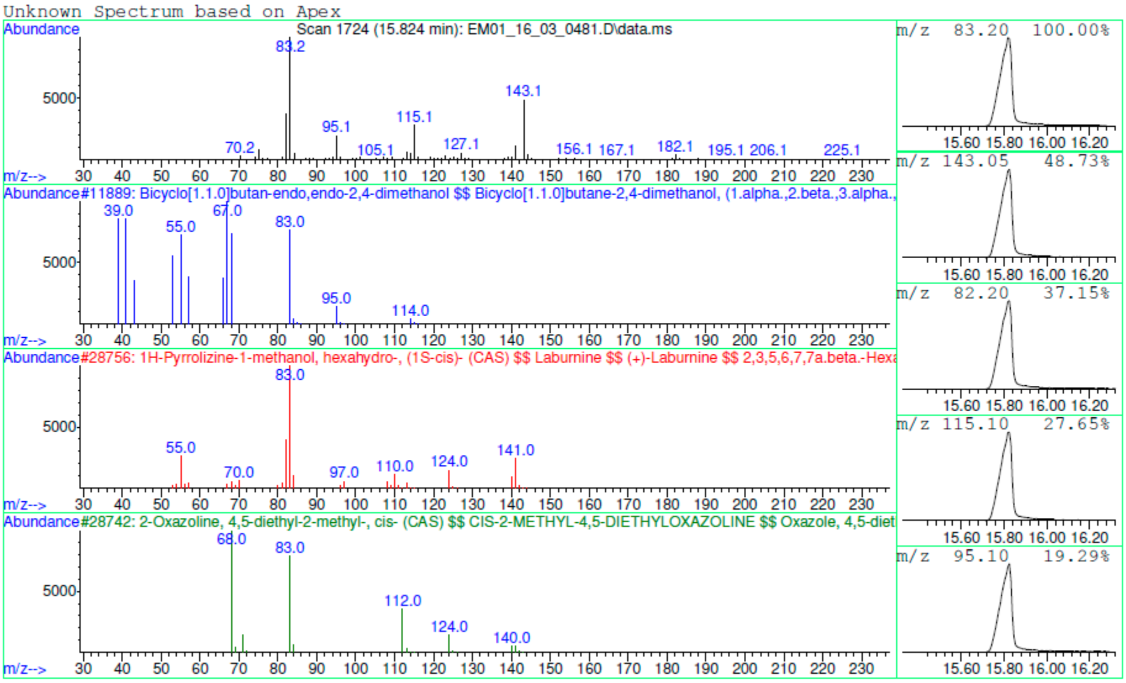

Data File: D:\Backup dos Sistemas\CG-EM-01\2016\03_MARCO_16\EM01_16_03_0481.D
Sample : PRCH 021-15

Peak Number: 1 at 15.824 min Area: 152076027 Area % 100.00

The 3 best hits from each library.	Ref\#	CAS\#	Qual
C:\Database\WILEY275.L			
1 Bicyclo[1.1.0]butan-endo,endo-2,...	11889	073688-08-1	32
2 1H-Pyrrolizine-1-methanol, hexah...	28756	003348-73-0	23
3 2-Oxazoline, 4,5-diethyl-2-methy...	28742	025943-07-1	9

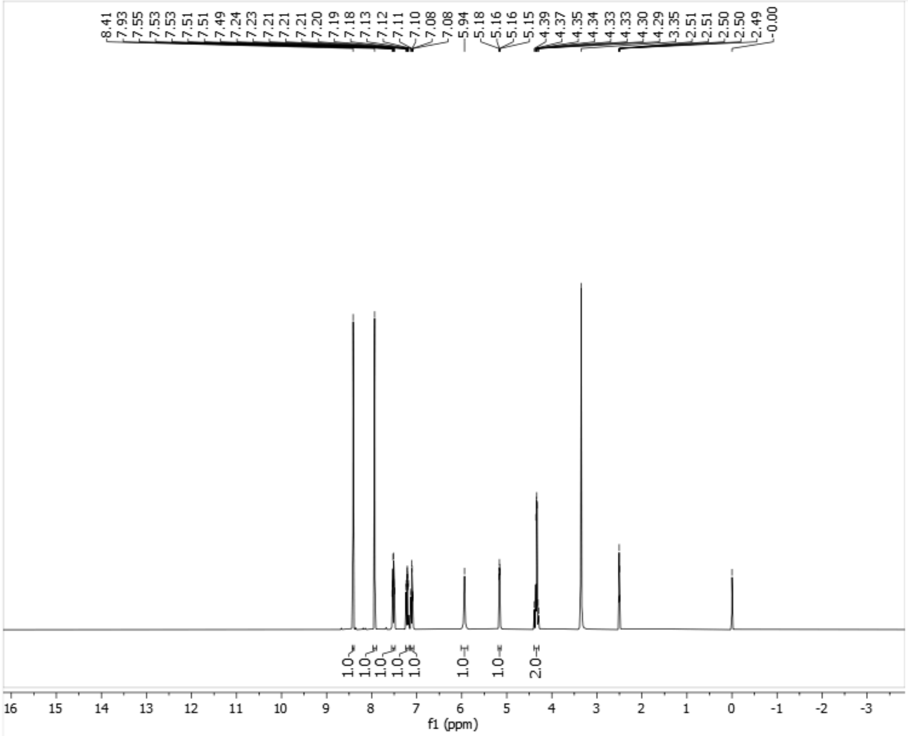

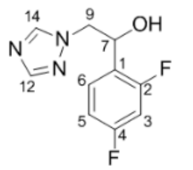

Parameter	Value
1 Origin	Bruker BioSpin GmbH
2 Owner	nmr
3 Instrument	spect
4 Solvent	DMSO
5 Temperature	298.0
6 Pulse Sequence	zg30
7 Experiment	1D
8 Probe	5 mm CPPBBO BB-1H/ 19F/ D Z-GRD Z122623/ 0041
9 Number of Scans	32
10 Receiver Gain	116.9
11 Relaxation Delay	1.0000
12 Pulse Width	11.5000
13 Acquisition Time	4.0894
14 Acquisition Date	2016-03-03T10:46:00
15 Modification Date	2016-03-03T10:47:48
16 Spectrometer Frequency	400.15
17 Spectral Width	8012.8
18 Lowest Frequency	-1537.6
19 Nucleus	1H
20 Acquired Size	32768
21 Spectral Size	65536
22 Digital Resolution	0.12

^{1}H NMR (400 MHz, DMSO-d_6) δ 8.41 (s, 1H), 7.93 (s, 1H), 7.52 (td, J = 8.6, 7.0 Hz, 2H), 7.21 (ddd, J = 10.8, 9.5, 2.5 Hz, 2H), 7.10 (td, J = 8.5, 2.2 Hz, 2H), 5.94 (s, 1H), 5.16 (dd, J = 7.2, 5.0 Hz, 2H), 4.41 – 4.27 (m, 3H).

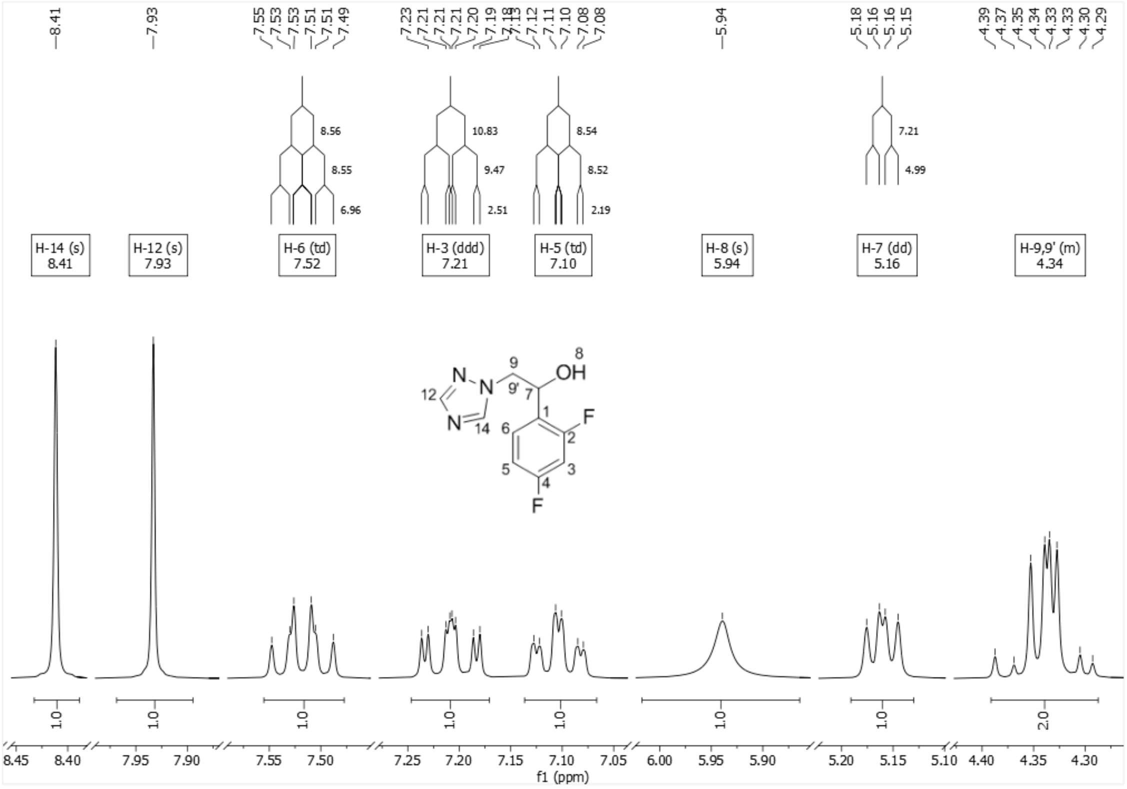

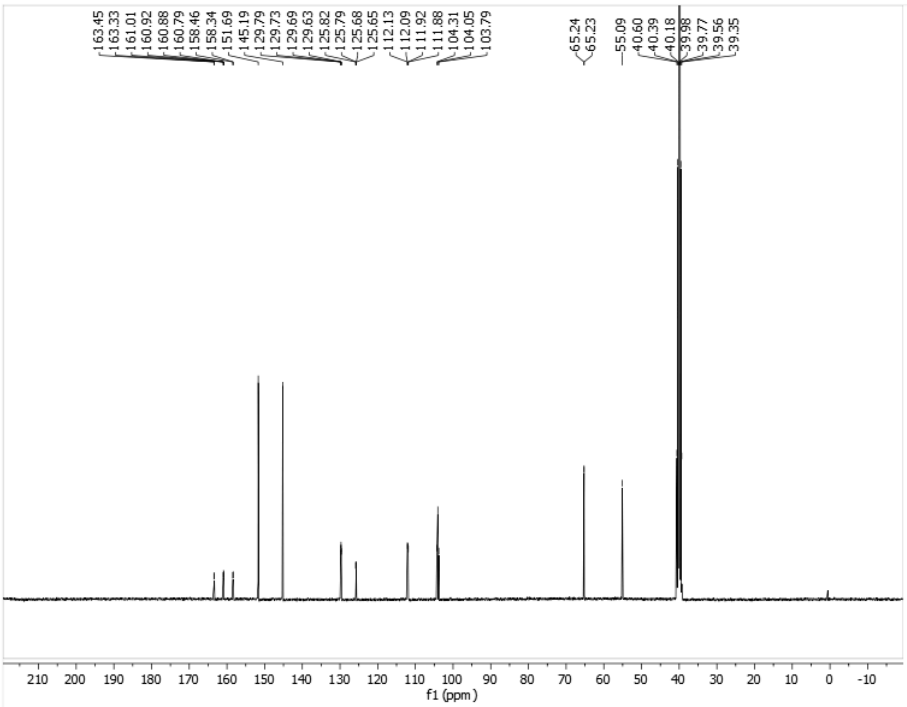

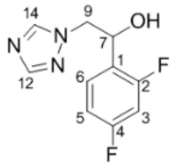

Parameter	Value
1 Origin	Bruker BioSpin GmbH
2 Owner	nmr
3 Instrument	spect
4 Solvent	DMSO
5 Temperature	298.0
6 Pulse Sequence	zgpg30
7 Experiment	1D
8 Probe	5 mm CPPBBO BB-1H/ 19F/ D Z-GRD Z122623/ 0041
9 Number of Scans	1024
10 Receiver Gain	66.7
11 Relaxation Delay	1.0000
12 Pulse Width	10.0000
13 Acquisition Time	1.3631
14 Acquisition Date	2016-03-03T07:55:00
15 Modification Date	2016-03-03T09:12:04
16 Spectrometer Frequency	100.63
17 Spectral Width	24038.5
18 Lowest Frequency	-1957.5
19 Nucleus	13C
20 Acquired Size	32768
21 Spectral Size	65536
22 Digital Resolution	0.37

^{13}C NMR (101 MHz, DMSO-d_6) δ 163.54 – 160.83 (m), 160.97 – 158.25 (m), 151.69, 145.19, 129.71 (dd, J = 9.9, 6.2 Hz), 125.73 (dd, J = 14.2, 3.6 Hz), 112.01 (dd, J = 21.1, 3.5 Hz), 104.05 (t, J = 26.0 Hz), 65.24 (d, J = 1.3 Hz), 55.09.

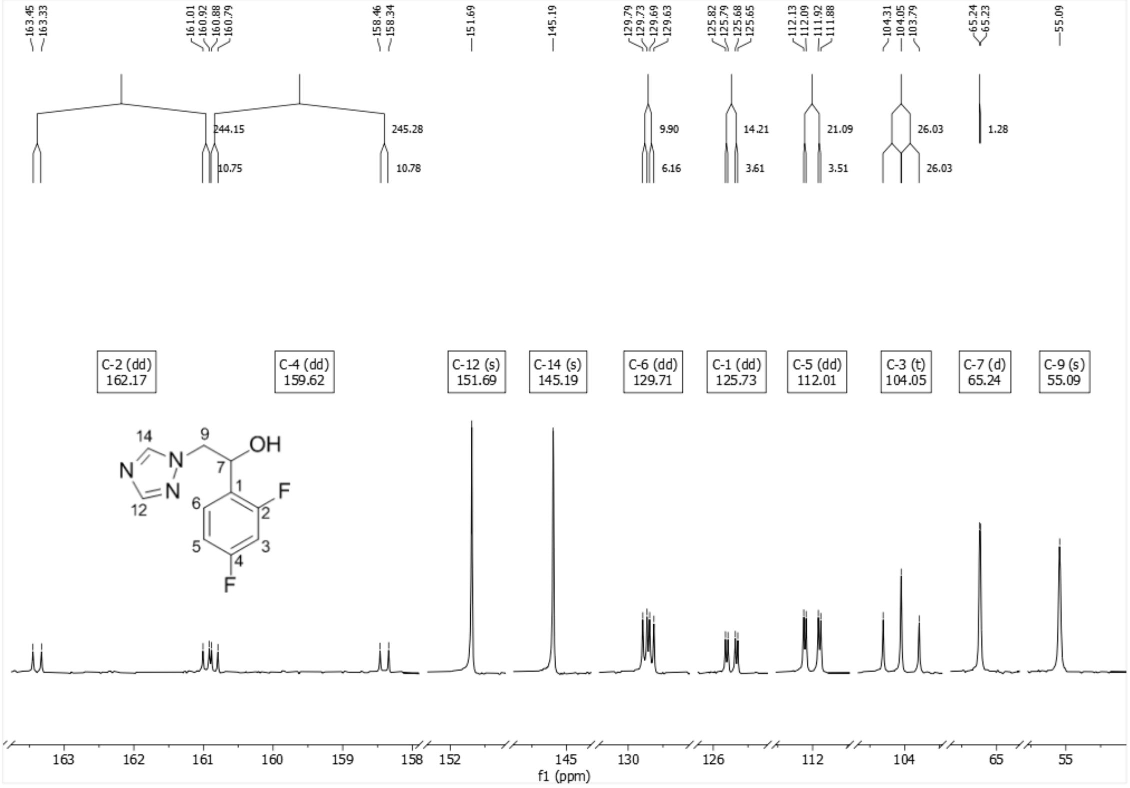

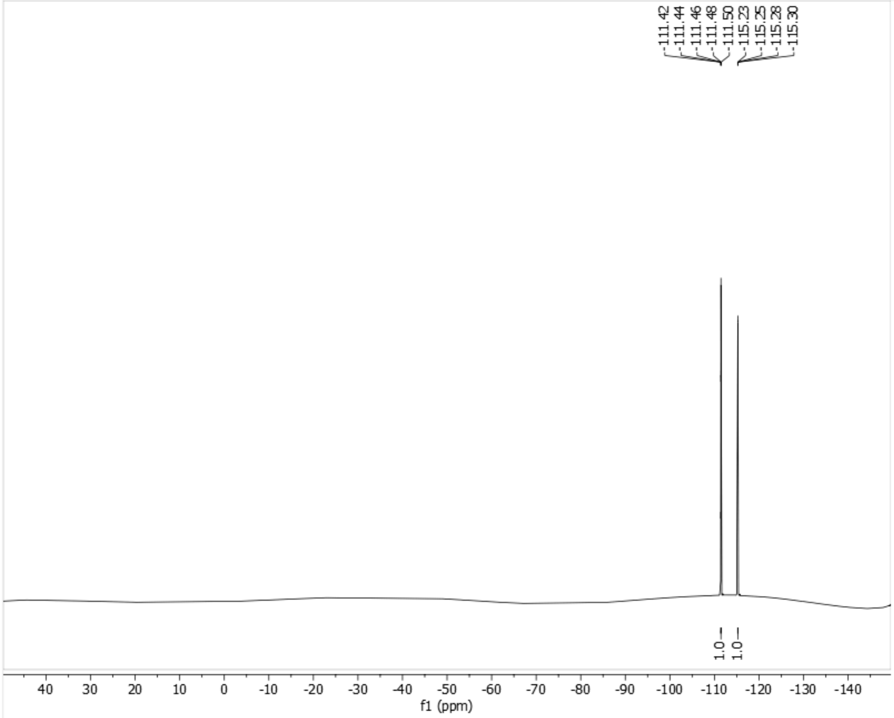

^{19}F NMR (377 MHz, DMSO-d_6) δ -111.41 – -111.51 (m), -115.27 (dd, J = 18.1, 8.3 Hz).

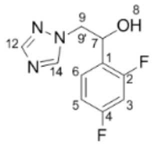

	Parameter	Value
1	Origin	Bruker BioSpin GmbH
2	Owner	nmr
3	Instrument	spect
4	Solvent	DMSO
5	Temperature	298.0
6	Pulse Sequence	zg30
7	Experiment	1D
8	Probe	5 mm CPPBBO BB-1H/ 19F/ D Z-GRD Z122623/ 0041
9	Number of Scans	32
10	Receiver Gain	32.3
11	Relaxation Delay	1.0000
12	Pulse Width	15.0000
13	Acquisition Time	0.8738
14	Acquisition Date	2016-03-03T08:28:00
15	Modification Date	2016-03-03T08:28:22
16	Spectrometer Frequency	376.52
17	Spectral Width	75000.0
18	Lowest Frequency	-56325.9
19	Nucleus	19F
20	Acquired Size	65536
21	Spectral Size	131072
22	Digital Resolution	0.57

LaQAFA
Laboratório de Química Analítica Fundamental e Aplicada

Amostra: PRCH 021/15

Perfil Cromatográfico (λ = 225 nm)

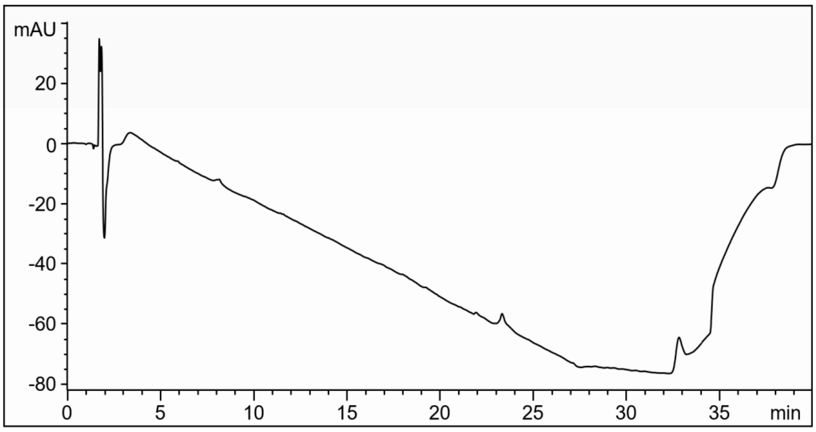

Perfil Cromatográfico (λ = 245 nm)

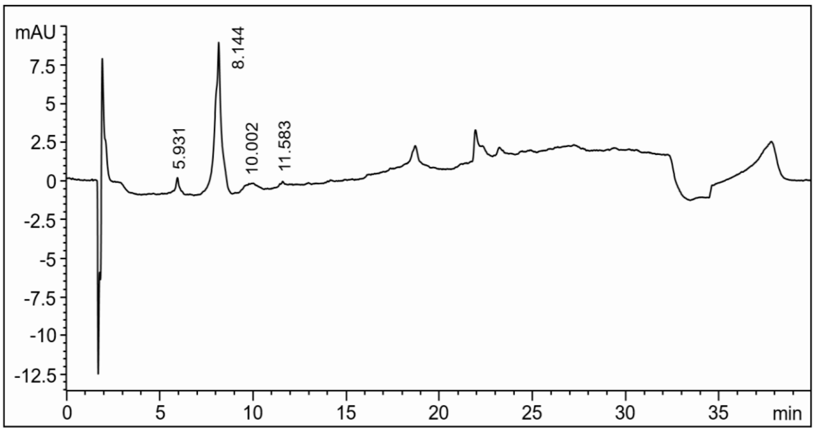

PRCH 021/15 (245 nm)

Tr (min)	Área	% Área
5,93	17,46	5,91
8,14	239,66	**81,18**
10,00	26,99	9,14
11,58	11,10	3,76
	295,21	100,0

LaQAFA
Laboratório de Química Analítica Fundamental e Aplicada

Perfil Cromatográfico (λ = 265 nm)

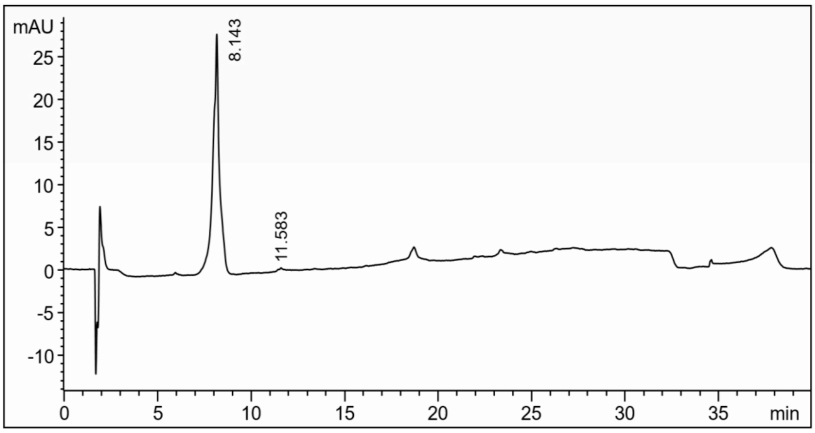

PRCH 021/15 (265 nm)

Tr (min)	Área	% Área
8,14	698,55	**98,70**
11,58	9,235	1,30
	707,785	100,0

Perfil Cromatográfico (λ = 290 nm)

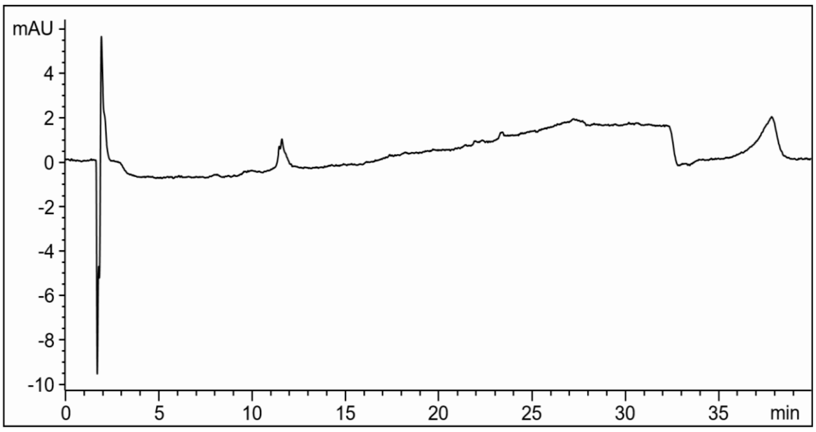

Espectro de UV (Tr = 8,14 min)

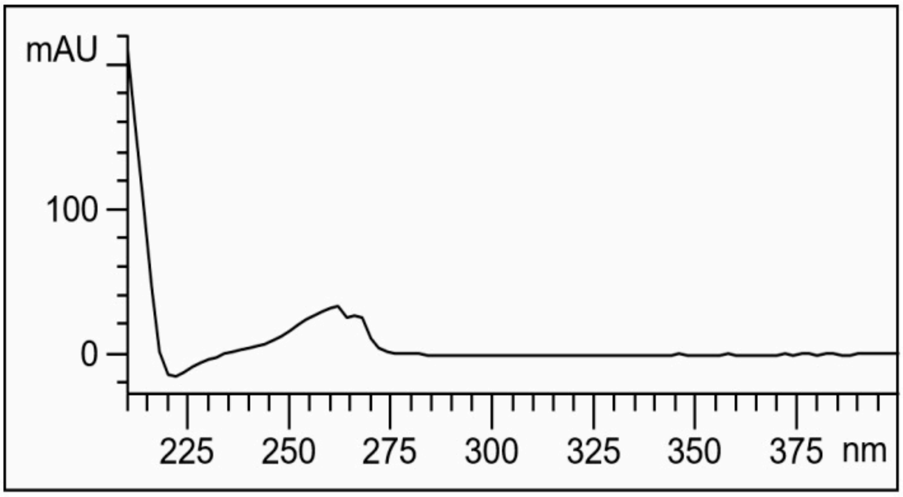

Supplement: Supplementary file 1 [file molecules-28-07461-s001.zip › molecules-2647909-SI.pdf]
